# Pyrazolate-Bridged NHC Cyclometalated [Pt_2_] Complexes and [Pt_2_Ag(PPh_3_)]^+^ Clusters in Electroluminescent Devices

**DOI:** 10.1021/acs.inorgchem.4c00105

**Published:** 2024-04-08

**Authors:** Jorge Roy, Michele Forzatti, Lorenzo Arnal, Antonio Martín, Sara Fuertes, Daniel Tordera, Violeta Sicilia

**Affiliations:** † Departamento de Química Inorgánica, Facultad de Ciencias, Instituto de Síntesis Química y Catálisis Homogénea (ISQCH), CSIC - Universidad de Zaragoza, Pedro Cerbuna 12, Zaragoza 50009, Spain; ‡ Instituto de Ciencia Molecular, Universidad de Valencia, C/Catedrático J. Beltran, 2, Paterna 46980, Spain; § Departamento de Química Inorgánica, Escuela de Ingeniería y Arquitectura de Zaragoza, Instituto de Síntesis Química y Catálisis Homogénea (ISQCH), CSIC - Universidad de Zaragoza, Campus Río Ebro, Edificio Torres Quevedo, Zaragoza 50018, Spain

## Abstract

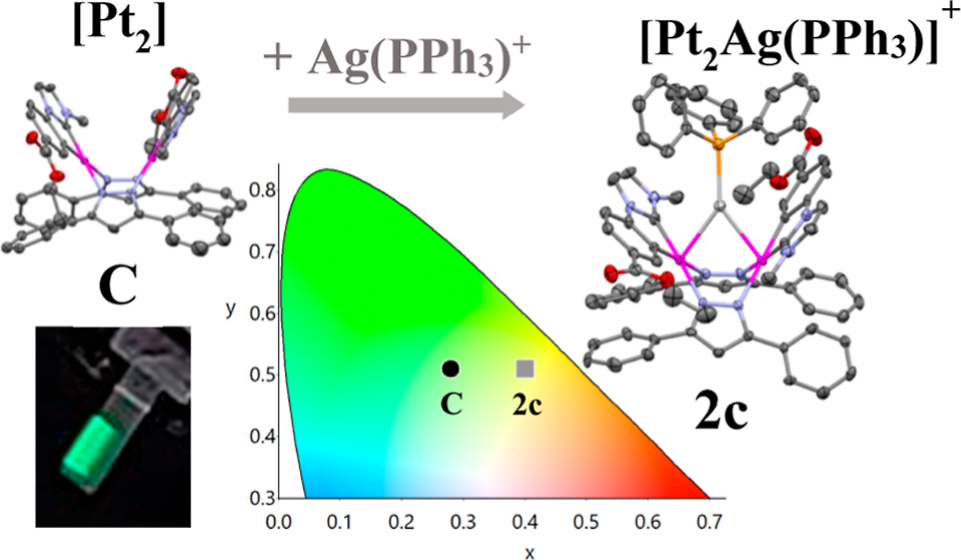

The ionic transition metal complexes (iTMCs) [{Pt(C^∧^C*)(μ-Rpz)}_2_Ag(PPh_3_)]X (HC^∧^C* = 1-(4-(ethoxycarbonyl)phenyl)-3-methyl-1*H*-imidazole-2-ylidene, X = ClO_4_/PF_6;_ Rpz = pz **1a/2a**, 4-Mepz **1b/2b**, and 3,5-dppz **1c/2c**) were prepared from the neutral [{Pt(C^∧^C*)(μ-Rpz)}_2_] (Rpz = pz **A**, 4-Mepz **B**, and 3,5-dppz **C**) and fully characterized. The “Ag(PPh_3_)” fragment is in between the two square-planar platinum units in an “open book” disposition and bonded through two Pt–Ag donor–acceptor bonds, as shown by X-ray diffraction (d_Pt–Ag_ ∼ 2.78 Å, **1a**–**1c**). ^195^Pt{^1^H} and ^31^P{^1^H} NMR confirmed that these solid-state structures remain in solution. Photoluminescence studies and theoretical calculations on **1a**, were performed. The diphenylpyrazolate derivatives show the highest photoluminescence quantum yield (PLQY) in the solid state. Therefore, **2c** and its neutral precursor **C** were selected as active materials on light-emitting devices. OLEDs fabricated with **C** showed a turn-on voltage of 3.2 V, a luminance peak of 21,357 cd m^–2^ at 13 V, and a peak current efficiency of 28.8 cd A^–1^ (9.5% EQE). They showed a lifetime t_50_ of 15.7 h. OLEDs using **2c** showed a maximum luminance of 114 cd m^–2^, while LECs exhibited a maximum luminance of 20 cd m^–2^ and a current efficiency of around 0.2 cd A^–1^, with a t_50_ value of 50 min.

## Abstract 2

The [Pt_2_Ag(PPh_3_)] ^+^ clusters [{Pt(C∧C*)(μ-Rpz)}_2_Ag(PPh_3_)]^+^ were available from the neutral [Pt_2_] complexes. The incorporation of a [Ag(PPh_3_)] ^+^ fragment allows for iTMCs with tunable phosphorescence with respect to the precursors. The more efficient emitters, **C** and **2c**, were used as active materials in EL devices.

## Introduction

The first organic light-emitting diode (OLED) was reported in 1987 by Tang and Van Slyke.^1^ It consisted of two layers sandwiched between indium tin oxide (ITO) and Mg– Ag alloy electrodes: 1-bis{4-[di(*p*-tolyl)amino]-phenyl}cyclohexane (TAPC) as the hole transport layer and tri(8-hydroxyquinolinolate)aluminum (Alq_3_) as the emitting material. Since then, OLED performance has experienced great progress, from improved architectures to more efficient emitter materials and charge carriers, driven by its great potential in fabricating advanced displays.^2^ One of the most crucial components in these devices is the light-emitting layer (EML). The incorporation of phosphorescent heavy-metal complexes, mainly those of the third-row [Re^+^, Os^2+^, Ir^3+^ (5d^6^), Pt^2+^, and Au^3+^ (5d^8^)], with high spin–orbit coupling constants as dopants of the fluorescent organic materials, enables triplet excitons to be harvested in the device in addition to singlets from pure organic materials. This allows phosphorescent organic light-emitting diodes (PhOLEDs) to reach a theoretical internal quantum efficiency (IQE) of up to 100%, while only 25% IQE can be reached in pure organic OLEDs.^3^ This idea was born independently in the laboratories of Ma and Che^4^ and Baldo and Thompson^5^ in 1998 using [Os(CN)_2_(PPh_3_)_2_(L^∧^L)] (L^∧^L = 4,4′-diphenyl-2,2′-bipyridine) and [Pt(OEP)] (OEP = octaethylporphyrin), respectively.

To date, most work on Pt(II) involves mononuclear complexes and has been subject to several revisions.^3,6−13^ In this type of compounds, the tuning of emission properties relies on the design of the chromophoric ligands, including their hapticity and the nature of the donor atoms. To a lesser extent, dinuclear Pt(II) complexes [Pt_2_] have also been used in the fabrication of PhOLEDs.^14−16^ In these cases, the bridging ligands also play an important role in controlling the Pt···Pt interaction and the nature of the excited states. Most of these complexes comprise two “Pt(β-diketonate)” units bridged by a tetradentate ligand, which prevents intramolecular Pt···Pt interactions.^15,17−24^ Among them, those in which the tetradentate ligand is doubly C^∧^N-cyclometalated are the most prevalent,^15,18−24^ reaching external quantum efficiencies (EQEs) above 20%, only in a few cases.^15,22,24^ Nevertheless, dinuclear complexes with a half-lantern (double decker) structure and short Pt···Pt distances have also been incorporated in PhOLEDs, obtaining EQE from 0.14 to 26.4% in the deep-red or NIR region.^25−32^ Another family comprises complexes with pyrazolate bridging ligands of general formula [{Pt(C^∧^E)(μ-Rpz)}_2_] (C^∧^E = 2-(4′,6′-difluorophenyl)pyridinato-N,C^2’^; Rpz = pyrazolate, 3-methyl-5-*tert*-butylpyrazolate, and 3,5-bis(*tert*-butyl)pyrazolate;^33^ C^∧^E = 2-(pyren-1-yl) pyridine, 2-(7-(*tert*-butyl)pyren-1-yl)pyridine, Rpz = 3,5-dimethylpyrazolate,^34^ and C^∧^E = Phenyl-NHC^35^). In the former, the smaller the bulkiness of the pyrazolate, the greater the intramolecular Pt···Pt distance and the emission energy.^33^ These complexes were used to fabricate blue, green, red, and white OLEDs with EQEs of 3.8% (blue), 6.6% (green and red), and ∼5% (white). With the latter, blue OLEDs were made with an EQE of 23.4%.^35^ Besides, PhOLEDs based on d^8^-d^10^ heteronuclear clusters containing noticeable Pt-M(I) (M = Ag and Au) contacts have also been described.^16^ Several families of tri- or tetraphosphine-supported PtAu_2_^36−38^ and PtAg_2_^39,40^ with Pt-bearing functionalized acetylides were used to prepare high-efficiency solution-processed OLEDs with EQEs up to 21.5%.

In addition, light-emitting electrochemical cells (LECs) based on a phosphorescent ionic transition metal complex (iTMC) have been largely investigated as a lower-cost alternative to OLEDs, especially those based on Ir(III) complexes.^41−43^ However, platinum-based LECs have seldom been explored, with just a few examples found in the literature.^44−48^ Most of them are polynuclear complexes of Pt(II) with cyclometalated C^∧^N or N^∧^C^∧^N/N^∧^N^∧^C pincer ligands, which exhibit emission in the red region of the visible spectrum, with just one example of green Pt(II)-based LECs reported up to date.^44^

Over the past decade, our research has been mainly focused on phosphorescent Pt(II) complexes bearing cyclometalated N-heterocyclic carbenes (C^∧^C*), which have been proven to emit with a high photoluminescence quantum yield (PLQY). In these complexes, the incorporation of electron-withdrawing substituents into the aryl cyclometalated fragment allows to obtain very efficient blue-emitters,^49−52^ among them the already reported dinuclear pyrazolate-bridged complexes [{Pt(EtO_2_C–C^∧^C*)(μ-Rpz)}_2_] (HRpz = pyrazol, 4-methylpyrazol, 3,5-dimethylpyrazol, and 3,5-diphenylpyrazol).^52^ These platinum butterfly structures exhibit, on both the ground (GS) and lowest adiabatic triplet excited state (T_1_), two conformers, the butterfly spread and the butterfly folded ones, which are characterized by long and short Pt–Pt distances, respectively. In poly(methyl methacrylate) (PMMA) films, they show intense sky-blue emissions, ^3^IL/MLCT in nature, arising from the major butterfly spread conformer. However, the emission of the 3,5-diphenylpyrazolate derivative shifts from greenish-blue to yellowish-green upon mechanical grinding. This process induces an intramolecular structural change in the GS with shortening of the Pt–Pt distances, which results in the suppression of the ^3^IL/MLCT band and the appearance of the ^3^MMLCT one.

Aiming to tune the emission color of these neutral platinum butterfly complexes and to convert them into ionic transition metal complexes (iTMCs), we took advantage of their “open book” structure and the basicity of the metal centers. As a result, herein, we report the synthesis of the iTMCs, [{Pt(EtO_2_C–C^∧^C*)(μ-Rpz)}_2_Ag(PPh_3_)]^+^ (Rpz = pz, 4-Mepz, and 3,5-dppz), which have been isolated as the ClO_4_^–^ and the PF_6_^–^ salts. The photoluminescent properties of the clusters [Pt_2_Ag(PPh_3_)]^+^ have been deeply studied. Electroluminescent (EL) devices from the more efficient emitters [{Pt(EtO_2_C–C^∧^C*)(μ-3,5-dppz)}_2_] and [{Pt(EtO_2_C–C^∧^C*)(μ-3,5-dppz)}_2_Ag(PPh_3_)]PF_6_ have been fabricated, and their performance is compared with those based on analogous active materials.

## Results and Discussion

### Synthesis and Characterization

**Scheme 1:**
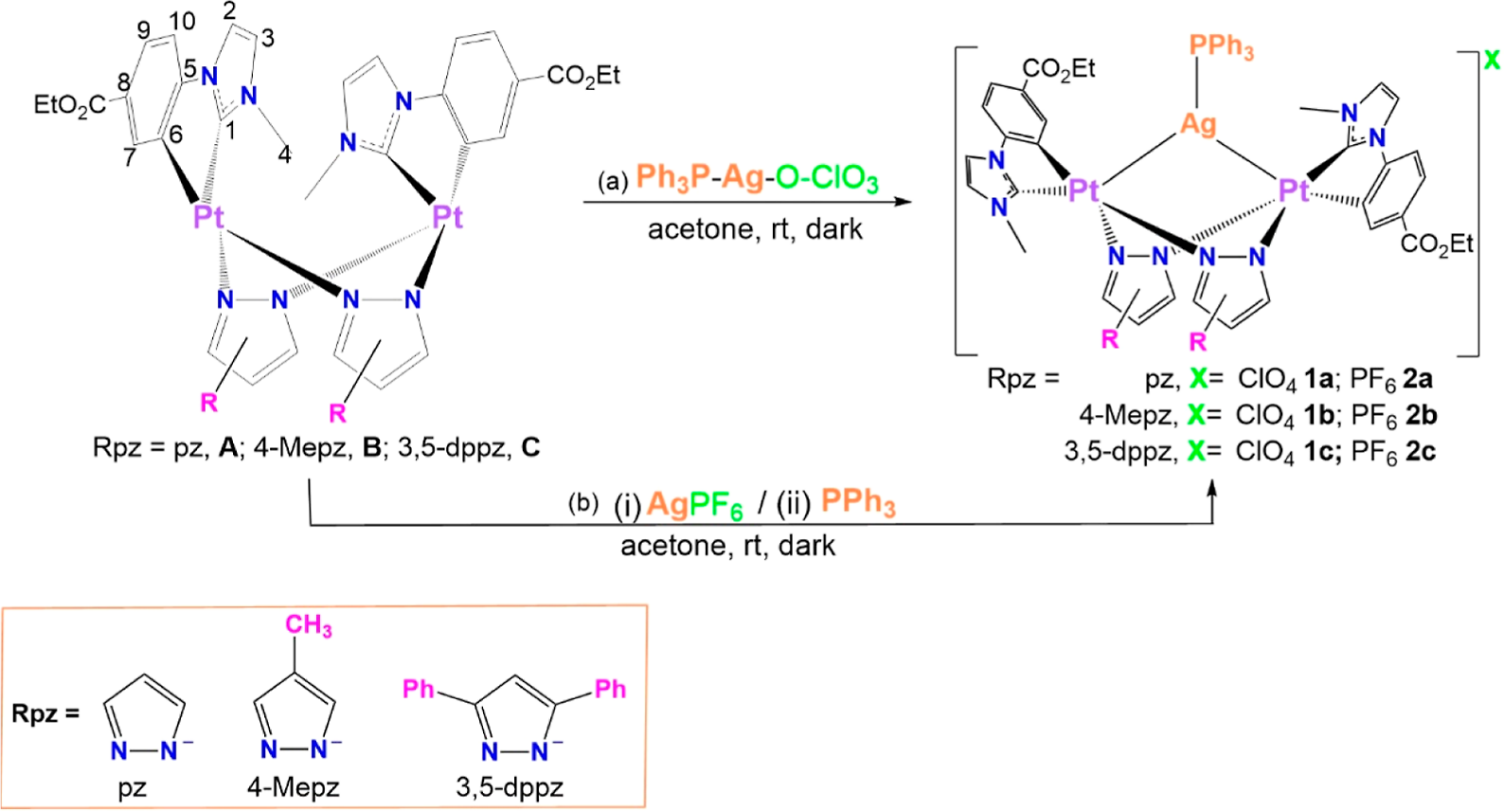
Reaction Pathways for the Synthesis of [Pt_2_AgPPh_3_]^+^ Clusters. Just the *anti* Isomers Have Been Represented for Clarity

The clusters [{Pt(EtO_2_C–C^∧^C*)(μ-Rpz)}_2_Ag(PPh_3_)]ClO_4_ (Rpz = pz **1a**, 4-Mepz **1b**, and 3,5-dppz **1c**) were prepared by the reaction of the Pt_2_ neutral complexes [{Pt(EtO_2_C–C^∧^C*)(μ-Rpz)}_2_] (Rpz = pz **A**, 4-Mepz **B**, and 3,5-dppz **C**) with an equimolar amount of [Ag(OClO_3_)(PPh_3_)] at r.t. in the dark (see Scheme 1, path a). All their spectroscopic data (MALDI (+) mass spectra, elemental analysis, and IR and multinuclear NMR spectra) are in agreement with the structures represented in Scheme 1, as can be seen in the experimental section and Figures S1–S3 in the Supporting Information and were then confirmed by single crystal X-ray diffraction studies (see below).

The NMR spectra of these [Pt_2_Ag(PPh_3_)]^+^ clusters provide evidence that they were obtained as a mixture of two isomers (*anti*/*syn*), as in the starting complexes, **A**–**C**. The isomer with the *anti-*orientation of the cyclometalated C^∧^C* groups is clearly the major one, while *syn* is, in some cases, almost imperceptible. The isomer *anti* (represented in Scheme 1), with a *C*_*2*_ symmetry, shows the signals corresponding to a fragment “Pt(EtO_2_C–C^∧^C*)(μ-Rpz)” along with those due to the PPh_3_ bonded to Ag. Some of the most relevant NMR data are compiled in Table 1. The ^195^Pt{^1^H} NMR spectra of compounds **1a**–**c** display just one signal, downfield shifted in more than 300 ppm with respect to that of the corresponding starting material (**A**–**C**), which is indicative of the electron density donation from Pt to Ag.

**Table 1:** Relevant NMR Data for **1a**–**c** and the Starting Substrates (**A**–**C**)^a^

comp	δ^195^Pt^b^	^1^***J***_Pt–Ag_	δ^31^P^c^	^1^***J***_P-109Ag_	^1^***J***_P-107Ag_	^2^***J***_P-195Pt_
**A**	–3796.0 (s)					
**1a**	–3490.4 (dd)	469	4.96	715.9	621.6	238.7
**B**	–3794.4(s)					
**1b**	–3487.7 (dd)	461	4.94	713.4	619.3	243.6
**C**	–3737.0 (s)					
**1c**	–3404.0 (dd)	465	4.18	668.9		206.7

a δ: ppm; *J*: Hz.

b 193 K.

c r.t.

The pattern consists of a doublet of doublets due to the ^195^Pt–Ag and ^195^Pt–P couplings. In no case could the individual ^195^Pt–^107^Ag and ^195^Pt–^109^Ag coupling constants be determined, even by recording the spectra at low temperatures.

**Figure 1:**
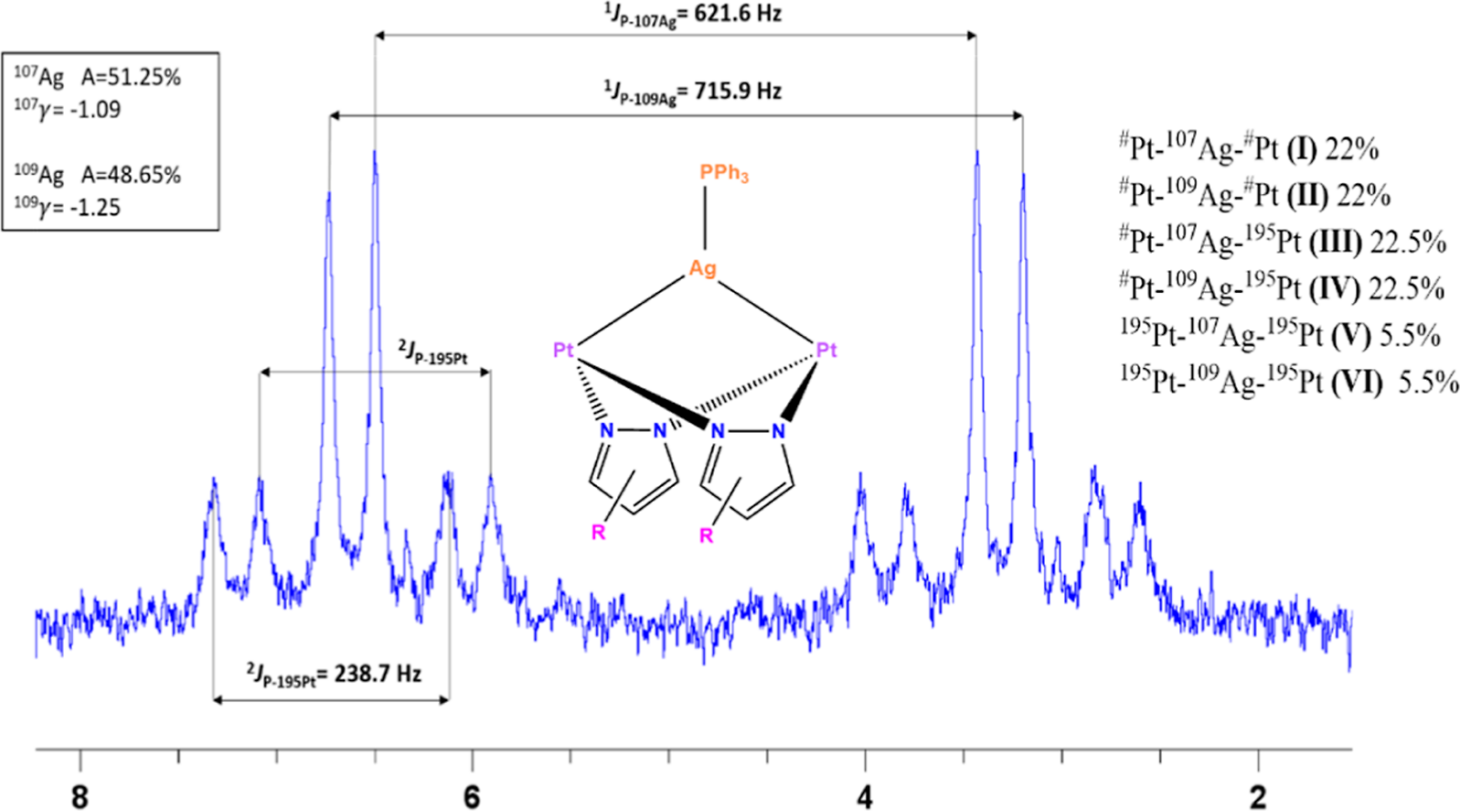
^31^P{^1^H} NMR spectrum of **1a** at RT in acetone-*d*_*6*_.

It is worth highlighting the ^31^P{^1^H} NMR spectra of compounds, [{Pt(EtO_2_C–C^∧^C*)(μ-Rpz)}_2_Ag(PPh_3_)]ClO_4_ (Rpz = pz **1a**, 4-Mepz **1b**, and 3,5-dppz **1c**) at room temperature for the relevant structural information they provide (see Figure 1 for **1a**). The ^31^P nucleus of the “Ag(PPh_3_)” fragment gives rise to a unique signal, whose pattern evidences the existence and robustness of the P–Ag and Ag–Pt bonds in solution. Its appearance is due to the existence of different isotopomers (I, II, III, IV, V, and VI in Figure 1). The most intense peaks correspond to species **I/II**, in which the two Pt centers are inactive in NMR. They appear as two doublets with similar intensity due to the similar natural abundance of the two stable isotopes, ^107^Ag and ^109^Ag, but with different values of ^31^P-^107^Ag and ^31^P-^109^Ag coupling constants. Species **III/IV** have one ^195^Pt atom (I = 1/2; 33% of Pt atoms), causing the appearance of ^195^Pt satellites. So, 45% of the signal corresponds to two AMX spin systems, giving two doublets of doublets. About 11% of the molecules (**V/VI**) containing two ^195^Pt atoms will exhibit an AMX_2_ spin system giving two doublets of triplets, but the low intensity of the corresponding peaks hinders them from being observed.

**Figure 2:**
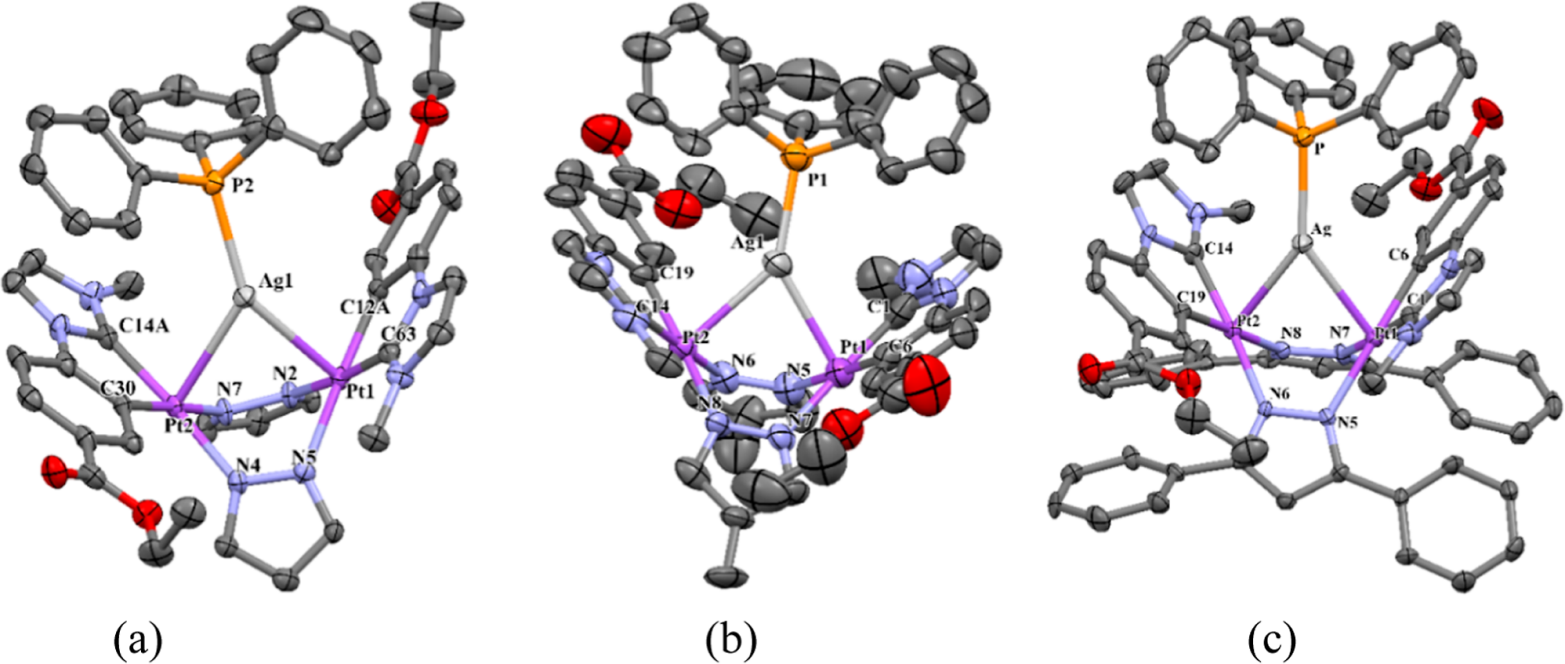
Molecular structures of the cations in compounds **1a** (a), **1b** (b), and **1c** (c). Thermal ellipsoids are drawn at the 50% probability level. Hydrogen atoms, solvent molecules, and ClO_4_^–^ have been omitted for clarity. [Pt(1)–Ag(1): 2.7844(5) Å **1a**, 2.7628(12) Å **1b**, 2.8012(7) Å **1c;** Pt(2)–Ag(1) 2.7396(5) Å **1a**, 2.7448(11) Å **1b**, and 2.7606(6) Å **1c**].

As seen in Figure 2, single-crystal X-ray diffraction of **1a**–**1c** confirmed the structures proposed for them on the basis of the multinuclear NMR data. In these trinuclear [Pt_2_Ag(PPh_3_)]^+^ complex units, the boat-like conformation of the six-membered ring Pt_2_N_4_ creates an appropriate site for the silver atom to be located.

The Pt–Ag bond distances (∼2.8 Å) and the near perpendicularity of the Pt–Ag vector to the corresponding Pt coordination planes [angles 9.70° (Pt1) and 10.76° (Pt2) in **1a**; 15.74° (Pt1) and 13.18° (Pt2) in **1b;** 19.34° (Pt1) and 17.35° (Pt2) in **1c**] are within the ranges found for Pt–Ag donor–acceptor bonds.^53−55^ The Ag center completes its coordination sphere with a PPh_3_ ligand, showing a highly distorted triangular coordination environment. A full description of these structures has been included in the Supporting Information (Tables S1 and S2). For further application, we prepared the same clusters but containing PF_6_^–^ as a counteranion, [{Pt(EtO_2_C–C^∧^C*)(μ-Rpz)}_2_Ag(PPh_3_)]PF_6_ (Rpz = pz **2a**, 4-Mepz **2b**, and 3,5-dppz **2c**). They were prepared in two steps (Scheme 1, path b). In the first step, (step (i) **A**–**C** were reacted with the equimolar amount of AgPF_6_ for 1 h. Then, a solution of PPh_3_ in acetone was added dropwise to the resulting solution of step i. Compounds **2a**–**2c** were also characterized by elemental analysis, IR, and NMR. Since all the spectroscopic data corresponding to the [Pt_2_Ag(PPh_3_)]^+^ clusters match those of **1a**–**1c**, just IR, ^31^P, and ^19^F NMR data corresponding to the anion PF_6_^–^ have been included in the experimental section along with the corresponding spectra (Figures S4–S6).

### UV–vis Spectra of 1a–1c and Theoretical Calculations

**Figure 3:**
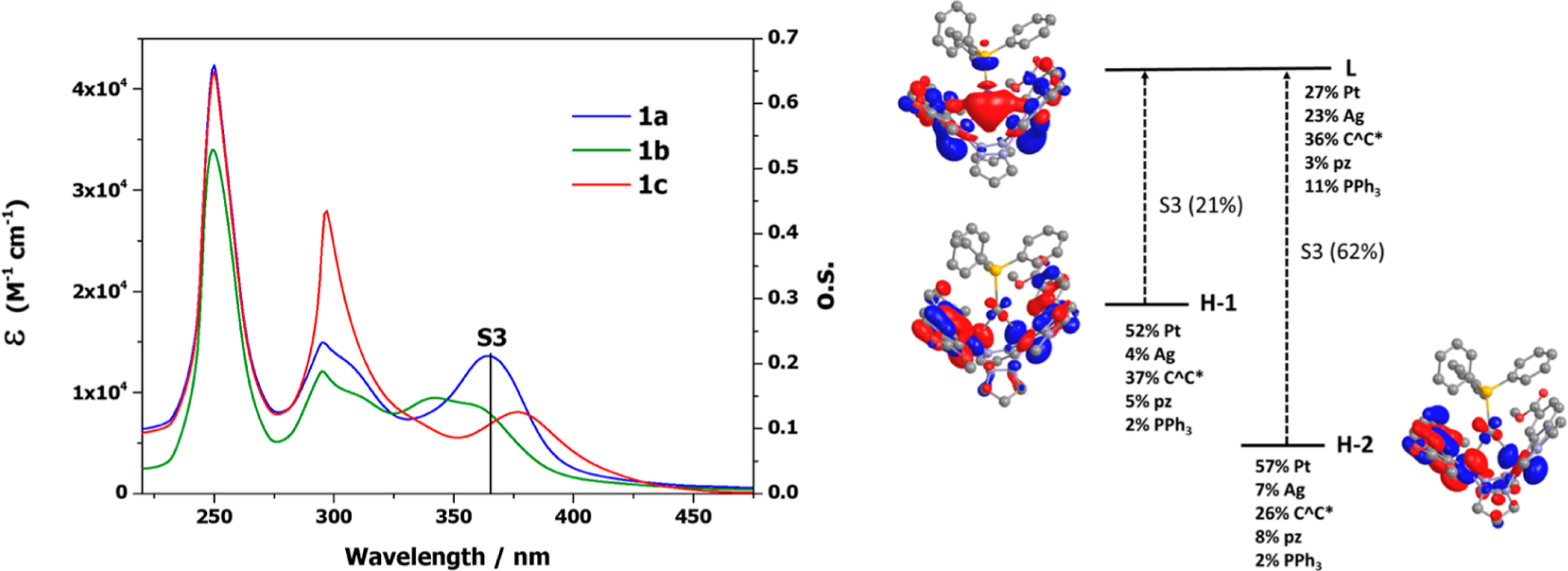
UV–visible spectra in 2-MeTHF 10^–4^ M of **1a**–**1c**. The most significant transition was calculated in THF for **1a** (S_3_ in bars) and plots (isovalue 0.03) of the molecular orbitals involved in S_3_.

The UV–vis spectra of **1a**–**1c** were recorded in 10^–4^ M solutions of 2-MeTHF and have been plotted in Figure 3. All data appear listed in Table S3 and plotted in Figure S7 in the Supporting Information, together with those for the starting complexes, **A**–**C**, for comparison.^52^ In general, the lowest-energy absorption bands of the [Pt_2_Ag(PPh_3_)]^+^ clusters appear in the range 350–425 nm, red-shifted with respect to that of the corresponding Pt_2_ complex, **A**–**C**, indicating that the existence of the Pt–Ag bonds has a clear effect on the photophysical properties of these clusters.

To provide further insight on the lowest energy absorption’s nature, DFT and TD-DFT studies were performed on complex [{Pt(C^∧^C*)(μ-pz)}_2_]Ag(PPh_3_)]^+^ (**1a**) (see Tables S4 and S5 and Figure S8 in the Supporting Information). The main calculated spin-allowed transition, S_3_, matches with the registered lowest-energy absorption (see Figure 3 for **1a**). Analysis of the FOs in the ground state involved in this transition showed that the HOMO, H-1 and H-2, are mainly constructed from orbitals of Pt (43% HOMO, 52% H-1, 57% H-2) and C^∧^C* (43% HOMO, 37% H-1, and 26% H-2). The LUMO has a contribution of Pt (27%) and C^∧^C* (36%) orbitals, along with an important contribution of Ag and PPh_3_ (23% and 11%, respectively). However, the pz groups have a residual contribution to the FOs (3–9%). Considering that the calculated S_3_ state arises mainly from H-2→ LUMO (62%) and H-1→ LUMO (21%) transitions, the lowest-energy absorption can be attributed to transitions with a mixed nature, ^3^MM′CT/MLCT [5d(Pt) → 5sp^n^ (Ag)π(PPh_3_)/5d(Pt) → π*(C^∧^C*)].

The similarities between the clusters would allow us to assume the same theoretical explanation for all of them. In clusters **1a**–**1b**, the presence of the [Ag(PPh)_3_]^+^ unit changes the nature of the lowest energy absorption and shifts it to red with respect to the starting materials **A** and **B** (IL/MLCT). In **1c**, because the presence of [Ag(PPh)_3_]^+^ prevents the interaction of the d_*z*_2 orbitals of both platinum centers, it cancels the contribution of the ^1^MMLCT [dσ*(Pt–Pt) → π*(C^∧^C*)] transition operating in the case of **C**. Therefore, both effects would be compensated, and the lowest energy absorption band in **1c** almost matches that in **C**.

The UV–vis absorption spectra at rt of thin films of the PF_6_^–^ derivatives, **2a**–**2c**, are quite similar to those of the ClO_4_^–^ ones in 2-MeTHF solution. They show, as well, their lowest energy absorption bands in the range of 350–425 nm, with maxima at *ca*. 370 nm (see Figure S9 in Supporting Information).

### Photo- and Electroluminescence

**Figure 4:**
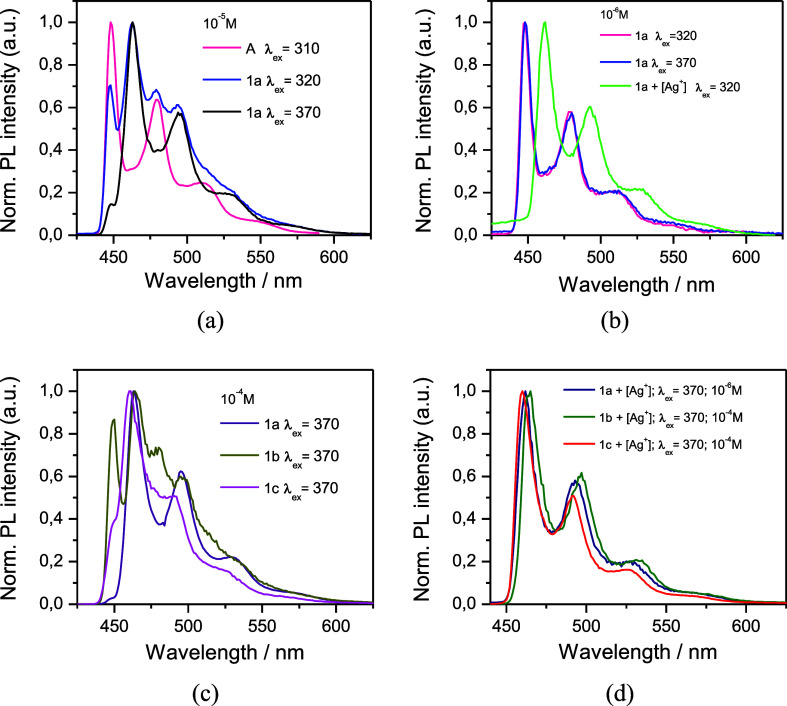
Normalized emission spectra in glassy 2-MeTHF (77 K) of **A** and **1a** (10^–5^ M, **a**); **1a**, 10^–6^ M with and without [Ag^+^] **b**; **1a, 1b, 1c** (10^–4^ M, λ_exc_ = 370 nm, **c**); and **1a, 1b, 1c** with excess [Ag^+^] **d**). [Ag^+^] = [Ag(OClO_3_)(PPh_3_)].

**Figure 5:**
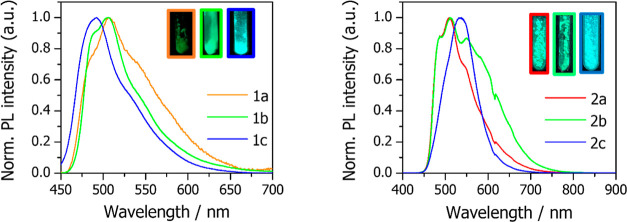
**Left:** Normalized emission spectra of powdery samples of **1a**–**1c** at r.t. **Right:** Normalized emission spectra of neat thin films of **2a**–**2c** at r.t. Insets: Pictures of powdery samples of **1a**–**1c** and **2a**–**2c**, taken under 365 nm UV light at room temperature in the air.

Compounds **1a**–**1c** do not display luminescence in 2-MeTHF solution under UV light at rt, not even in an argon atmosphere, like other discrete-emitting Pt(II) complexes bearing a cyclometalated NHC ligand (C^∧^C*). This behavior is very often attributed to thermal quenching processes via the population of higher-lying dd* states or the formation of exciplexes.^50^ However, in rigid media, like glassy 2-MeTHF (77 K) or solid state, **1a**–**1c** show bright phosphorescence (Table 2 and Figures 4 and 5).

**Table 2:** Emission Data for Complexes **1a**–**c**

	media	λ_ex_(nm)	λ _em_(nm)	τ (μs)	Φ^d^(%)
**1a**	2-MeTHF^a^	320	465_max_, 497, 535 tail to 600	5.87	
	solid	440	484, 506_max_, 535	0.17 (16%), 0.92 (84%)	3
**A**	2-MeTHF^b^	310	448_max_, 479, 510 tail to 575	0.4 (20%), 1.4 (80%)	3
	solid	390	469, 527_sh_, 556_max_		
**1b**	2-MeTHF^c^	370	465_max_, 497, 531 tail to 600	5.83	
	solid	440	490, 506_max_, 540_sh_	0.23 (22%), 0.82 (78%)	6
**B**	2-MeTHF^b^	340	449_max_, 480, 510 tail to 575	0.3 (22%), 1.1 (78%)	3
	solid	390	472, 527_sh_, 559_max_		
**1c**	2-MeTHF^c^	370	460_max_, 491, 525 tail to 600	6.59	
	solid	450	492_max_	0.49(29%), 1.23(71%)	20
**C**	2-MeTHF^b^	335	449_max_, 476, 510 tail to 575	0.5 (30%), 1.1 (70%)	29
	solid	390	469,482_max_, 553_sh_		

a 10^–6^ M + [Ag(OClO_3_)(PPh_3_)], 2-MeTHF, Ar, 77K.

b 10^–5^ M, Ar, 77K.

c 10^–4^ M + [Ag(OClO_3_)(PPh_3_)], 2-MeTHF, Ar, 77K.

d Air, 298 K.

In glassy 2-MeTHF (10^–5^ M, 77 K), the emission of **1a** resulted to be excitation-wavelength dependent (see Figure 4a). For instance, upon excitation in the low-lying absorption region (λ: 350–400 nm), **1a** shows a bright phosphorescence with maxima in the sky-blue spectral range (λ_max_ = 463 nm). The structured shape of the emission band, with vibrational spacings [1300–1450 cm^–1^] corresponding to the C=C/C=N stretches of the C^∧^C* ligand, suggests the involvement of this in their excited state. Upon excitation at λ ≤ 340 nm, it shows an additional structured band at λ_max_ = 448 nm, which matches with that observed for the starting complex [{Pt(EtO_2_C–C^∧^C*)(μ-pz)}_2_](**A**) under the same conditions.

To explain the presence of the high energy emission, we tested first the photostability of the clusters, which was confirmed as the ^1^H and ^31^P{^1^H} NMR spectra of **1a** in THF-*d*_*8*_ before and after irradiation with λ = 365 nm for 15 min match one to another (see Figure S10), indicating that irradiation does not cause the cluster to break up. Then, we evaluated whether the following dissociation equilibrium (eq 1) might occur in solution.
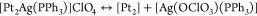
1

For that, we measured the luminescence of **1a** in 2-MeTHF at different concentrations. At 10^–4^ M the emission is still excitation-wavelength dependent, but upon excitation at λ_exc_ = 370 nm, the low energy band (λ_max_ = 463 nm) is the only one observed (Figure S11a). By contrast, at 10^–6^ M the emission band of high energy (λ_max_ = 448 nm), matching the emission band of the starting [Pt_2_] complex **A**, is the only one observed, regardless of the excitation wavelength (Figure 4b). The addition of [Ag(OClO_3_)(PPh_3_)] to the 10^–6^ M solution rendered just the low energy emission (λ_max_ = 462 nm), attributable to the cluster [Pt_2_Ag(PPh_3_)]^+^**1a**, and proving that the addition of [Ag(OClO_3_)(PPh_3_)] shifts the equilibrium to the left.

Therefore, these results are consistent with the dissociation equilibrium represented in eq 1, operating in solution, being more important as the concentration decreases.

The clusters **1b** and **1c** were found to exhibit the same behavior in 2-MeTHF at 77K than **1a** (see Figures S11b,c). Their solutions 10^–4^ and 10^–5^ M exhibit two emission bands with that corresponding to the cluster (λ_max_ ∼ 463 nm) becoming more intense as the concentration is higher (10^–4^ M vs 10^–5^ M), and the excitation wavelength is longer (λ_exc_ = 370 nm). In view of this, it would have been desirable to record the luminescence spectra of these complexes at concentrations higher than 10^–4^ M, but their scarce solubility prevents this. By comparing the emission spectra of **1a**–**1c** in the same conditions (10^–4^ M, 2-MeTHF, 77K, λ_exc_ = 370 nm Figure 4c), it seems that the dissociation follows the trend **1a** < **1c** < **1b**. In all cases, the addition of [Ag(OClO_3_)(PPh_3_)] shifts the equilibrium to the left, resulting just in the emission corresponding to the clusters (Figure 4d). These data have been summarized in Table 2, and they show that the incorporation of the fragment “Ag(PPh_3_)^+^” clearly shifts the emission to the red compared to those of the [Pt_2_] precursors (see Table 2).

TD-DFT calculations carried out on **1a** revealed the existence of two close low-lying triplets, T_1_ (λ = 452.64 nm) and T_2_ (λ = 449.75 nm), calculated at the ground state that match very well with the experimental λ_max_ (Figure S8 and Tables S4 and S5 in the Supporting Information).

Considering Kasha’s rule,^56^ the emission should originate from the lowest excited state, that is, from T_1_ which shows a mixed character ^3^MM′CT/MLCT [5d(Pt) → 5sp^n^ (Ag)π(PPh_3_)/5d(Pt) → π*(C^∧^C*)], confirming the involvement of Ag orbitals in the emitting state.

Powdery samples of **1a**–**1c** show at r.t. in the air emission bands (see Figure 5 left) that resemble those corresponding to the clusters in glassy 2-MeTHF, with moderate-low PLQYs values, of 3, 6 and 20% for **1a**, **1b**, and **1c**, respectively (Table 2).

The emission properties of the PF_6_^–^ derivatives (**2a**–**2c**) in the neat film were also determined (Figure 5 right). For that, 100 μL of 20 mg/mL complex solutions in dichloromethane (DCM) were spin-coated on quartz substrates and irradiated with a 365 nm-UV light. Interestingly, while the λ_max_ values of the photoluminescence (PL) for **2a** and **2b** (509 and 511 nm, respectively) are comparable with those found for the ClO_4_^–^ counterparts (λ_max_ = 506 nm for both **1a** and **1b**), the emission of **2c** happens to be red-shifted upon anion exchange (λ_max_ = 535 nm versus 492 nm for **1c**).

With the intention of utilizing **2a**–**2c** for the fabrication of light-emitting devices, the PLQY of such thin films was also determined. Under a nitrogen atmosphere, PLQY values of 5.0, 6.6, and 39%, were obtained for **2a**, **2b** and **2c**, respectively. The three clusters show the same trend of PLQY as that of the ClO_4_^–^ counterparts, with the phenyl substituents on the pyrazolate bridges having a beneficial effect on PLQY, especially in the case of **2c**. Moreover, these results indicate that the presence of [Ag(PPh_3_)] ^+^ in the clusters allows to get iTMCs with tunable phosphorescence with respect to the Pt_2_ precursors.

In view of these results, we focused on **2c** and its neutral precursor, complex **C**, as active materials on light-emitting devices. First, we studied the electroluminescence (EL) properties of **C** as a reference. OLEDs were fabricated using the following stack configuration: ITO/PEDOT/PSS (40 nm)/TAPC (10 nm)/mCP (10 nm)/emitter **C** (either 1 or 10 nm)/PO-T2T (60 nm)/Ba (5 nm)/Ag (70 nm). In them, ITO is indium tin oxide, PEDOT/PSS is poly(3,4-ethylenedioxythiophene):poly(styrenesulfonate), TAPC is 1,1-bis[(di-4-tolylamino)phenyl]cyclohexane, mCP is 1,3-bis(N-carbazolyl)benzene, and PO-T2T is 2,4,6-tris[3-(diphenylphosphinyl)phenyl]-1,3,5-triazine). Details of the device fabrication are described in Supporting Information. In short, PEDOT: PSS was spin-coated on top of an ITO-coated glass substrate, to enlarge the work function and flatten the ITO electrode. Then, the substrates were taken into a vacuum chamber, where TAPC, mCP, **C**, PO-T2T, Ba, and Ag were sequentially deposited. The emitting layer consisted of either a thick (10 nm) or an ultrathin (1 nm) layer of undoped complex **C**.

**Figure 6:**
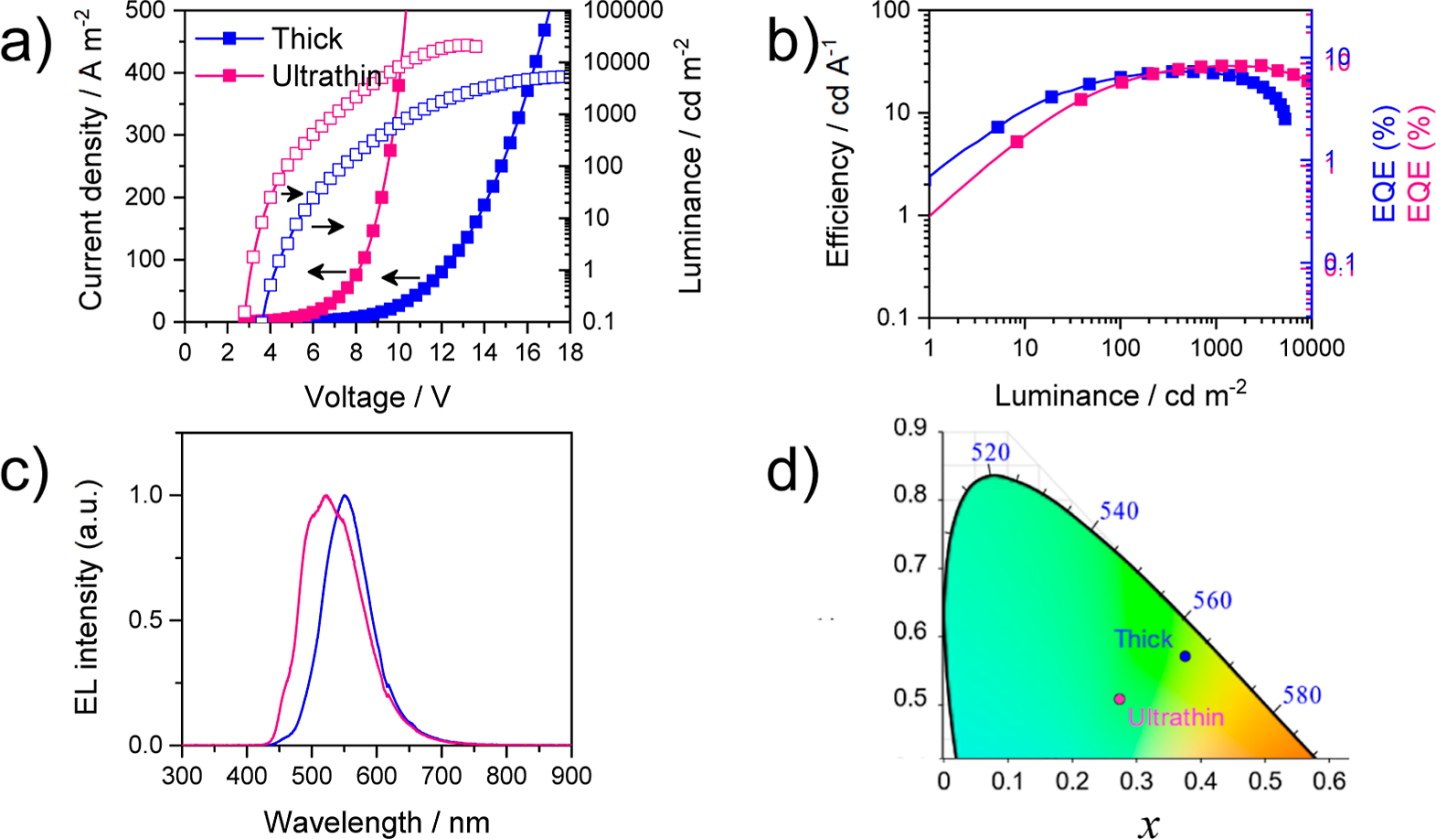
(a) Current density and luminance versus voltage characteristics for OLEDs employing **C** (thick: 10 nm, ultrathin: 1 nm layer) as the emitter. (b) Efficiency of the devices versus luminance. (c) Electroluminescence spectra collected from the devices. (d) CIE 1931 chromaticity diagram showing the two corresponding color points (cropped).

Figure 6a–b exhibits the current density and luminance versus voltage (*JVL*) and the efficiency versus luminance curves, where devices with two different thicknesses of the emitting layer clearly showed different behavior. In the case of devices with a thick **C** layer, the turn-on voltage was 4.4 V (1 cd m^–2^). The maximum measured luminance was 7502 cd m^–2^ at 18 V, with a maximum efficiency recorded of 25.2 cd A^–1^ (corresponding to an EQE of 7.1%) at a luminance of 513.8 cd m^–2^ (9.60 V bias voltage). On the other hand, devices with an ultrathin emitting layer exhibited better performance. The turn-on voltage was lower, 3.2 V (1 cd m^–2^), and the luminance reached a peak value of 21,357 cd m^–2^ at 13 V. Moreover, they did not only have a higher peak current efficiency (CE) (28.8 cd A^–1^, corresponding to 9.5% EQE, at a luminance of 2975 cd m^–2^), but they also reached it at a lower bias (8.40 V), which accounts for an even higher power efficiency (PE). They also suffered less from efficiency roll-off (67% of the peak value at 10,000 cd m^–2^). The electroluminescence spectra of the two types of devices are shown in Figure 6c. The EL spectrum of the device with the thick layer is nearly coincident with the PL spectrum of the solid after mechanical grinding, with the emission due to a ^3^MMLCT state of molecules with a butterfly-folded configuration and short Pt···Pt distances.^52^ The devices with the ultrathin emitting layer have a broader and slightly blue-shifted spectrum, with a higher contribution from the ^3^IL/MLCT state of butterfly spread molecules exhibiting long Pt···Pt distances. These changes in the structural conformation seem to be induced by the thickness of the layer and are reflected on the International Commission on Illumination (CIE) color coordinates of the EL: (0.38, 0.57) and (0.28, 0.51) for the thick and ultrathin, respectively (Figure 6d). These results indicate that these green OLEDs exhibit better performance than those fabricated from [{Pt(C^∧^N)(μ-Rpz)}_2_] (C^∧^N = 2-(4′,6′-difluorophenyl)pyridinato-N,C^2’^; Rpz = 3-methyl-5-*tert*-butylpyrazolate), where a maximum EQE of 6.0% at similar luminance values (2517 cd m^–2^) was reported.^33^

EL under different biases was measured for both devices, thin, and thick emitting layers. Only a small blue-shift with increasing voltage (λ_max_ of 553 nm at 10 V and 550 nm at 16 V) was observed for the 10 nm thick devices and a small variation in the relative intensity of the emission peak’s features for the 1 nm thick devices, which is also reflected on the CIE coordinates (Figure S12). The lifetimes of devices with an ultrathin emitting layer were also measured. Due to the frequently observed antagonistic relationships between triplet–triplet annihilation extent and OLED lifetime,^57^ devices with an ultrathin emitting layer are expected to have a longer lifetime. Moreover, they can reach the same luminance value at lower current densities, which is beneficial toward slowing down their degradation process.^58^ In our case, devices were operated under a constant current with a current density of 8 A m^–2^, which resulted in an initial luminance of 100 cd m^–2^ and a long half-luminance lifetime t_50_ (time to reach half of the initial luminance) of 15.7 h (Figure S13).

Given these promising results, OLEDs were fabricated using complex **2c** as the emitter. Taking into account the ionic nature of **2c**, devices were solution-processed with the stack configuration: ITO/PEDOT/PSS (40 nm)/CBP:**2c** (6 wt %, 30 nm)/BmPyPhB (30 nm)/Ba (5 nm)/Ag (70 nm) (where CBP is 4,4′-bis(carbazol-9-yl)biphenyl and BmPyPhB is 1,3-bis[3,5-di(pyridin-3-yl)phenyl]benzene). Details of the device fabrication are described in the Supporting Information. The emitting layer was spin-coated from solutions of the CBP host containing 6 wt % of complex **2c**. The devices were finished by vacuum deposition of a 30 nm-thick BmPyPhB electron transport layer and a Ba/Ag cathode.

**Figure 7:**
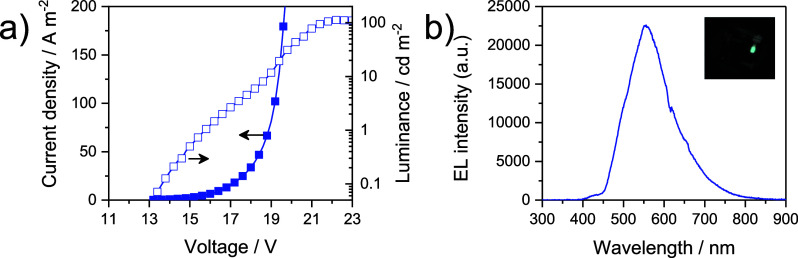
(a) Current density and luminance versus voltage characteristics for OLEDs employing **2c** as the emitter. (b) Electroluminescence spectra collected from the same device; the inset shows a picture of a working pixel.

Figure 7a shows the *JVL* curves registered for the two OLEDs. Electroluminescence was only detected at high bias (13 V) with a maximum luminance of 114 cd m^–2^ at 22.4 V. The electroluminescence spectrum, shown in Figure 7b, falls within the yellow-green region of the electromagnetic spectrum (CIE coordinates: 0.40, 0.51) and is independent of the applied voltage (Figure S14). The overall CE is rather moderate (0.2 cd A^–1^, corresponding to 0.08% EQE, which further decreases to 0.08 cd A^–1^ at the maximum luminance).

**Figure 8:**
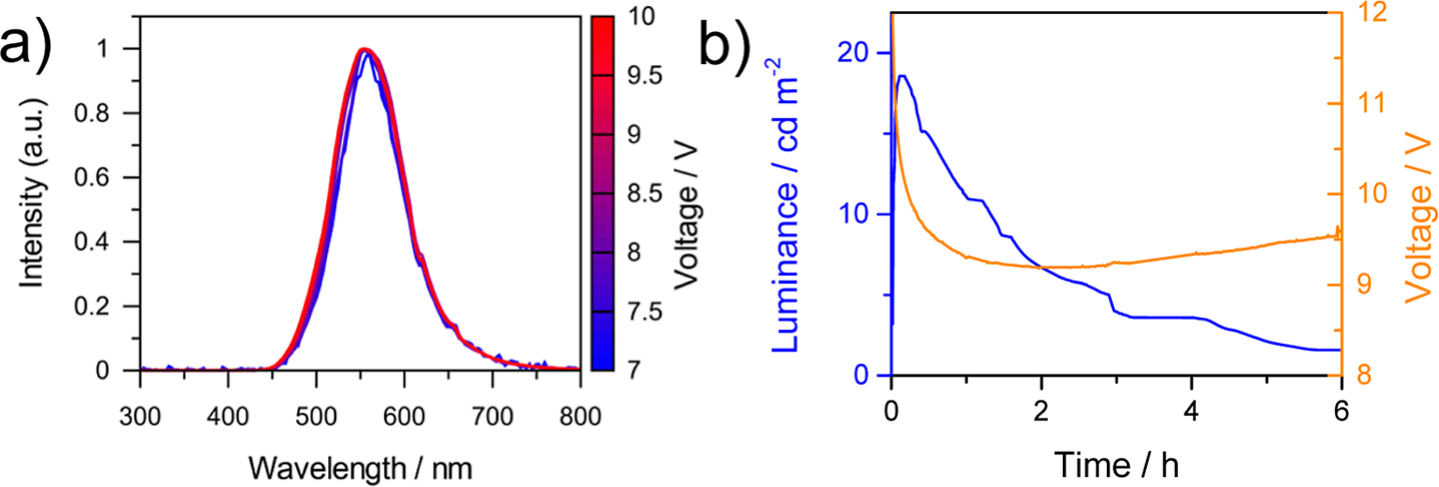
(a) Electroluminescence spectra of LECs employing **2c** as the emitting material driven at different voltage values, from 7 to 10 V. (b) Time-dependence of the luminance and the voltage of LECs driven by a pulsed current with an average current density of 50 A m^–2^.

The need for a high bias to achieve significant current through the device (14.2 V for 1 A/m^2^) not only limits the maximum PE (0.04 lm/W) but also is an indication of a problematic charge injection. In an attempt to enhance charge injection and increase device efficiency, LECs using **2c** as the emitter were fabricated. LECs differ from the OLED by the presence and action of mobile ions in the active layer: after applying an external bias, anions and cations migrate according to the electrical field to form electric double layers at the interfaces, which in turn enhances carrier injection.^59^ In the devices fabricated in this study, the ionic complex, which performs the dual role of emitter and charge transporter, was mixed with the ionic liquid (IL) 1-butyl-3-methyl-imidazolium-hexafluorophosphate (BMIM^+^PF_6_^–^) in a molar ratio of 3.14 to 1 (iTMC/IL). The IL provided additional ions in the light-emitting layer. The two components were dissolved in DCM to form a solution with a final **2c** concentration of 20 mg/mL. From such a solution, devices were fabricated adopting the archetypical stack configuration ITO/PEDOT/PSS (80 nm)/**2c**/IL (90–120 nm)/Al (100 nm). Devices were first characterized by recording their electroluminescence (EL) spectra at different voltages. Lower voltages (when compared with the OLED using the same active material) were required to record the EL spectra showing an EL that is voltage-independent with a peak centered at 555 nm in the yellow-green emission (Figure 8a) with CIE coordinates of 0.40 and 0.55 (Figure S15). Then, LEC devices were tested by measuring their performance over time. In particular, they were operated under pulsed current driving with an average current density of 50 A m^–2^ (1000 Hz block-wave, 50% duty cycle with a peak current of 100 A m^–2^). The resulting time-dependence of the luminance and voltage is shown in Figure 8b. Devices showed maximum luminance values of about 20 cd m^–2^, CE values of around 0.2 cd A^–1^ and PE values of around 0.06 lm W^1–^, with a half-luminance lifetime (t_50_) value of 50 min. These results are comparable and even superior to those of other Pt-based LECs using polymetallic iTMC as emitters [{4,4′-*t*Bu_2_(C^∧^N^∧^N)}_3_Pt_3_(μ_3_-dpmp)]^3+^ (HC^∧^N^∧^N = 6-aryl-2,2′-bipyridine; dpmp = bis(diphenylphosphinomethyl)phenylphosphine)^45^ with maximum luminance levels of 10 cd m^–2^ and [((N^∧^C^∧^N)Pt)_2_(μ-pz)]^+^ (N^∧^C^∧^N = 1,3-di(2-pyridyl)benzene; μ-pz = 3,5-diphenylpyrazolate)^48^ with 20 cd m^–2^.

When compared with our previously reported carbazole-appended NHC ligand cyclometalated platinum(II) complexes,^44^ a lower performance is observed, probably due to the rather deep level of the HOMO in the cluster (−6.70 eV, Table S4) that hinders effective electron removal. The great stabilization of the HOMO compared to that of the precursor (−5.80 eV),^52^ can be attributed to the acidic character of fragment [Ag(PPh_3_)]^+^. Despite that, the results obtained from **2c** are comparable, or even better than those reached in OLEDs based on [Pt(CN–C^∧^C*)(dppm)]PF_6_,^50^ [Pt(CN–C^∧^C*)(PPh_3_)py]PF_6_,^51^ and [Pt(CO_2_Et–C^∧^C*)(dppm)]PF_6_^49^ containing electron withdrawing groups on the phenyl ring of the C^∧^C* ligand.

## Conclusions

The neutral complexes [{Pt(C^∧^C*)(μ-Rpz)}_2_] (HC^∧^C* = 1-(4-(ethoxycarbonyl)phenyl)-3-methyl-1*H*-imidazole-2-ylidene, Rpz = pz **A**, 4-Mepz **B**, and 3,5-dppz **C**) contain two platinum(II) fragments in an “open book” disposition with the metal centers basic enough to form Pt–Ag donor–acceptor bonds. These facts enable the formation of the ionic transition metal complexes [{Pt(C^∧^C*)(μ-Rpz)}_2_Ag(PPh_3_)]^+^ (Rpz = pz, 4-Mepz, and 3,5-dppz), which could be isolated as ClO_4_^–^ (**1a**–**1c**) and PF_6_^–^ (**2a**–**2c**) salts.

Experimentally, it was found that the presence of “Ag(PPh_3_)^+^” shifts the lower-energy absorption and the emission of the clusters, **1a**–**1c**, to the red with respect to those of their corresponding precursor, **A**–**C**. This is consistent with the involvement of the Ag orbitals in the LUMOs of the clusters, in such a way that the lower energy absorption and emission have a mixed nature, ^1/3^MM′CT/MLCT [5d(Pt) → 5sp^n^ (Ag)π(PPh_3_)/5d(Pt) → π*(C^∧^C*)], instead of the ^1/3^IL/MLCT nature in the precursors.

The Ph substituents on the pyrazolate bridges increase the PLQYs with respect to H and Me, and both **2c** and its precursor, **C**, were used as active materials on light-emitting devices. OLEDs based on **C** were fabricated with two different layer thicknesses (10 and 1 nm). Devices showed an emission dependent on the emitter thickness, most probably due to different emissive states related to the Pt···Pt distances. The ultrathin (1 nm) OLEDs showed the best performance with a turn-on voltage of 3.2 V, a luminance peak of 21,357 cd m^–2^ at 13 V, and a peak CE of 28.8 cd A^–1^ (9.5% EQE). Using **2c** as an emitter, solution-processed OLEDs and LECs showed voltage-independent electroluminescence. **2c** OLEDs showed a maximum luminance of 114 cd m^–2^, while LECs exhibited a maximum luminance of 20 cd m^–2^ and a CE of around 0.2 cd A^–1^, with a half-luminance lifetime (t_50_) value of 50 min. The incorporation of a cationic “Ag(PPh_3_)” fragment into a neutral phosphorescent Pt complex was revealed to be a suitable strategy to obtain iTMCs for LECs with competitive performances within this novel group of heteropolymetallic emitters.

## Supporting Information Available

Experimental Section (general procedures for synthesis, characterization, computational methods, and X-ray determination (CCDC nos. 2309669–2309671 for 1a, 1b, and 1c), device fabrication, and synthesis and characterization data of the new compounds); spectra for characterization, full description of the X-ray structures, data and figures for absorption theoretical calculations, and emission properties and electroluminescence; and Cartesian coordinates of the DFT-optimized structures for complex 1a (X,Y,Z) (PDF)

## References

[ref1] Tang C. W.; VanSlyke S. A. Organic Electroluminescent Diodes. Appl. Phys. Lett. 1987, 51, 913–915. 10.1063/1.98799.

[ref2] Hong G.; Gan X.; Leonhardt C.; Zhang Z.; Seibert J.; Busch J. M.; Brase S. A Brief History of OLEDs—Emitter Development and Industry Milestones. Adv. Mater. 2021, 33, 2005630 10.1002/adma.202005630. 33458866

[ref3] Che C. M.; Kwok C. C.; Kui C. F.; Low K. H. Luminescent Coordination and Organometallic Complexes for OLEDs. In Comprehensive Inorganic Chemistry II: from elements to applications, 2nd ed.; Reedijk J.; Poeppelmeier K., Eds.; Elsevier: Amsterdam, 2013; pp 607–654.

[ref4] Ma Y.; Zhang H.; Shen J.; Che C. Electroluminescence From Triplet Metal—Ligand Charge-Transfer Excited State of Transition Metal Complexes. Synth. Met. 1998, 94, 245–248. 10.1016/S0379-6779(97)04166-0.

[ref5] Baldo M. A.; O’Brien D. F.; You Y.; Shoustikov A.; Sibley S.; Thompson M. E.; Forrest S. R. Highly Efficient Phosphorescent Emission from Organic Electroluminescent Devices. Nature 1998, 395, 151–154. 10.1038/25954.

[ref6] Wang X.; Wang S. Phosphorescent Pt(II) Emitters for OLEDs: From Triarylboron-Functionalized Bidentate Complexes to Compounds with Macrocyclic Chelating Ligands. Chem. Rec. 2019, 19, 1693–1709. 10.1002/tcr.201800165. 30648808

[ref7] Haque A.; Xu L.; Al-Balushi R. A.; Al-Suti M. K.; Ilmi R.; Guo Z.; Khan M. S.; Wong W. Y.; Raithby P. R. Cyclometallated Tridentate Platinum(II) Arylacetylide Complexes: Old Wine in New Bottles. Chem. Soc. Rev. 2019, 48, 5547–5563. 10.1039/c8cs00620b. 31720563

[ref8] Pashaei B.; Karimi S.; Shahroosvand H.; Abbasi P.; Pilkington M.; Bartolotta A.; Fresta E.; Fernandez-Cestau J.; Costa R. D.; Bonaccorso F. Polypyridyl ligands as a Versatile Platform for Solid-State Light-Emitting Devices. Chem. Soc. Rev. 2019, 48, 5033–5139. 10.1039/C8CS00075A. 31418444

[ref9] Ibrahim-Ouali M.; Dumur F. Recent Advances on Metal-Based Near Infrared and Infrared Emitting OLEDs. Molecules 2019, 24, 1412 10.3390/molecules24071412. 30974838 PMC6480698

[ref10] Cebrian C.; Mauro M. Recent Advances in Phosphorescent Platinum Complexes for Organic Light-Emitting Diodes. Beilstein J. Org. Chem. 2018, 14, 1459–1481. 10.3762/bjoc.14.124. 30013674 PMC6037003

[ref11] Elie M.; Renaud J. L.; Gaillard S. N-Heterocyclic Carbene Transition Metal Complexes in Light Emitting Devices. Polyhedron 2018, 140, 158–168. 10.1016/j.poly.2017.11.045.

[ref12] Fleetham T.; Li G. F.; Li J. Phosphorescent Pt(II) and Pd(II) Complexes for Efficient, High-Color-Quality and Stable OLEDs. Adv. Mater. 2017, 29, 1601861 10.1002/adma.201601861. 27859829

[ref13] Murphy L.; Williams J. A. G. Luminescent Platinum Compounds: From Molecules to OLEDs. Top Organomet. Chem. 2010, 28, 75–111. 10.1007/978-3-642-01866-4_3.

[ref14] Chaaban M.; Zhou C.; Lin H.; Chyi B.; Ma B. Platinum(II) Binuclear Complexes: Molecular Structures, Photophysical Properties and Applications. J. Mater. Chem. C 2019, 7, 5910–5924. 10.1039/C9TC01585J.

[ref15] Sun Y.; Liu B.; Guo Y.; Chen X.; Lee Y.-T.; Feng Z.; Adachi C.; Zhou G.; Chen Z.; Yang X. Developing Efficient Dinuclear Pt(II) Complexes Based on the Triphenylamine Core for High-Efficiency Solution-Processed OLEDs. ACS Appl. Mater. Interfaces 2021, 13, 36020–36032. 10.1021/acsami.1c06148. 34283914

[ref16] Zhang Q.-C.; Xiao H.; Zhang X.; Xu L.-J.; Chen Z.-N. Luminescent Oligonuclear Metal Complexes and the Use in Organic light-emitting Diodes. Coord. Chem. Rev. 2019, 378, 121–133. 10.1016/j.ccr.2018.01.017.

[ref17] Tronnier A.; Strassner T. (C^C*) Cyclometalated Binuclear N-heterocyclic Biscarbene Platinum(II) Complexes – Highly Emissive Phosphorescent Emitters. Dalton Trans. 2013, 42, 9847–9851. 10.1039/c3dt50841b. 23689316

[ref18] Luo J.; Liu Y.; Chen Q.; Shi D.; Huang Y.; Yu J.; Wang Y.; Zhang Z.; Lei G.; Zhu W. Synthesis, Optoelectronic Properties of a Dinuclear Platinum(II) Complex Containing a Binary Cyclometalated Ligand in the Single-Emissive-Layer PLEDs. Dalton Trans. 2013, 42, 1231–1237. 10.1039/C2DT31863F. 23138411

[ref19] Zhang Y.-M.; Meng F.; Tang J.-H.; Wang Y.; You C.; Tan H.; Liu Y.; Zhong Y.-W.; Su S.; Zhu W. Achieving Near-Infrared Emission in Platinum(II) Complexes by Using an Extended Donor-Acceptor-Type Ligand. Dalton Trans. 2016, 45, 5071–5080. 10.1039/C5DT04793E. 26880278

[ref20] Yang X.; Jiao B.; Dang J.-S.; Sun Y.; Wu Y.; Zhou G.; Wong W.-Y. Achieving High-Performance Solution-Processed Orange OLEDs with the Phosphorescent Cyclometalated Trinuclear Pt(II) Complex. ACS Appl. Mater. Interfaces 2018, 10, 10227–10235. 10.1021/acsami.7b18330. 29504742

[ref21] Shafikov M. Z.; Daniels R.; Pander P.; Dias F. B.; Williams J. A. G.; Kozhevnikov V. N. Dinuclear Design of a Pt(II) Complex Affording Highly Efficient Red Emission: Photophysical Properties and Application in Solution-Processible OLEDs. ACS Appl. Mater. Interfaces 2019, 11, 8182–8193. 10.1021/acsami.8b18928. 30753060

[ref22] Hao Z.; Zhang K.; Chen K.; Wang P.; Lu Z.; Zhu W.; Liu Y. More Efficient Spin-Orbit Coupling: Adjusting the Ligand Field Strength to the Second Metal Ion in Asymmetric Binuclear Platinum(II) Configurations. Dalton Trans. 2020, 49, 8722–8733. 10.1039/D0DT00939C. 32555914

[ref23] Sun Y.; Chen C.; Liu B.; Guo Y.; Feng Z.; Zhou G.; Chen Z.; Yang X. Efficient Dinuclear Pt(II) Complexes Based on the Triphenylphosphine Oxide Scaffold for High Performance Solution-Processed OLEDs. J. Mater. Chem. C 2021, 9, 5373–5378. 10.1039/D0TC05965J.

[ref24] Sun Y.; Liu B.; Jiao B.; Guo Y.; Chen X.; Zhou G.; Chen Z.; Yang X. Highly Efficient Solution-processed Pure Yellow OLEDs Based on Dinuclear Pt(II) Complexes. Mater. Chem. Front. 2021, 5, 5698–5705. 10.1039/D1QM00507C.

[ref25] Wang S. F.; Fu L.-W.; Wei Y.-C.; Liu S.-H.; Lin J.-A.; Lee G.-H.; Chou P.-T.; Huang J.-Z.; Wu C.-I.; Yuan Y.; Lee C.-S.; Chi Y. Near-Infrared Emission Induced by Shortened Pt-Pt Contact: Diplatinum(II) Complexes with Pyridyl Pyrimidinato Cyclometalates. Inorg. Chem. 2019, 58, 13892–13901. 10.1021/acs.inorgchem.9b01754. 31565936

[ref26] Saito K.; Hamada Y.; Takahashi H.; Koshiyama T.; Kato M. Organic Light-Emitting Diodes Based on a Binuclear Platinum(II) Complex. Jpn. J. Appl. Phys. 2005, 44, L500–L501. 10.1143/JJAP.44.L500.

[ref27] Xiong W.; Meng F.; Tan H.; Wang Y.; Wang P.; Zhang Y.; Tao Q.; Su S.; Zhu W. Dinuclear Platinum Complexes Containing Aryl-Isoquinoline and Oxadiazole-Thiol with an Efficiency of Over 8.8%: In-Depth Investigation of the Relationship Between Their Molecular Structure and Near-Infrared Electroluminescent Properties in PLEDs. J. Mater. Chem. C 2016, 4, 6007–6015. 10.1039/C6TC00825A.

[ref28] Rajakannu P.; Lee W.; Park S.; Kim H. S.; Mubarok H.; Lee M. H.; Yoo S. Molecular Engineering for Shortening the Pt···Pt Distances in Pt(II) Dinuclear Complexes and Enhancing the Efficiencies of these Complexes for Application in Deep-Red and Near-IR OLEDs. Adv. Funct. Mater. 2023, 33, 2211853 10.1002/adfm.202211853.

[ref29] Yu J.; Yang X.; Chen J.; Liu D.; Cao L.; Tan H.; Zhu W. Achieving Near-infrared Electroluminescence Around 780 nm Based on Butterfly-shaped dinuclear platinum(II) complexes. J. Mater. Chem. C 2023, 11, 12384–12391. 10.1039/d3tc01830j.

[ref30] Zhang Y.; Miao J.; Xiong J.; Li K.; Yang C. Rigid Bridge-Confined Double-Decker Platinum(II) Complexes Towards High-Performance Red and Near-Infrared Electroluminescence. Angew. Chem., Int. Ed. 2022, 61, e202113718 10.1002/anie.202113718. 34734464

[ref31] Wang L.; Wen Z.; Xu Y.; Zhang Y.; Miao J.; Chen Z.; Li K. High-efficiency and Stable Red to Near-infrared Organic Light-emitting Diodes Using Dinuclear Platinum(II) Complexes. Mater. Chem. Front. 2023, 7, 873–880. 10.1039/D2QM01163H.

[ref32] Xue M.; Lam T.-L.; Cheng G.; Liu W.; Low K.-H.; Du L.; Xu S.; Hung F.-F.; Phillips D. L.; Che C. M. Exceedingly Stable Luminescent Dinuclear Pt(II) Complexes with Ditopic Formamidinate Bridging Ligands for High-Performance Red and Deep-Red OLEDs with LT97 up to 2446 h at 1000 cdm^–2^. Adv. Optical Mater. 2022, 10, 2200741 10.1002/adom.202200741.

[ref33] Ma B.; Djurovich P. I.; Garon S.; Alleyne B.; Thompson M. E. Platinum Binuclear Complexes as Phosphorescent Dopants for Monochromatic and White Organic Light-Emitting Diodes. Adv. Funct. Mater. 2006, 16, 2438–2446. 10.1002/adfm.200600614.

[ref34] Su N.; Meng F.; Chen J.; Wang Y.; Tan H.; Su S.; Zhu W. Near-infrared Emitting Pyrazole-Bridged Binuclear Platinum Complexes: Synthesis, Photophysical and Electroluminescent Properties in PLEDs. Dyes Pigm. 2016, 128, 68–74. 10.1016/j.dyepig.2016.01.014.

[ref35] Lo K. W.; Tong G. S. M.; Cheng G.; Low K. H.; Che C. M. Dinuclear PtII Complexes with Strong Blue Phosphorescence for Operationally Stable Organic Light-Emitting Diodes with EQE up to 23% at 1000 cdm^–2^. Angew. Chem., Int. Ed. 2022, 61, e202113718 10.1002/anie.202115515. 34939273

[ref36] Zhang L.-Y.; Xu L.-J.; Wang J.-Y.; Zeng X.-C.; Chen Z.-N. Photoluminescence and Electroluminescence of Cationic PtAu_2_ Heterotrinuclear Complexes with Aromatic Acetylides. Dalton Trans. 2017, 46, 865–874. 10.1039/C6DT04249J. 28001160

[ref37] Xu L.-J.; Zeng X.-C.; Wang J.-Y.; Zhang L.-Y.; Chi Y.; Chen Z.-N. Phosphorescent PtAu_2_ Complexes with Differently Positioned Carbazole-Acetylide Ligands for Solution-Processed Organic Light-Emitting Diodes with External Quantum Efficiencies of over 20%. ACS Appl. Mater. Interfaces 2016, 8, 20251–20257. 10.1021/acsami.6b06707. 27430486

[ref38] Zeng X.-C.; Wang J.-Y.; Xu L.-J.; Wen H.-M.; Chen Z.-N. Solution-Processed Oleds Based on Phosphorescent PtAu_2_ Complexes with Phenothiazine-Functionalized Acetylides. J. Mater. Chem. C 2016, 4, 6096–6103. 10.1039/C6TC01539E.

[ref39] Li Y.-P.; Fan X.-X.; Wu Y.; Zeng X.-C.; Wang J.-Y.; Wei Q.-H.; Chen Z.-N. High-Efficiency Organic Light-Emitting Diodes of Phosphorescent PtAg_2_ Heterotrinuclear Acetylide Complexes Supported by Triphosphine. J. Mater. Chem. C 2017, 5, 3072–3078. 10.1039/C7TC00382J.

[ref40] Shu H.-X.; Wang J.-Y.; Zhang Q.-C.; Chen Z.-N. Photophysical and Electroluminescent Properties of PtAg_2_ Acetylide Complexes Supported with meso- and rac-Tetraphosphine. Inorg. Chem. 2017, 56, 9461–9473. 10.1021/acs.inorgchem.7b00452. 28441021

[ref41] Lin Y.-D.; Lu C.-W.; Su H.-C. Long-Wavelength Light-Emitting Electrochemical Cells: Materials and Device Engineering. Chem.—Eur. J. 2023, 29, e202202985 10.1002/chem.202202985. 36346637

[ref42] Bai R. B.; Meng X. W.; Wang X. X.; He L. Color-Stable, Efficient, and Bright Blue Light-Emitting Electrochemical Cell Using Ionic Exciplex Host. Adv. Funct. Mater. 2020, 31, 1907169 10.1002/adfm.202007167.

[ref43] Santander-Nelli M.; Boza B.; Salas F.; Zambrano D.; Rosales L.; Dreyse P. Theoretical Approach for the Luminescent Properties of Ir(III) Complexes to Produce Red-Green-Blue LEC Devices. Molecules 2022, 27, 2623 10.3390/molecules27092623. 35565982 PMC9104581

[ref44] Fuertes S.; Mardegan L.; Martínez I.; Ventura S.; Ara I.; Tordera D.; Bolink H. J.; Sicilia V. Green Light-Emitting Electrochemical Cells Based on Platinum(II) Complexes with a Carbazole-Appended Carbene Ligand. J. Mater. Chem. C 2022, 10, 15491–15500. 10.1039/D2TC02539F.

[ref45] Lu W.; Chan M. C. W.; Zhu N. Y.; Che C. M.; Li C. N.; Hui Z. Structural and Spectroscopic Studies on Pt···Pt and π-π Interactions in Luminescent Multinuclear Cyclometalated Platinum(II) Homologues Tethered by Oligophosphine Auxiliaries. J. Am. Chem. Soc. 2004, 126, 7639–7651. 10.1021/ja039727o. 15198612

[ref46] Weber K. T.; Karikis K.; Weber M. D.; Coto P. B.; Charisiadis A.; Charitaki D.; Charalambidis G.; Angaridis P.; Coutsolelos A. G.; Costa R. D. Cunning Metal Core: efficiency/Stability Dilemma in Metallated Porphyrin Based Light-emitting Electrochemical Cells. Dalton Trans. 2016, 45, 13284–13288. 10.1039/C6DT02293F. 27363542

[ref47] Shafikov M. Z.; Tang S.; Larsen C.; Bodensteiner M.; Kozhevnikov V. N.; Edman L. An Efficient Heterodinuclear Ir(III)/Pt(II) Complex: Synthesis, Photophysics and Application in Light-emitting Electrochemical Cells. J. Mater. Chem. C 2019, 7, 10672–10682. 10.1039/C9TC02930C.

[ref48] Cinninger L. M.; Bastatas L. D.; Shen Y. L.; Holliday B. J.; Slinker J. D. Luminescent Properties of a 3,5-Diphenylpyrazole Bridged Pt(II) Dimer. Dalton Trans. 2019, 48, 9684–9691. 10.1039/C9DT00795D. 30938381

[ref49] Sicilia V.; Fuertes S.; Chueca A. J.; Arnal L.; Martín A.; Perálvarez M.; Botta C.; Giovanella U. Highly Efficient Platinum-based Emitters for Warm White Light Emitting Diodes. J. Mater. Chem. C 2019, 7, 4509–4516. 10.1039/C9TC00747D.

[ref50] Sicilia V.; Arnal L.; Chueca A. J.; Fuertes S.; Babaei A.; Igual Muñoz A. M.; Sessolo M.; Bolink H. J. Highly Photoluminescent Blue Ionic Platinum-Based Emitters. Inorg. Chem. 2020, 59, 1145–1152. 10.1021/acs.inorgchem.9b02782. 31880921

[ref51] Fuertes S.; Chueca A. J.; Arnal L.; Martín A.; Giovanella U.; Botta C.; Sicilia V. Heteroleptic Cycloplatinated N-heterocyclic Carbene Complexes: A New Approach to Highly Efficient Blue-Light Emitters. Inorg. Chem. 2017, 56, 4829–4839. 10.1021/acs.inorgchem.6b02826. 28387513

[ref52] Sicilia V.; Arnal L.; Escudero D.; Fuertes S.; Martin A. Chameleonic Photo- and Mechanoluminescence in Pyrazolate-Bridged NHC Cyclometalated Platinum Complexes. Inorg. Chem. 2021, 60, 12274–12284. 10.1021/acs.inorgchem.1c01470. 34339189 PMC8892954

[ref53] Falvello L. R.; Fornies J.; Martin A.; Sicilia V.; Villarroya P. Synthesis and Reactivity of the Neutral Pyrazolate Complexes [M_2_{CH_2_C_6_H_4_P(*o*-tolyl)_2_-κC,P}_2_(μ-Rpz)_2_] (M = Pd, Pt; Rpz = Pz, 3,5-dmpz, 4-Mepz) toward AgClO_4_. Molecular Structure of [Pt_2_Ag{CH_2_C_6_H_4_P(*o*-tolyl)_2_-κC,P}_2_(μ-4-Mepz)_2_]ClO_4_. Organometallics 2002, 21, 4604–4610. 10.1021/om0203835.

[ref54] Baya M.; Belio U.; Fornies J.; Martin A.; Peralvarez M.; Sicilia V. Neutral Benzoquinolate Cyclometalated Platinum(II) Complexes as Precursors in the Preparation of Luminescent Pt-Ag Complexes. Inorg. Chim. Acta 2015, 424, 136–149. 10.1016/j.ica.2014.07.059.

[ref55] Martin A.; Belio U.; Fuertes S.; Sicilia V. Luminescent PtAg Clusters Based on Neutral Benzoquinolate Cyclometalated Platinum Complexes. Eur. J. Inorg. Chem. 2013, 2013, 2231–2247. 10.1002/ejic.201201528.

[ref56] Escudero D. Photodeactivation Channels of Transition Metal Complexes: A Computational Chemistry Perspective. In Transition Metals in Coordination Environments: Computational Chemistry and Catalysis Viewpoints; Broclawik E., Borowski T., Radoń M., Eds.; Springer International Publishing: Cham, 2019, pp 259–287.

[ref57] Roberts M.; King S.; Cass M.; Pintani M.; Coward C.; Akino N.; Nakajima H.; Anryu M. 56.1: *Invited Paper*: Excited State Interactions in P-OLEDs: Implications For Efficiency And Lifetime. SID Symp. Dig. Tech. Pap. 2011, 42, 820–821. 10.1889/1.3621456.

[ref58] VanSlyke S. A.; Chen C. H.; Tang C. W. Organic Electroluminescent Devices with Improved Stability. Appl. Phys. Lett. 1996, 69, 2160–2162. 10.1063/1.117151.

[ref59] van Reenen S.; Matyba P.; Dzwilewski A.; Janssen R. A. J.; Edman L.; Kemerink M. A. A Unifying Model for the Operation of Light-Emitting Electrochemical Cells. J. Am. Chem. Soc. 2010, 132, 13776–13781. 10.1021/ja1045555. 20831189

