# Supplementary material for: Pyrazolate-Bridged NHC Cyclometalated [Pt2] Complexes and [Pt2Ag(PPh3)]+ Clusters in Electroluminescent Devices
Source: Inorg Chem. 2024 Apr 8;63(16):7275–85. doi: 10.1021/acs.inorgchem.4c00105 (PMC11040726; doi:10.1021/acs.inorgchem.4c00105)
Supplement: Supplementary file 1 — ic4c00105_si_001.pdf [file ic4c00105_si_001.pdf]

SUPPORTING INFORMATION

Pyrazolate-Bridged NHC Cyclometalated $[Pt_2]$ complexes and $[Pt_2Ag(PPh_3)]^+$ Clusters in Electroluminescent Devices

Jorge Roy, Michele Forzatti, Lorenzo Arnal, Antonio Martín, Sara Fuertes, Daniel Tordera, and Violeta Sicilia**

E-mail: daniel.tordera@uv.es

E-mail: sicilia@unizar.es

CONTENTS

1. Experimental Section S2

1.1 General procedures, materials and instrumentation..S2

1.2 Computational methods. S2

1.3 X-ray Structure determinations. Experimental procedures and refinement. S3

1.4 OLEDs fabrication and characterization..S6

1.5 LECs fabrication and characterization..S7

1.6 Synthesis of new compounds..S8

2. Spectra for characterization S11

3. Full description of the single crystal X-ray structures of compounds 1a-1c. S32

4. Absorption data and theoretical calculations for the $[Pt_2Ag(PPh_3)]^+$ clusters........S34

5. Emission properties of the $[Pt_2Ag(PPh_3)]^+$ clusters..S37

6. Devices characterization and performances . S40

7. References S43

1. Experimental Section

1.1 General procedures, materials and instrumentation.

Compounds [{Pt(EtO_2C-C^C*)(μ-Rpz)}$_2$](RpzH = pzH **A**,[1] 4-MepzH **B**,[2] 3,5-dppzH **C**[3],[2]) and [Ag($OClO_3$)(PPh_3)],[3] were prepared as described elsewhere. PPh_3 and $AgPF_6$ were purchased from Sigma-Aldrich, and fluoroChem respectively and used as received.

IR spectra were recorded on a Perkin-Elmer Spectrum 100 FT-IR Spectrometer (ATR in the range 250-4000 cm-1). Mass spectral analyses were performed with a Microflex MALDI-TOF Bruker or an Autoflex III MALDI-TOF Bruker instruments. C, H, and N analyses were carried out in a Perkin-Elmer 2400 CHNS analyser or Thermo Flash 1112. ^{1}H, ^{13}C{^{1}H}, ^{31}P{^{1}H}, ^{195}Pt{^{1}H} NMR spectra were recorded on a Bruker NEO 400 and 500 MHz instruments using the standard references: $SiMe_4$ for ^{1}H and ^{13}C, Na_2PtCl_6 in D_2O for ^{195}Pt, 85 % H_3PO_4 for ^{31}P and $CFCl_3$ for ^{19}F. J are given in Hz and δ are given in ppm; assignments are based on ^{1}H-^{1}H COSY experiments and ^{1}H-^{13}C HSQC and HMBC experiments. Unless otherwise indicated, all measurement were performed at r.t.

UV-visible spectra were recorded on a Unicam UV4 spectrophotometer. Steady-state photoluminescence spectra were recorded on a Jobin-Yvon Horiba Fluorolog FL-3-11 Tau 3 spectrofluorimeter. Phosphorescence lifetimes were recorded with a Fluoromax phosphorimeter accessory containing a UV xenon flash tube. Nanosecond lifetimes were recorded with a Datastation HUB-B with a nanoLED controller and software DAS6. NanoLEDs of 340 nm and 370 nm were employed for lifetimes measurements. The lifetime data were fitted using the Jobin-Yvon software package and the Origin Pro 8 program. Solid state Quantum Yields (QY) were measured using the Hamamatsu Absolute PL Quantum Yield Measurement System C11347-11. The absorbance and photoluminescence spectra of thin-films were measured with an Avantes AvaSpec-2048L spectrometer equipped with a Avantes AvaLight-DS-S-BAL deuterium halogen light source and optic fibres. For photoluminescence measurements, films were illuminated with a diode laser of integrated optics, with an emission wavelength of 365 nm.

1.2 Computational methods.

Density functional calculations were carried out on the ground (S_0) state with the Gaussian 16[4] suite of programs, using the M06 hybrid density functional[5] (MUE (M06) = 2.48

kcal/mol[6]) together with Grimme's D3 dispersion correction.[7] The ECP-60-mwb for platinum and ECP-28-mwb, for silver, pseudopotential[8] was used, and the 6-31G(d)[9,10] basis sets were used for all other atoms. In order to facilitate the theoretical study, we have done a simplification on the real system, we have modelled the ethanoate substituent on the cyclometalated ligand as an acetate. General geometry optimizations were performed without any symmetry restriction and in THF using the polarizable continuum model (PCM).[11] Frequency calculations were performed in order to determine the nature of the stationary points found in S_o no imaginary frequencies for minima. Mulliken population analysis was carried out as implemented in Gaussian 16 package.[4] ChemissianLab program package was used for analysis and graphic representation of molecular structures and orbitals and for Mayer Bond Order analysis. Atomic charges were calculated by using the NBO analysis option as incorporated in Gaussian 16.[4]

1.3 X-ray Structure determinations. Experimental procedures and refinement.

Crystal data and other details of the structure analyses are presented in Table S1. Suitable crystals of 1a $2Me_2CO$,1b $0.25Me_2CO$, 1c $0.35C_7H_8$ for X-ray diffraction studies were obtained by slow diffusion of *n*-hexane into saturated solutions of 1a, 1b in acetone or toluene into saturated solution of 1c in THF. Crystals were mounted at the end of quartz fibers and the data collection was performed at 100 K temperature. The radiation used in all cases was graphite monochromated Mo$K\alpha$ (λ = 0.71073 Å). X-ray intensity data were collected on an Oxford Diffraction Xcalibur diffractometer. The diffraction frames were integrated and corrected from absorption by using the CrysAlis RED program.[12] The structures were solved by Patterson and Fourier methods and refined by full-matrix least squares on F^2 with SHELXL.[13] All non-hydrogen atoms were assigned anisotropic displacement parameters and refined without positional constraints, except as noted below. All hydrogen atoms were constrained to idealized geometries and assigned isotropic displacement parameters equal to 1.2 times the Uiso values of their attached parent atoms. For 1a $2Me_2CO$, the CH_3 fragment of one of the ethyl residues is disordered over two positions which were refined with 0.5 partial occupancy each. One of the perchlorate anions has three of hits oxygen atoms disordered over two positions which were refined with 0.6/0.4 partial occupancy. One of the crystallization acetone molecules is disordered over two positions with 0.5/0.5 partial

occupancy. Some soft restrains were used in the interatomic distances for some of the disordered atoms. For 1b $0.25Me_2CO$, the crystal quality is not very good and it did not diffract intensely at medium and high angles. This was probably due to poor packing caused by the shape of the molecules. Thus, voids appear in the structure, which are treated using the SQUEEZE procedure implemented in PLATON.[14] One of the perchlorates has three of its oxygen atoms disordered in two positions which are refined with 0.6/0.4 occupancies. The other perchlorate is disordered in two different, but close, positions that refine to 0.68/0.32 occupancies. An acetone molecule, although diffuse, can be modelled with occupancy 0.5. Restrains were used in some of the geometrical parameters of the disordered moieties. For 1c $0.35C_7H_8$, the solvent toluene molecule lies diffusely around an inversion center and the position of its methyl group is disordered. It was not possible to anisotropize the thermal parameters for the C atoms of the toluene moiety. Full-matrix least-squares refinement of these models against *F*2 converged to final residual indices given in Table S1. CCDC Nos. 2309669-2309671 contain the supplementary crystallographic data for **1a, 1b** and **1c**.

Table S1: Crystallographic data

	**1a·2Me2CO**	**1b·0.25Me2CO**	**1c·0.35 toluene**
Empirical formula	$C_{56}H_{59}AgClN_8O_{10}PPt_2$	$C_{52.75}H_{52.50}AgClN_8O_{8.25}PPt_2$	$C_{76.45}H_{65.80}AgClN_8O_8PPt_2$
Formula weight	1568.58	1494.99	1789.04
Crystal system	Monoclinic	Monoclinic	Triclinic
Space group	P 21/n	P 21/n	P -1
a (Å)	13.70329(16)	14.1667(4)	12.1049(3)
b (Å)	26.6973(3)	26.4853(7)	12.3896(3)
c (Å)	31.4337(4)	30.7734(11)	23.4781(6)
α (°)	90	90	89.965(2)
β (°)	91.2386(10)	99.107(3)	76.174(2)
γ (°)	90	90	86.156(2)
Volume ($Å^3$)/Z	11497.0(2) /8	11400.9(6) /8	3410.97(15)
ρ (Mg/m^3)	1.812	1.742	1.742
μ (Mo-Kα)/mm^{-1}	5.331	5.369	4.502
F(000)	6128	5808	1759
Crystal size (mm^3)	0.390 x 0.290 x 0.170	0.300 x 0.130 x 0.110	0.150 x 0.080 x 0.040
Theta range (°)	1.525 to 28.373	1.688 to 28.384	2.475 to 29.418
Reflections collected	90622	89631	45736
Independent reflections [R(int)]	24708 [0.0483]	24722 [0.0919]	16209 [0.0818]
Final R_1,wR_2 [I>2sigma(I)]	0.0396, 0.0787	0.0778, 0.1451	0.0581, 0.0952
R_1,wR_2 (all data)[a]	0.0622, 0.0865	0.1568, 0.1733	0.1126, 0.1131
GOF (F^2)[b]	1.028	1.027	1.016
Largest diff. peak and hole/e.$Å^{-3}$	2.858 and 1.708	1.802 and -1.520	3.390 and -1.465

[a] $R_1 = \sum(|F_o| - |F_c|) / \sum |F_o|$. $wR_2 = [\sum w (F_o^2 - F_c^2)^2 / \sum w(F_o^2)^2]^{1/2}$.

[b] Goodness-of-fit = $[\sum w (F_o^2 - F_c^2)^2 / (n_{obs} - n_{param})]^{1/2}$

1.4 OLED fabrication and characterization.

Aqueous poly(3,4-ethylenedioxythiophene) polystyrene sulfonate (PEDOT:PSS) dispersion was obtained from Clevios™ (P VP AI 4083). 4,4'-Bis(carbazol-9-yl)biphenyl (CBP) and 1,3- bis[3,5-di(pyridin-3-yl)phenyl]benzene (BmPyPhB) were purchased from Luminescence Technology Corp. Pre-patterned 150 nm thick indium tin oxide (ITO)-coated glass plates (3 cm × 3 cm) were used as transparent conductive substrates. They were subsequently cleaned ultrasonically in tap water-detergent, deionized water, and 2-propanol baths for 5 min. After drying, the substrates were placed in a UV–ozone cleaner (Jelight 42–220) for 15 min. The ITO substrates were coated with PEDOT:PSS Al4083 solution (2000 rpm for 60 seconds, resulting in 30 nm). The PEDOT:PSS films were thermally treated at 150 °C for 10 min. The emissive layer consisted of a mixture of CBP and metal complexes at 6 wt.%, and was deposited by spin-coating (1500 rpm, 60 s) from chlorobenzene solutions (10 mg mL^{-1}) to a thickness of 30 nm. These substrates were transferred to the vacuum chamber, where BmPyPhB, Ba and Ag were thermally deposited. The vacuum evaporation rates were 0.1 nm s^{-1} and 0.05 nm s^{-1} for the ETL and cathode, respectively, with the background pressure being around 3×10^{-6} bar. Shadow masks were used during the metal evaporation to obtain a final active area of 6 mm^2. As for OLEDs with **C** as emitting layer, the vacuum evaporation rates of TAPC, mCP, emitting layer and PO-T2T were 0.6 nm s^{-1}, 0.6 nm s^{-1}, 0.01 nm s^{-1} and 0.1 nm s^{-1}, respectively, with the background pressure being around 3×10^{-6} bar.

After full device fabrication, the samples were introduced into a setup for a current density and luminance versus voltage (*JVL*) scan. For this we employed a Keithley 2400 Source-Meter and a photodiode coupled to a Keithley 6485 picoammeter. A LabVIEW program was used to control the Keithleys and to obtain the data. The photodiode was calibrated using a Konica Minolta LS-150 equipped with a 110 close-up lens for the measurement of small areas and controlled through the CS-S20 Data Management Software. Electroluminescence (EL) spectra were recorded by driving the cells with the Keithley 2400 Source-Meter and an optical fibre connected to the Avant spectrometer AvaSpec–2048L. The external quantum efficiency (EQE) of the devices was extracted from the current efficiency and the EL spectra.

1.5 LECs fabrication and characterization.

A solution of the emitter was mixed in a molar ratio of 3.14:1 with the ionic liquid (IL) 1-butyl3-methyl-imidazolium-hexafluorophosphate ($BMIM^{+}PF_{6}^{-}$). The solvent used was dichloromethane (DCM). The final concentration of the emitter in the solution was 20 mg/mL. The same substrates as for the fabrication of OLEDs were used and the same cleaning procedure was followed, but after the ozone treatment, a suspension of PEDOT:PSS CH8000 was used instead. An 80-nm thick film was obtained at 4000 rpm for 60 seconds, which was then annealed on a hotplate at 150 °C for 10 minutes. The active layer solution (emitter:IL) was then spin-coated at 2000 rpm for 60 seconds, resulting in a thickness from 90 nm to 120 nm. Films were covered with a beaker for the first 10-15 seconds of spinning to reduce the fast solvent release and improve the morphology of the film. The films were annealed at 90°C for 45 minutes. Finally, an Al electrode (100 nm) was thermally evaporated on top of the active layer using a shadow mask under inert atmosphere. The final active area of the cells was 6 mm^2. The thickness of the PEDOT:PSS and active layer was determined with an Ambios XP-1 profilometer.

The devices were measured by applying a pulsed current density (50 A m^{-2}) while monitoring the voltage and luminance versus time by using a True Color Sensor MAZeT (MTCSiCT sensor) with a Botest OLT OLED Lifetime-Test system. The applied pulsed current consisted of block waves at a frequency of 1000 Hz with a duty cycle of 50%. Hence, the average current density and voltage were obtained by multiplying the values by the time-on (0.5 ms) and dividing by the total cycle time (1 ms). Electroluminescence (EL) spectra were recorded by driving the cells with the Botest OLT system and an optical fibre connected to the Avant spectrometer AvaSpec–2048L. The external quantum efficiency (EQE) of the devices was extracted from the current efficiency and the EL spectra.

1.6 Synthesis of new compounds

Caution! *Perchlorate salts of metal complexes with organic ligands are potentially explosive. Only small amounts of material should be prepared and these should be handled with great care.*

Synthesis of [{Pt(EtO_2C-C^C*)(μ-pz)}$_2$Ag(PPh_3)]X.

X= ClO_4 (1a). [Ag($OClO_3$)(PPh_3)] (45.87 mg, 0.100 mmol) was added to a stirred pale-yellow suspension of **A** (96.00 mg, 0.100 mmol) in acetone (20 ml) at room temperature in the dark, which turned into a bright yellow solution. The reaction was kept for 1h; then, the solvent was removed under reduced pressure. The residue was treated with Et_2O (30 ml), filtered, and washed to give **1a** as a yellow-greenish solid. Yield: 122.53 mg, 86%. Anal. Calcd for $C_{50}H_{47}AgClN_8O_8PPt_2$: C, 41.35; H, 3.26; N, 7.72. Found: C, 41.04; H, 3.13; N, 7.93. ^{1}H NMR data for **1a** (500 MHz, acetone-*d$_6$*): δ 8.03 (s,$^3J_{7\text{-}Pt}$ = 49.3, 2H, H_7), 7.95 (d,$^3J_{H\text{-}H}$ = 2.0, 2H, pz), 7.83 (d, 2H, pz),7.79 (d, $^3J_{2\text{-}3}$ = 2.1, 2H, H_2), 7.69 (dd, $^3J_{9\text{-}10}$ = 8.15, $^4J_{7\text{-}9}$ = 1.14, 2H, H_9), 7.48 (m, 3H, H_p), 7.30 (m, 6H, H_m), 7.23 (d, 2H, H_{10}), 7.13 (s, 2H, H_3), 6.56-6.41 (m, 8H, H_o and pz), 4.34-4.29 (m, 4H, OCH_2CH_3), 3.33 (s, 6H, H_4), 1.33 (t, $^3J_{H\text{-}H}$ = 7.1, 6H, OCH_2CH_3). ^{13} C{^{1}H} NMR plus HSQC and HMBC data for **1a** (126 MHz, acetone-*d$_6$*): δ 166.7 (s, 2C, C=O), 154.5 (s, $^1J_{C\text{-}Pt}$ = 1265.7, 2C, C_1), 152.9 (s, 2C, C_5), 140.7 and 139.5 (s, 4C, pz), 137.5 (s, 2C, C_7), 133.8 (d, $^2J_{C\text{-}P}$ = 16.2, 6C, C_o), 131.9 (s, 3C, C_p), 130.7 (d, $^1J_{C\text{-}P}$ = 35.6, 3C, C_{ipso}), 130.1 (d, $^3J_{C\text{-}P}$ = 10.3, 6C, C_m), 128.6 and 127.8 (s, 4C, C_6 and C_8), 128.8 (s, 2C, C_9), 125.1 (s, 2C, C_3), 117.6 (s, 2C, C_2), 112.6 (s, 2C, C_{10}), 107.4 (s, 2C, pz), 61.3 (s, 2C, OCH_2CH_3), 37.3 (s, 2C, C_4), 14.6 (s, 2C, OCH_2CH_3).^{31}P {^{1}H} NMR (202 MHz, acetone-*d$_6$*): δ 4.96 (d, $^1J_{P\text{-}^{109}Ag}$ = 715.9, $^1J_{P\text{-}^{107}Ag}$ = 621.6, $^2J_{P\text{-}^{195}Pt}$ = 238.7). ^{195}Pt{^{1}H} NMR (86 MHz, acetone-*d$_6$*, 193 K): δ -3490.4 (dd, $^1J_{Pt\text{-}Ag}$ = 469.4). MS (MALDI +): m/z = 982.58 [{Pt(C^C*)(μ-pz)}$_2$]$^+$, m/z = 1353.70 [{Pt(C^C*)(μ-pz)}$_2$Ag(PPh_3)]$^+$. IR (ATR, cm^{-1}) ν = 1711 (m, C=O), 1077, 623 (s, ClO_4).

X= PF_6 (2a). Ag PF_6 (52.50 mg, 0.207 mmol) was added to a suspension of **A** (203,5 mg, 0.207 mmol) in acetone (50 ml) and the mixture was stirred for 1h at r.t. in the dark. Then a solution of PPh_3 (66.37 mg, 0.248 mmol) in 10 mL of acetone was added to it drop by drop and was left to react for 90 min. Then, the solvent was evaporated to 2 mL and a mixture of Et_2O/MeOH (50 mL/ 3 mL) added to the residue. The resulting solid, **2a** was filtered and washed with Et_2O (10 mL x 2) and cold MeOH (2 mL, 4ºC). Yield: 248.9 mg, 80%. Anal.

Calcd for $C_{50}H_{47}AgF_6N_8O_4P_2Pt_2$: C, 40.09; H, 3.16; N, 7.48. Found: C,40.42; H, 3.03; N, 7.37: IR (ATR, cm^{-1}) ν = 834, 556 (s, PF_6^-). $^{31}P\{^1H\}$ NMR (202 MHz, acetone-d_6): δ(PF_6) -144.24 (sept, $^1J_{P-F}$ = 707.0 Hz). $^{19}F\{^1H\}$ NMR (471 MHz, acetone-d_6): δ -72.64 (d).

Synthesis of [{Pt(EtO$_2$C-C^C*)(*μ*-4-Mepz)}$_2$Ag(PPh$_3$)]X

X= ClO$_4$ (1b). Complex **1b** was obtained as a yellow-greenish solid and prepared similarly to **1a.** [Ag(OClO$_3$)(PPh$_3$)] (35.44 mg, 0.075 mmol) and **B** (76.30 mg, 0.075 mmol). Yield: 92.30 mg, 83%. Anal. Calcd for $C_{52}H_{51}AgClN_8O_8PPt_2$: C, 42.19; H, 3.47; N, 7.57. Found: C, 41.98; H, 3.33; N, 7.69. ^1H NMR data for **1b** (500 MHz, acetone-d_6): δ 8.06 (d, $^4J_{7-9}$ = 1.7, $^3J_{7-Pt}$ = 50.1, 2H, H_7), 7.81 (d,$^3J_{2-3}$ = 2.0, 2H, H_2), 7.72 (s, 2H, 4-Mepz), 7.68 (dd, $^3J_{9-10}$ = 8.13, 2H, H_9), 7.57 (s, 2H, 4-Mepz), 7.47 (m, 3H, H_p), 7.30 (m, 6H, H_m), 7.22 (d, 2H, H_{10}), 7.12 (d, 2H, H_3), 6.53-6.48 (m, 6H, H_o), 4.37-4.26 (m, 4H, OCH_2CH$_3$), 3.35 (s, 6H, H_4), 2.14 (s, 6H, Me, 4-Mepz), 1.34 (t, $^3J_{H-H}$ = 7.2, 6H, OCH$_2$CH_3).$^{13}C\{^1H\}$ NMR plus HSQC and HMBC data for **1b** (126 MHz, acetone-d_6): δ 166.7 (s, 2C, C=O), 154.8 (s, $^1J_{C-Pt}$ = 1258.3, 2C, C_1), 152.9 (s, 2C, C_5), 140.6 and 139.1 (s, 4C, 4-Mepz), 137.5 (s, 2C, C_7), 133.8 (d, $^2J_{C-P}$ = 15.1, 6C, C_o), 131.9 (s, 3C, C_p),130.7 (d, $^1J_{C-P}$ = 34.5, 3C, C_{ipso}), 130.1 (d, 6C, C_m), 128.9 and 127.7 (s, 4C, C_6 and C_8), 128.7 (s, 2C, C_9), 125.0 (s, 2C, C_3), 117.6 (s, 2C, C_2), 117.5 (s, 2C, 4-Mepz), 112.5 (s, 2C, C_{10}), 61.3 (s, 2C, OCH$_2$CH$_3$), 37.3 (s, 2C, C_4), 14.6 (s,2C, OCH$_2$$CH_3$), 9.4 (s,2C, Me, 4-Mepz).$^{31}P\{^1H\}$ NMR (202 MHz, acetone-d_6): δ 4.94 (d, $^1J_{P-109Ag}$ = 713.4, $^1J_{P-107Ag}$ = 619.3, $^2J_{P-195Pt}$ = 243.6).$^{195}Pt\{^1H\}$ NMR (86 MHz, acetone-d_6, 193 K): δ -3487.7 (dd, $^1J_{Pt-Ag}$ = 460.7). MS (MALDI +): m/z =1010.58 [{Pt(C^C*)(*μ*-4-Mepz)}$_2$]$^+$, m/z =1119.53 [{Pt(C^C*)(*μ*-4-Mepz)}$_2$Ag]$^+$, m/z =1381.70. [{Pt(C^C*)(*μ*-4-Mepz)}$_2$Ag(PPh$_3$)]$^+$. IR (ATR, cm^{-1}) ν = 1705 (m, C=O), 1704, 623 (s, ClO$_4$).

X= PF$_6$ (2b). Complex **2b** was obtained as a yellow-greenish solid and prepared similarly to **2a.** Ag PF$_6$ (36.00 mg, 0.143 mmol); **B** (144.2 mg, 0.143 mmol); PPh$_3$ (46.27 mg, 0.171 mmol). Yield: 169.5 mg, 78%. Anal. Calcd for $C_{52}H_{51}AgF_6N_8O_4P_2Pt_2$: C, 40.93; H, 3.37; N, 7.34. Found: C, 41.28; H, 3.16; N, 7.11: IR (ATR, cm^{-1}) ν =; 832, 556 (s, PF_6^-). $^{31}P\{^1H\}$ NMR (202 MHz, acetone-d_6): δ(PF_6) -144.24 (sept, $^1J_{P-F}$ = 707.0 Hz). $^{19}F\{^1H\}$ NMR (471 MHz, acetone-d_6): δ -72.64 (d).

Synthesis of [{Pt(EtO$_2$C-C^C*)(*μ*-3,5-dppz)}$_2$Ag(PPh$_3$)]X.

X= ClO$_4$ (1c). Complex **1c** was obtained as a yellow-greenish solid and prepared similarly to **1a.** [Ag(OClO$_3$)(PPh$_3$)] (33.93 mg, 0.072 mmol) and **C** (93.00 mg, 0.072 mmol). Yield:

80.90 mg, 82%. Anal. Calcd for $C_{74}H_{63}AgClN_8O_8PPt_2$: C, 50.59; H, 3.61; N, 6.38. Found: C, 50.10; H, 3.41; N, 6.43. ^{1}H NMR data for **1c** (500 MHz, acetone-d_6): δ 8.40 (d, $^3J_{H-H}$ = 6.5, 4H, dppz), 7,94 (s, 4H, dppz), 7.82 (s, $^3J_{H-Pt}$ =47.1, 2H, H_7), 7.62-7,13 (m, 29H, H_{PPh3}, $H_{c\text{^}c*}$ and dppz), 6.96 (d, $^3J_{H-H}$ = 6.5, 2H, dppz), 6.90 (s, 2H, H_2), 6.39 (s, 4H, dppz), 4.38-4.30 (m, 4H, OCH_2CH_3), 3.33 (s, 6H, H_4), 1.39 (t, $^3J_{H-H}$ = 7.0, 6H, OCH_2CH_3). ^{13}C{^{1}H} NMR plus HSQC and HMBC data for **1c** (126 MHz, acetone-d_6): δ 166.4 (s, 2C, C=O), 151.8 (s, 2C, C_1), 138.7 (s, 2C, C_7), 134.5 (s, 2C, dppz), 133.8 (s, 6C, C_o), 133.7 (s, 2C, dppz), 132.1 (s, 3C, C_p), 130.1 (s, 6C, C_m), 129.5 (s, 2C, C_8), 129.2 (s, 2C, dppz), 128.7 (s, 2C, C_9), 127.6 (s, 2C, C_o), 127.3 (s, 2C, C_o), 112.5 (s, 2C, C_2), 106.8 (s, 2C, $C_{C\text{^}C*}$),61.4 (s, 2C, OCH_2CH_3), 36.5 (s, 2C, C_4), 14.8 (s,2C, OCH_2CH_3). ^{31}P{^{1}H} NMR (202 MHz, acetone-d_6): δ 4.18 (d, $^1J_{P-Ag}$ = 668.9, $^2J_{P-195Pt}$ = 206.7).^{195}Pt{^{1}H} NMR (86 MHz, acetone-d_6, 193 K): δ -3404.0 (dd, $^1J_{Pt-Ag}$ = 465.1).MS (MALDI +): m/z =1287.75 [{Pt(C^C*)(μ-3,5-dppz)}$_2$]$^+$, m/z =1395.71 [{Pt(C^C*)(μ-3,5-dppz)}$_2$Ag]$^+$, m/z =1657.94 [{Pt(C^C*)(μ-3,5-dppz)}$_2$Ag(PPh_3)]$^+$. IR (ATR, cm^{-1}) ν =1707 (m, C=O), 1702, 622 (s, ClO_4).

2. Spectra for characterization

(a)

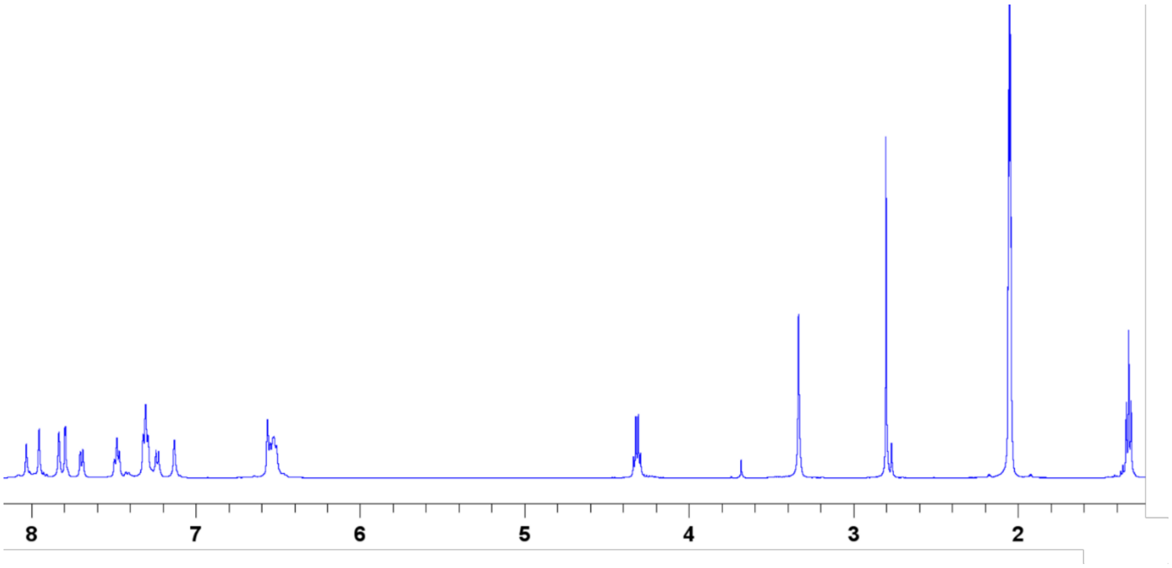

(b)

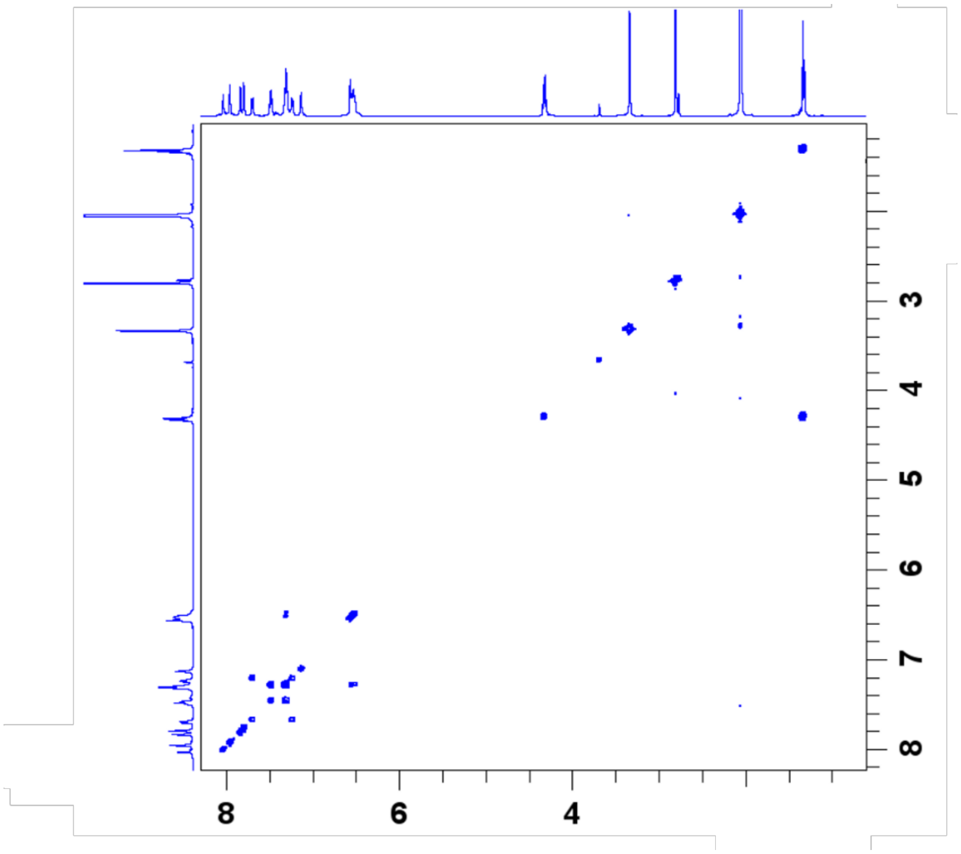

(c)

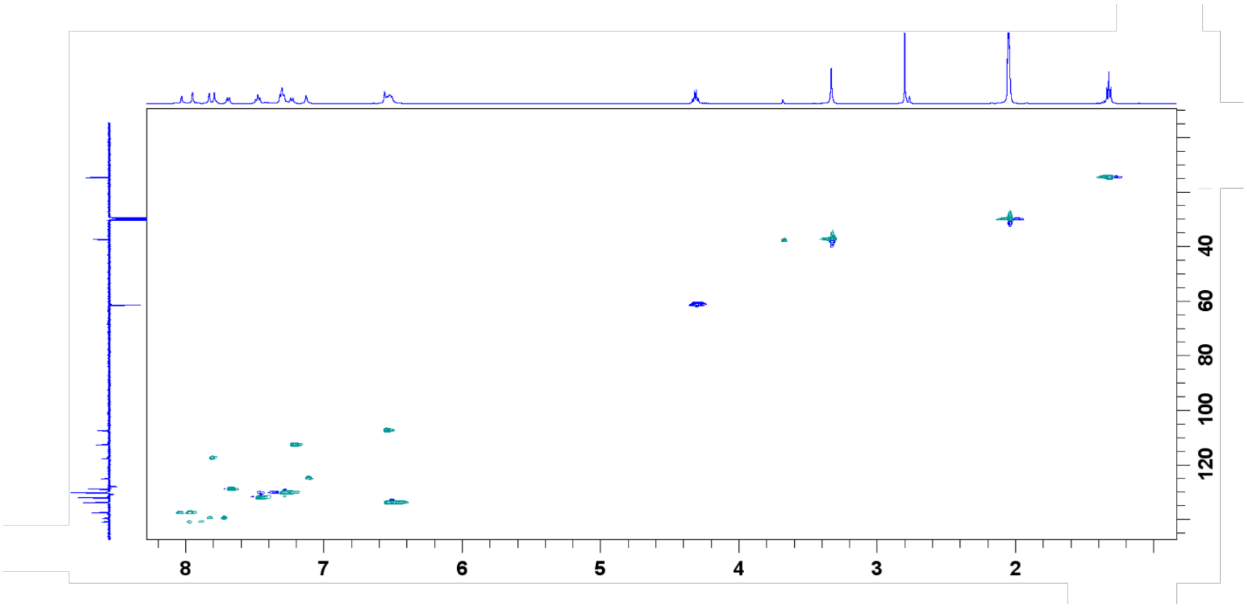

(d)

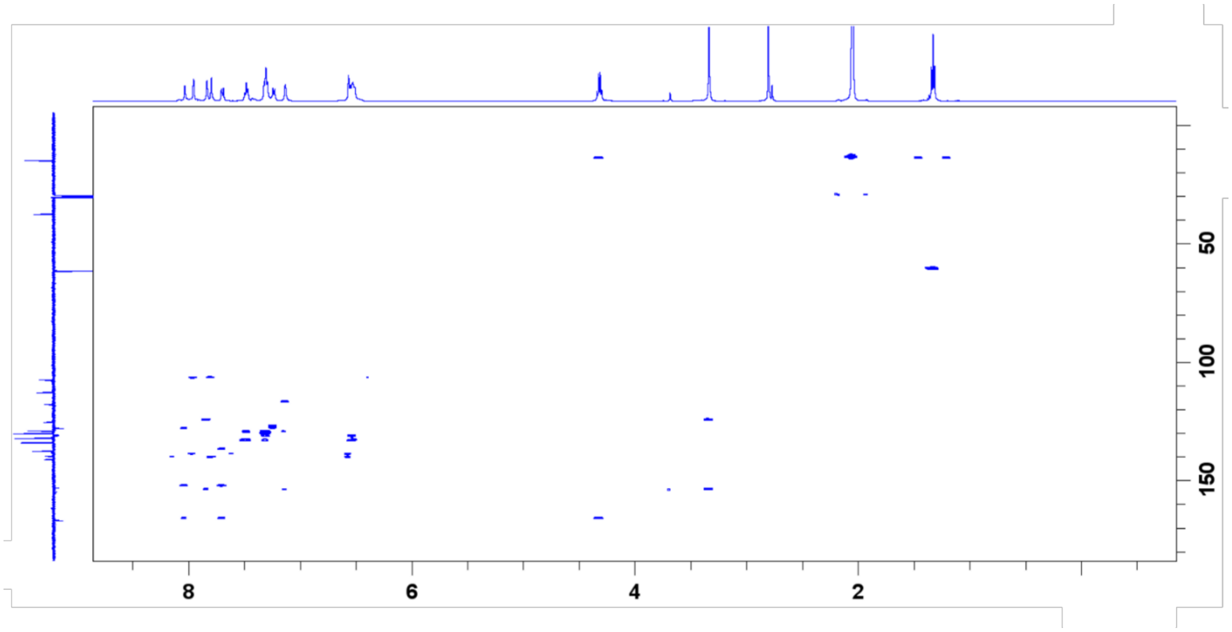

(e)

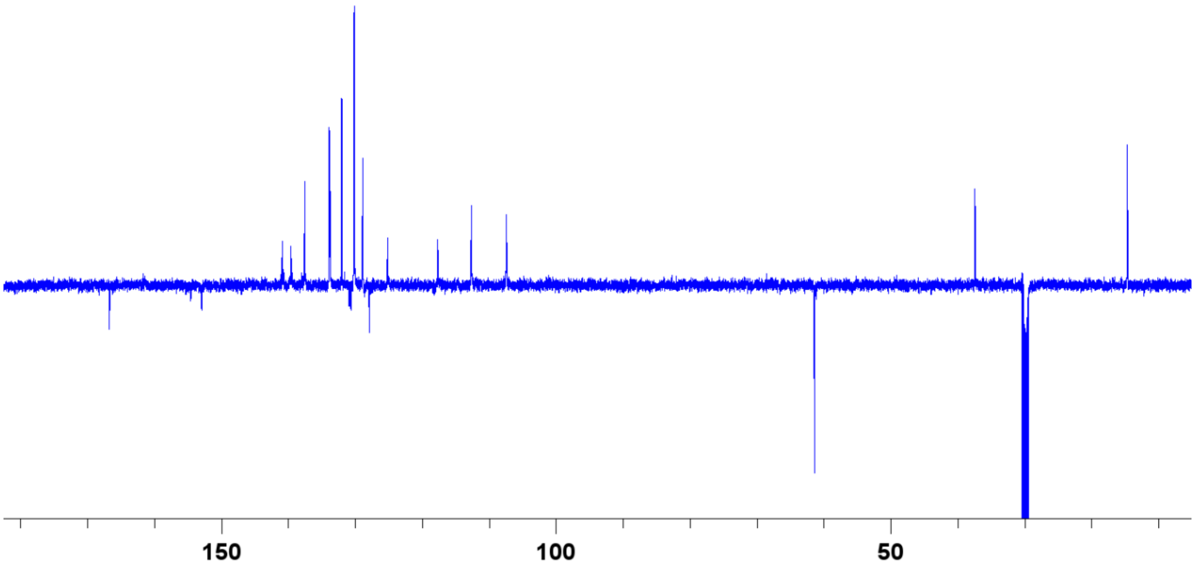

(f)

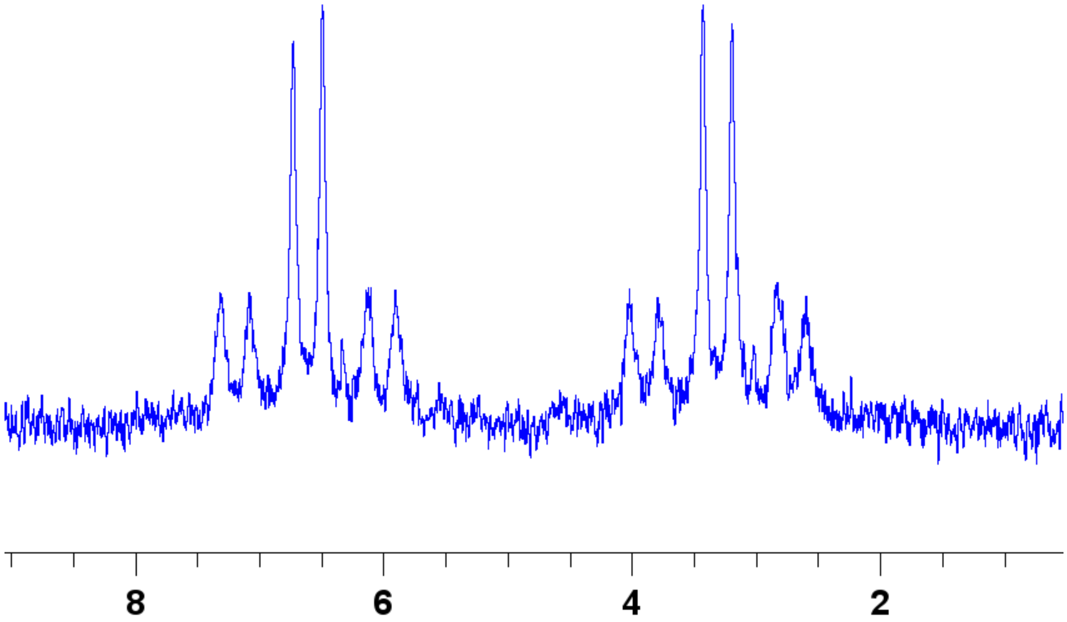

.

(g)

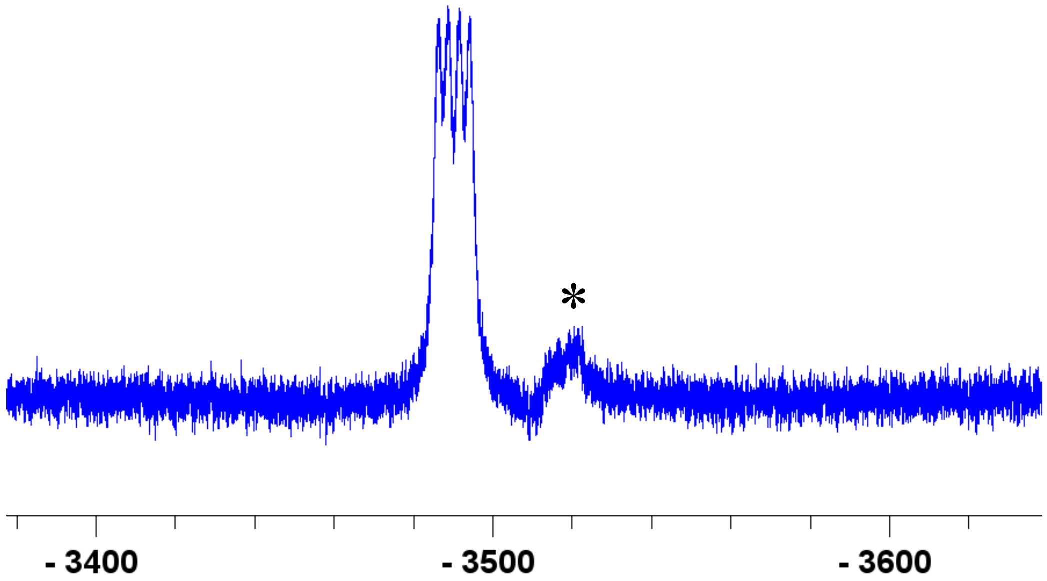
*
- 3400
- 3500
- 3600

(h)

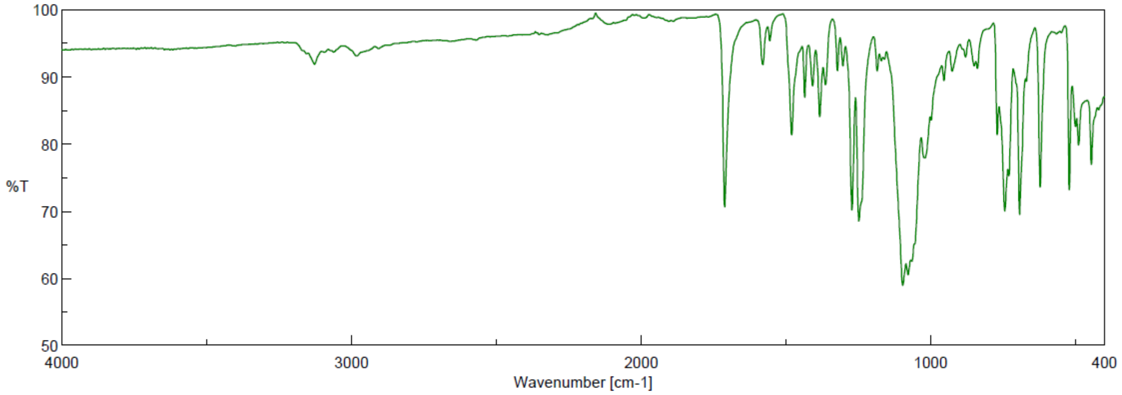
100
90
80
70
60
50
%T
4000
3000
2000
1000
400
Wavenumber [cm-1]

(i)

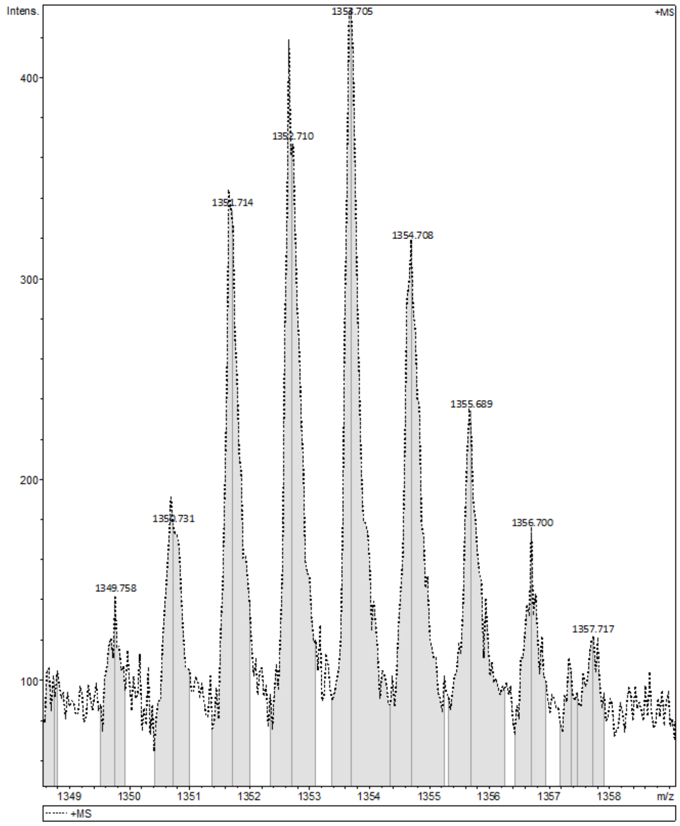

Figure S1: a. ^{1}H NMR spectrum of **1a** in acetone- d_6. **b.** ^{1}H-^{1}H COSY NMR spectrum of **1a** in acetone- d_6. **c.** ^{1}H-^{13}C HSQC NMR spectrum of **1a** in acetone- d_6. **d.** ^{1}H-^{13}C HMBC NMR spectrum of **1a** in acetone- d_6. **e.** ^{13}C{^{1}H} APT NMR spectrum of **1a** in acetone- d_6. **f.** ^{31}P{^{1}H} NMR spectrum of **1a** in acetone- d_6. **g.** ^{195}Pt{^{1}H} NMR spectrum of **1a** at 193K in acetone- d_6. * *syn* isomer. **h**. IR spectrum of **1a**. **i**. Mass spectrum of **1a**.

(a)

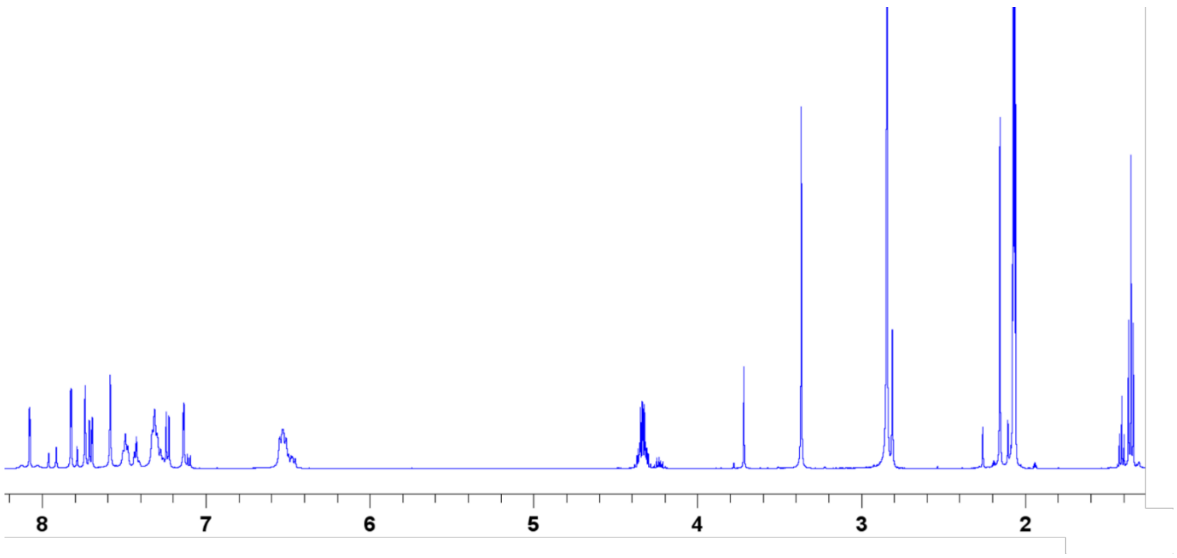

(b)

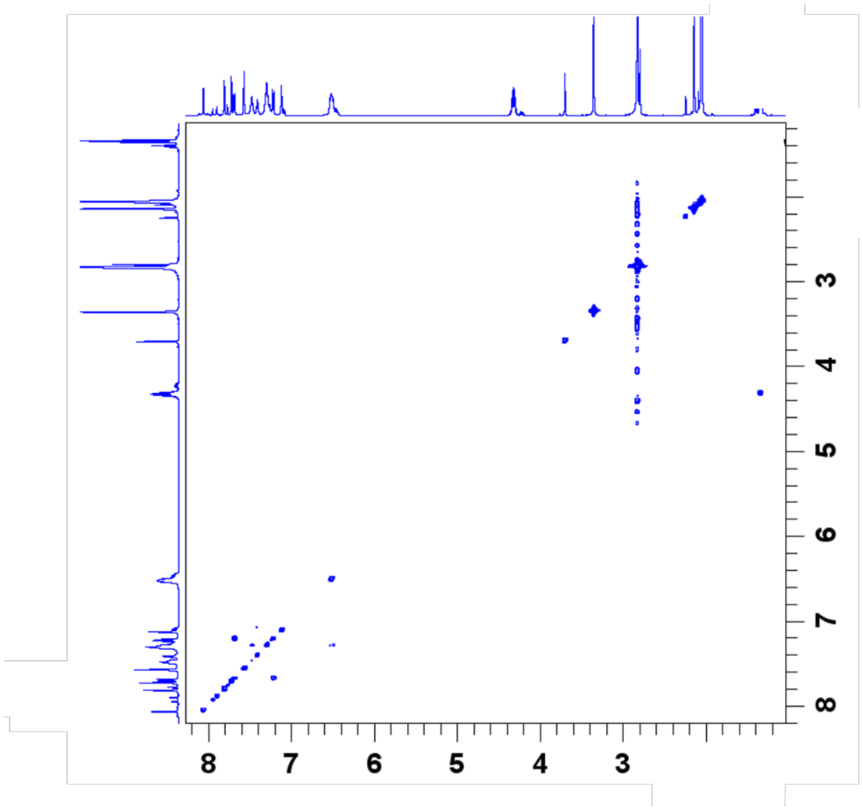

(c)

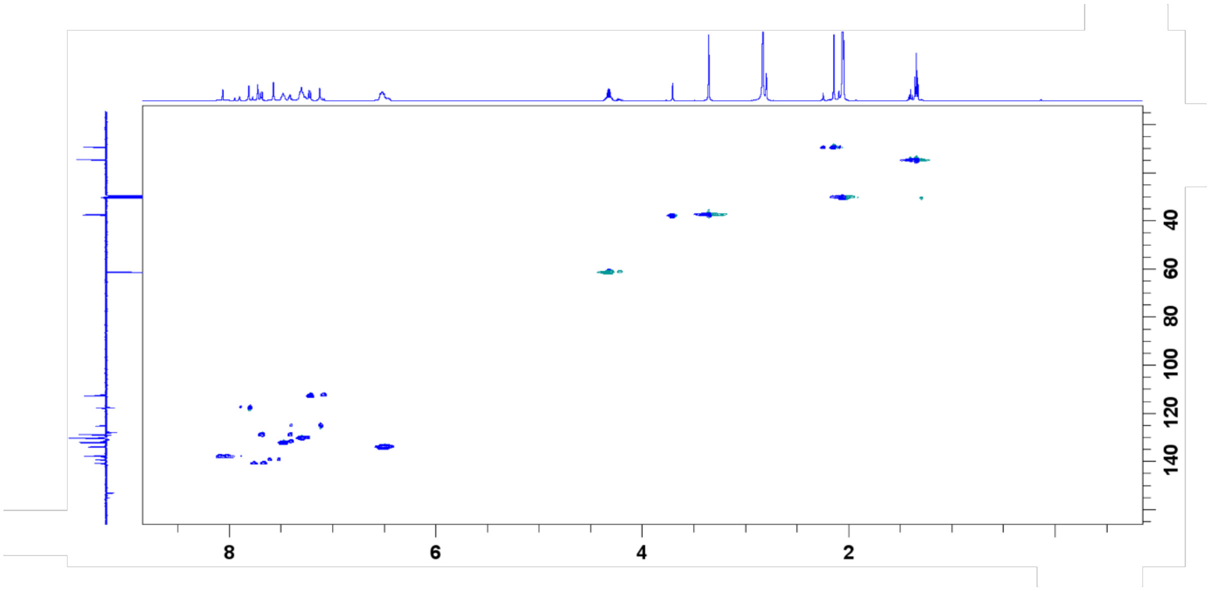

(d)

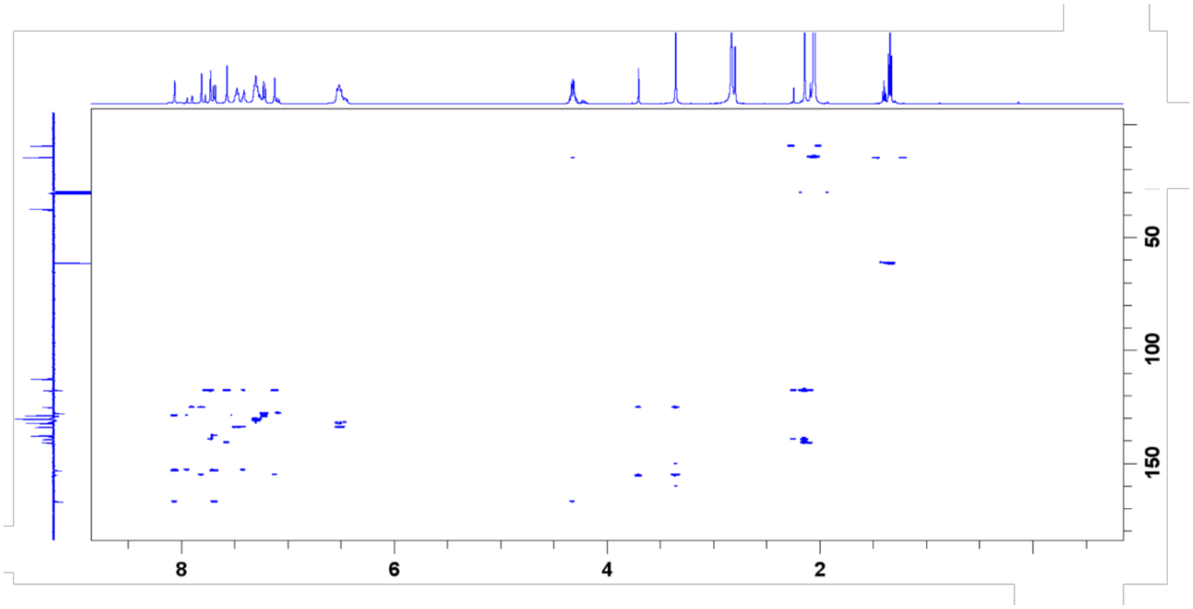

.

(e)

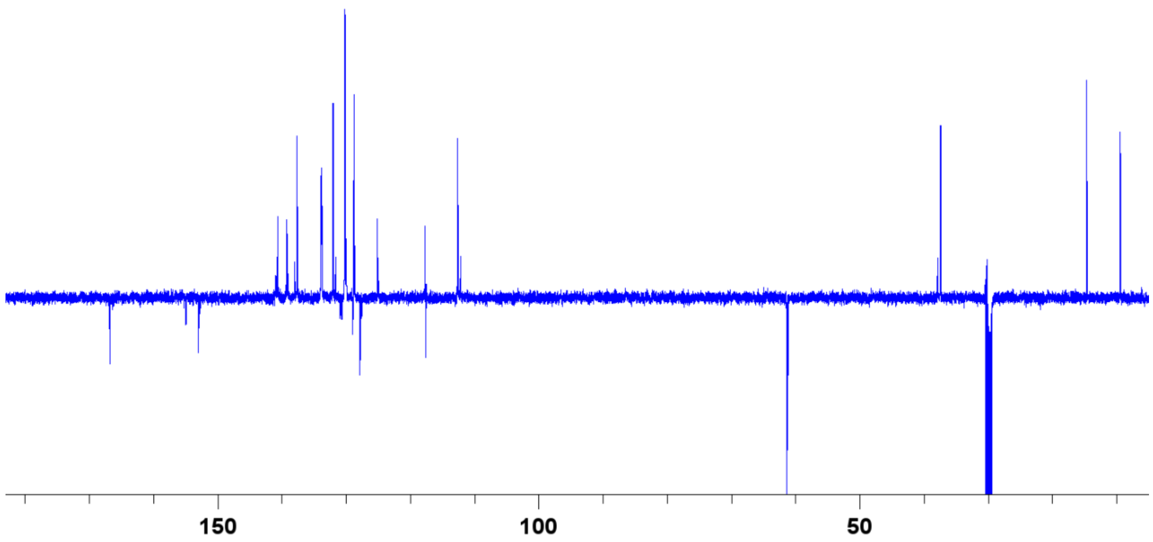

(f)

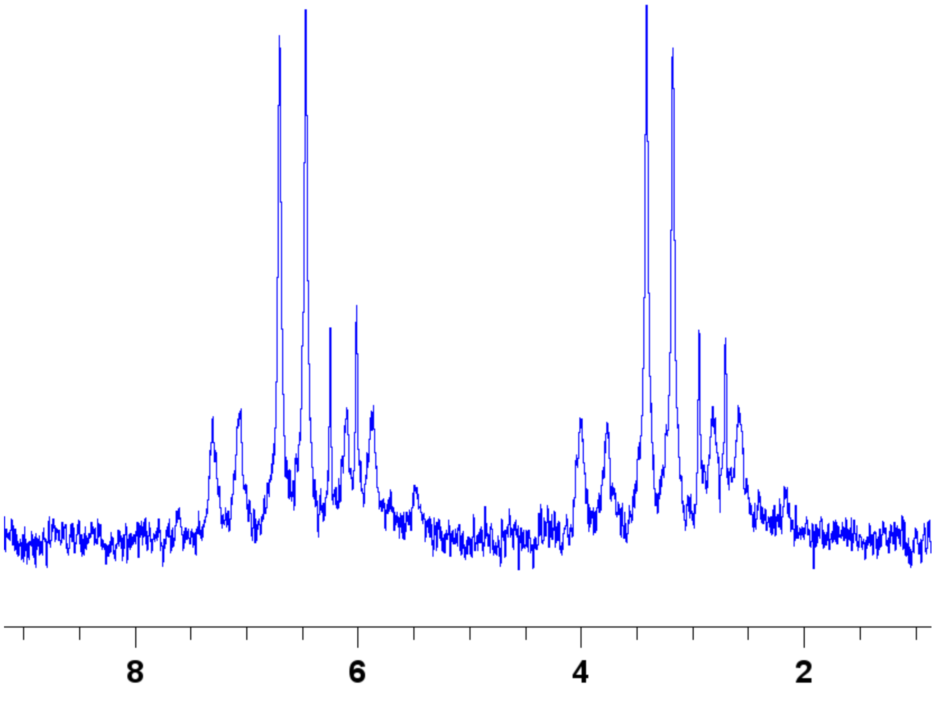

(g)

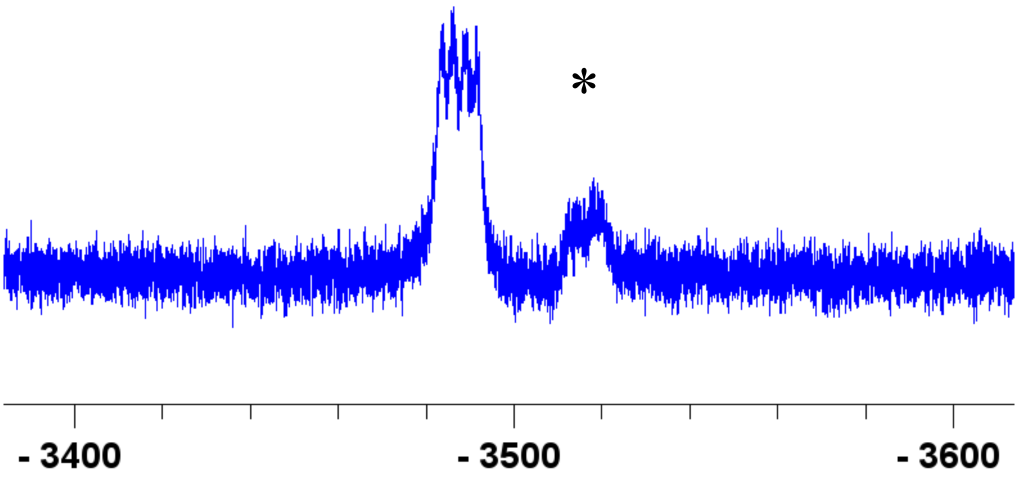
*
- 3400
- 3500
- 3600

(h)

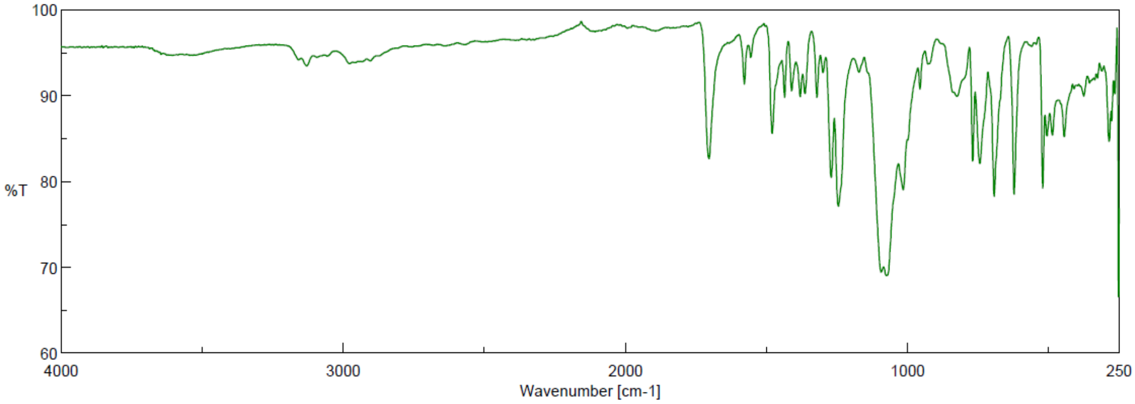
100
90
80
70
60
%T
4000
3000
2000
1000
250
Wavenumber [cm-1]

(i)

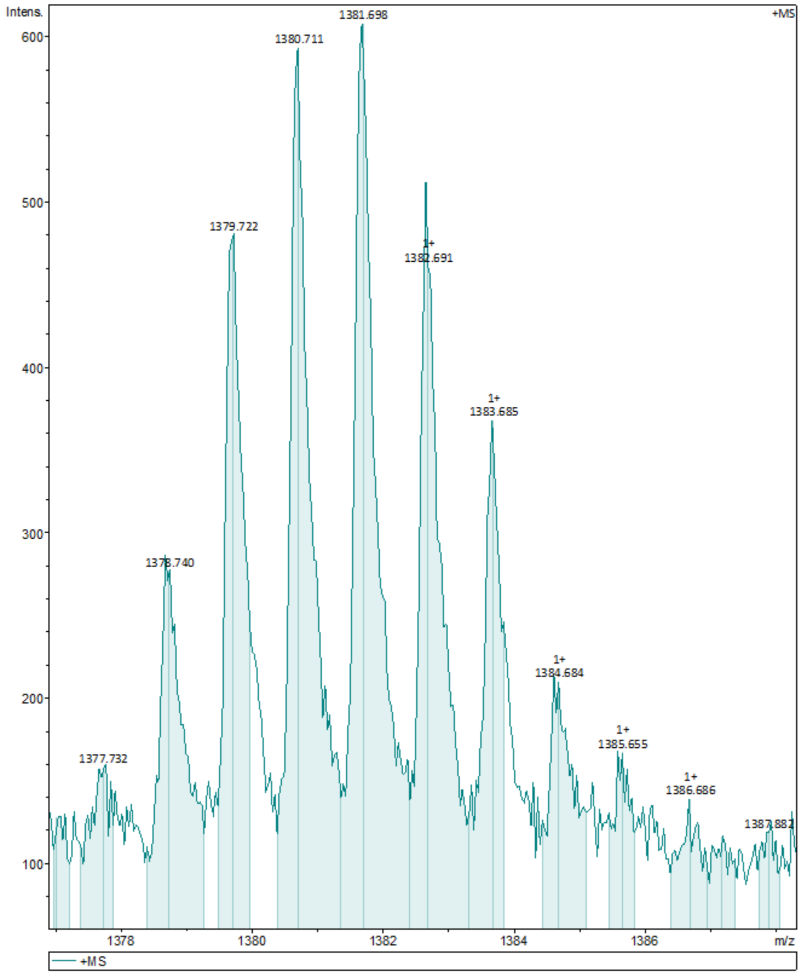

Figure S2: a. ^{1}H NMR spectrum of **1b** in acetone- d_6. **b.** ^{1}H-^{1}H COSY NMR spectrum of **1b** in acetone- d_6. **c.** ^{1}H-^{13}C HSQC NMR spectrum of **1b** in acetone- d_6. **d.** ^{1}H-^{13}C HMBC NMR spectrum of **1b** in acetone- d_6. **e.** ^{13}C{^{1}H} APT NMR spectrum of **1b** in acetone- d_6. **f.** ^{31}P{^{1}H} NMR spectrum of **1b** in acetone- d_6. **g.** ^{195}Pt{^{1}H} spectrum of **1b** at 193K NMR in acetone- d_6. * *syn* isomer. **h**. IR spectrum of **1b**. **i**. Mass spectrum of **1b**.

(a)

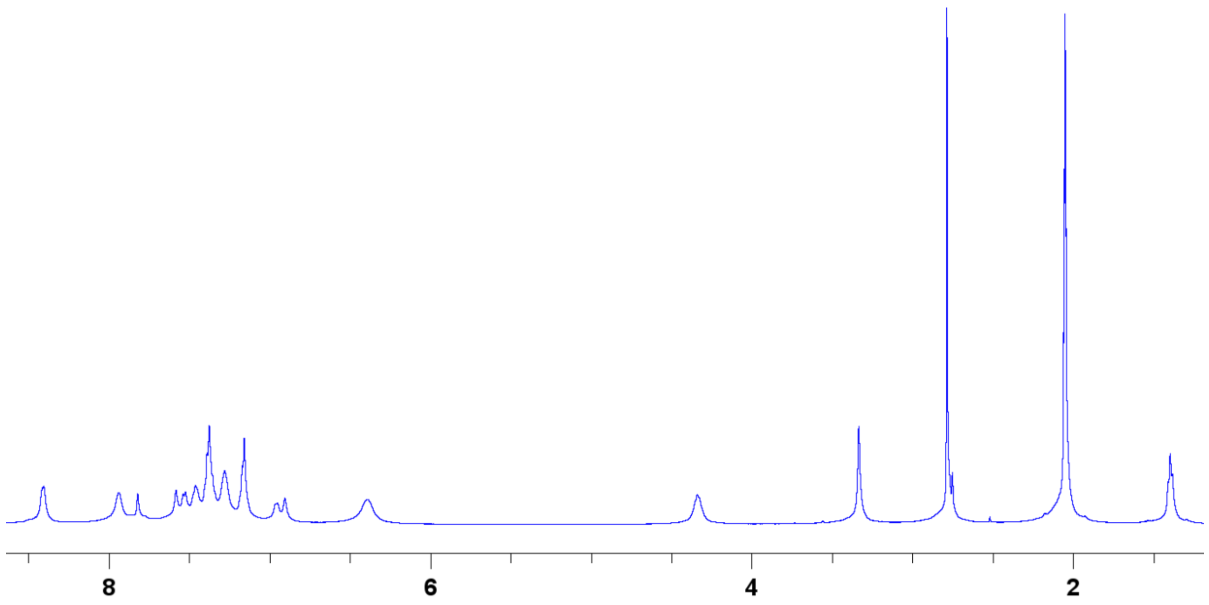

(b)

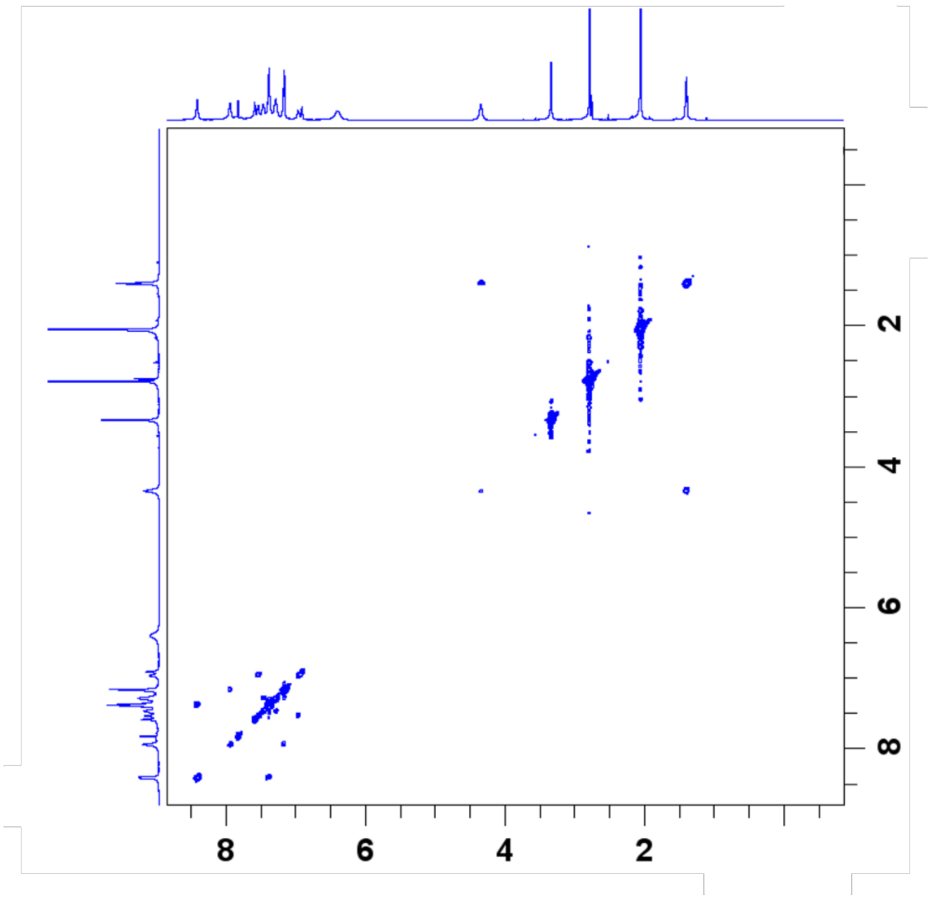

(c)

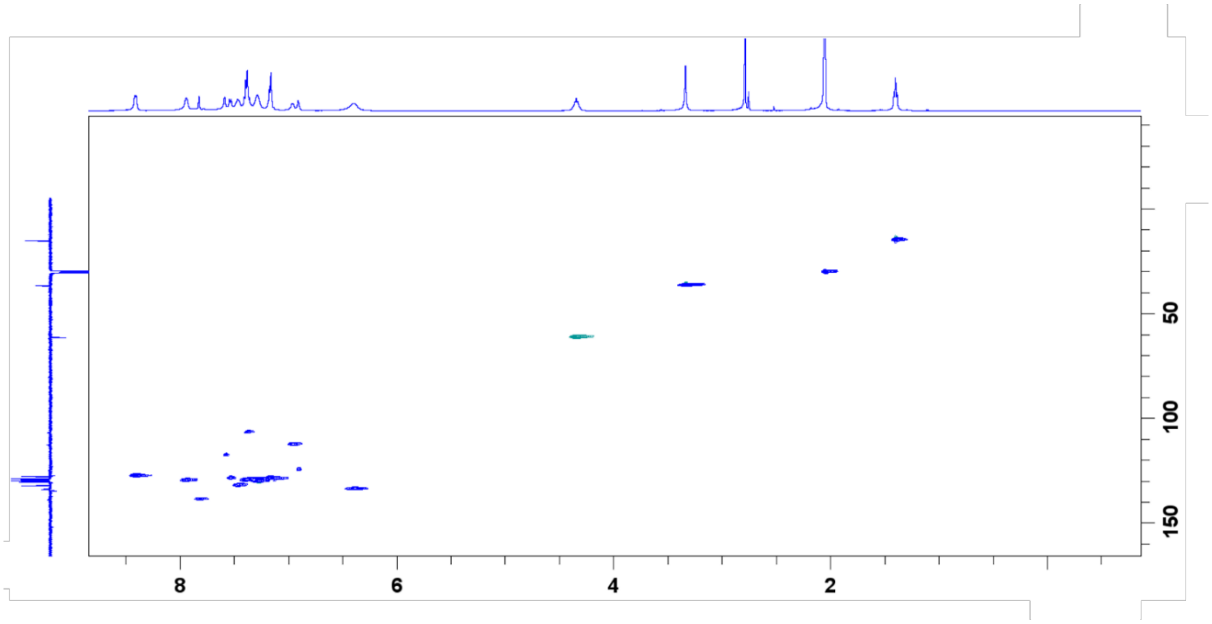

(d)

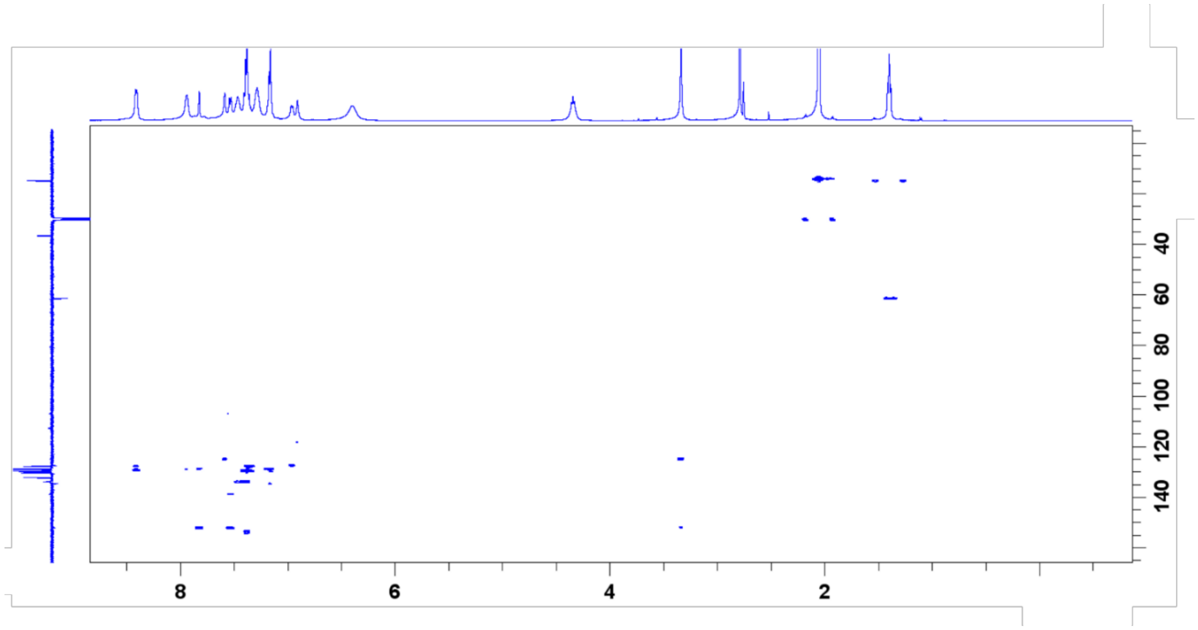

(e)

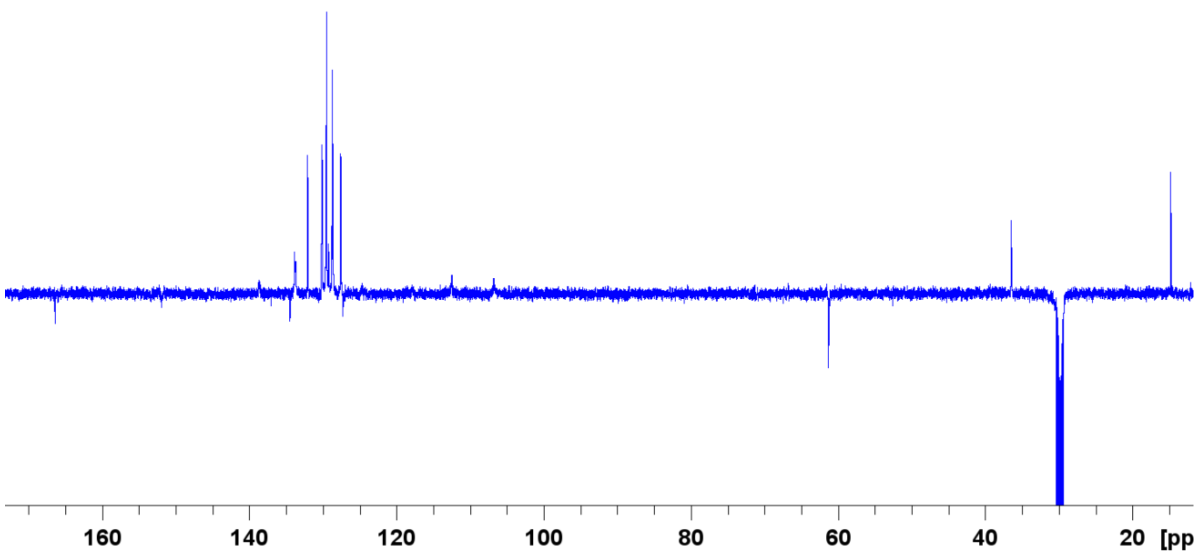

(f)

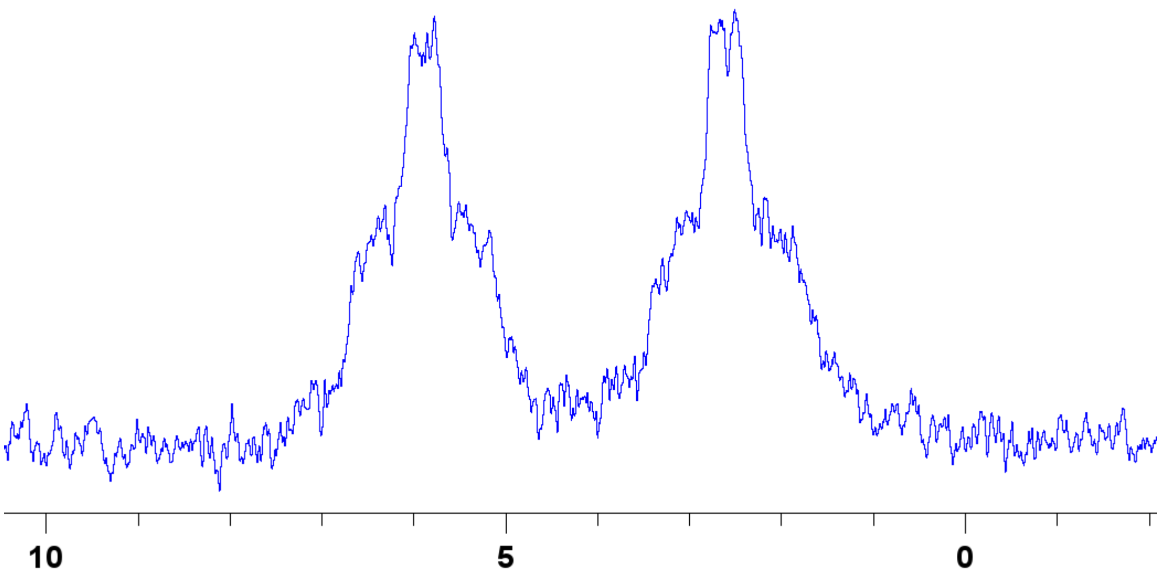

(g)

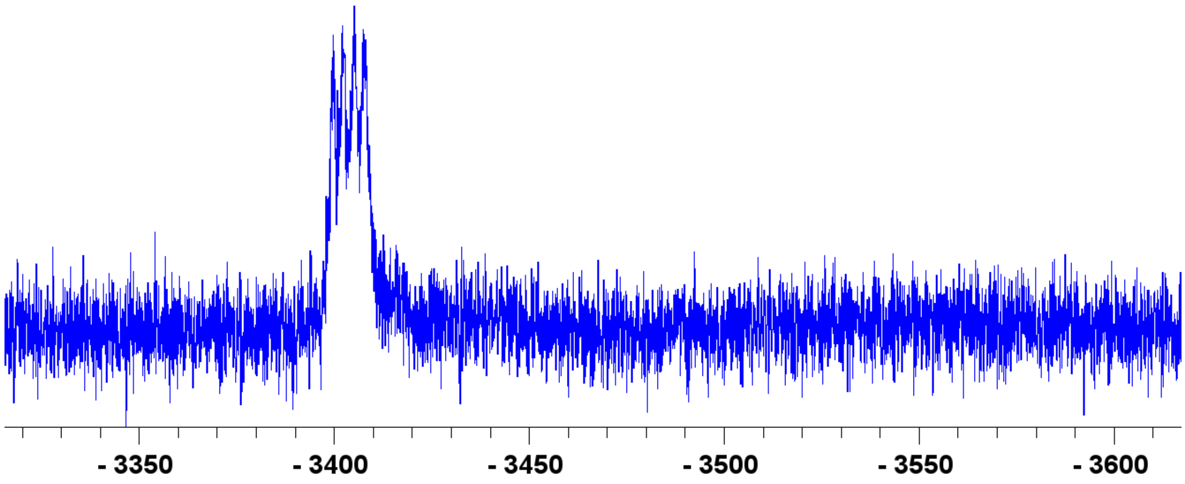
- 3350
- 3400
- 3450
- 3500
- 3550
- 3600

(h).

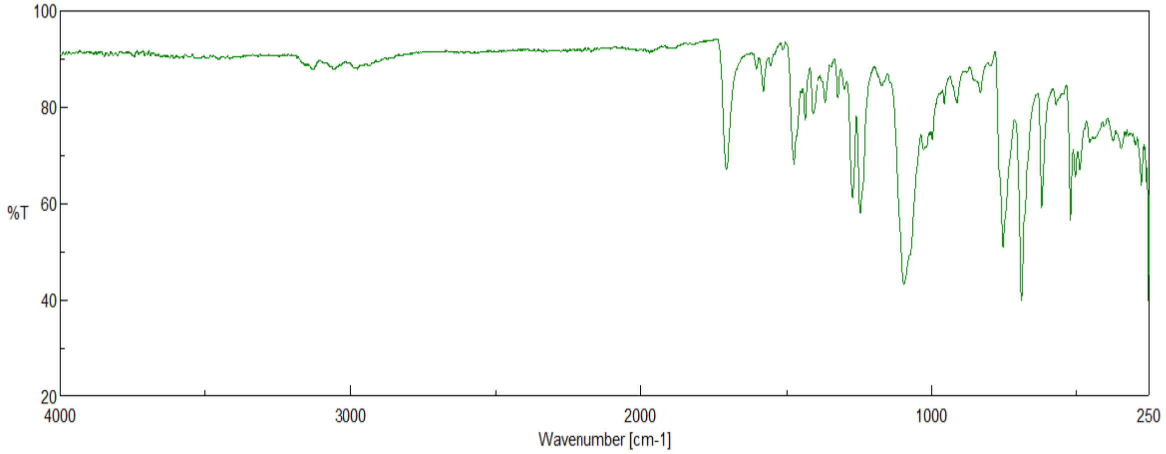
100
80
60
40
20
%T
4000
3000
2000
1000
250
Wavenumber [cm-1]

(i)

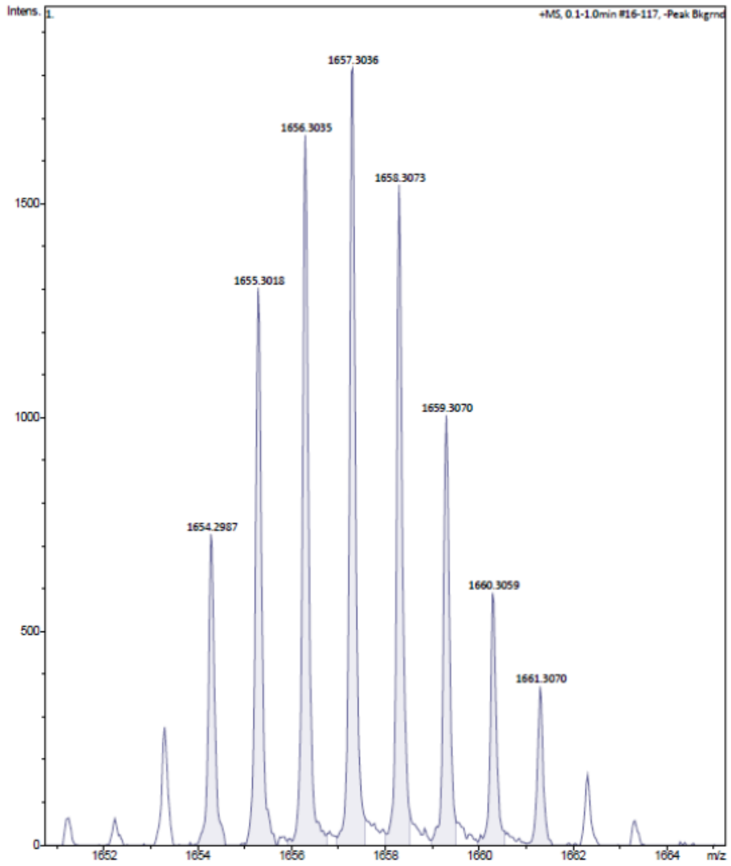

Figure S3: a. ^{1}H NMR spectrum of **1c** in acetone- d_6. **b.** ^{1}H-^{1}H COSY NMR spectrum of **1c** in acetone- d_6. **c.** ^{1}H-^{13}C HSQC NMR spectrum of **1c** in acetone- d_6. **d.** ^{1}H-^{13}C HMBC NMR spectrum of **1c** in acetone- d_6. **e.** ^{13}C{^{1}H} APT NMR spectrum of **1c** in acetone- d_6. **f.** ^{31}P{^{1}H} NMR spectrum of **1c** in acetone- d_6. **g.** [195]Pt{^{1}H} NMR spectrum of **1c** at 193K in acetone-d_6. **h**. IR spectrum of **1c. i**. Mass spectrum of **1c**.

(a)

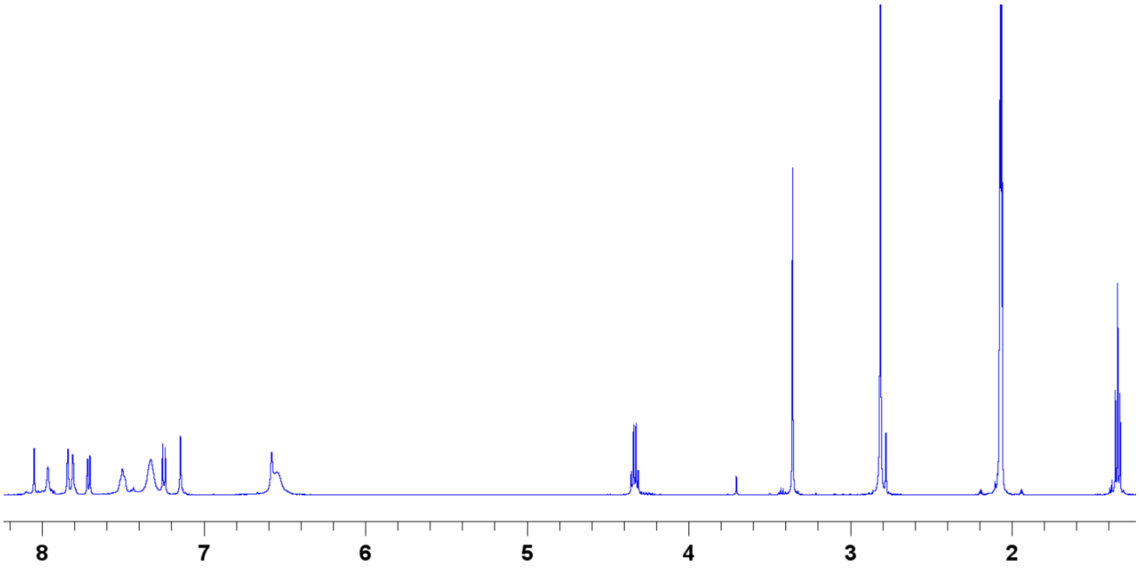

(b)

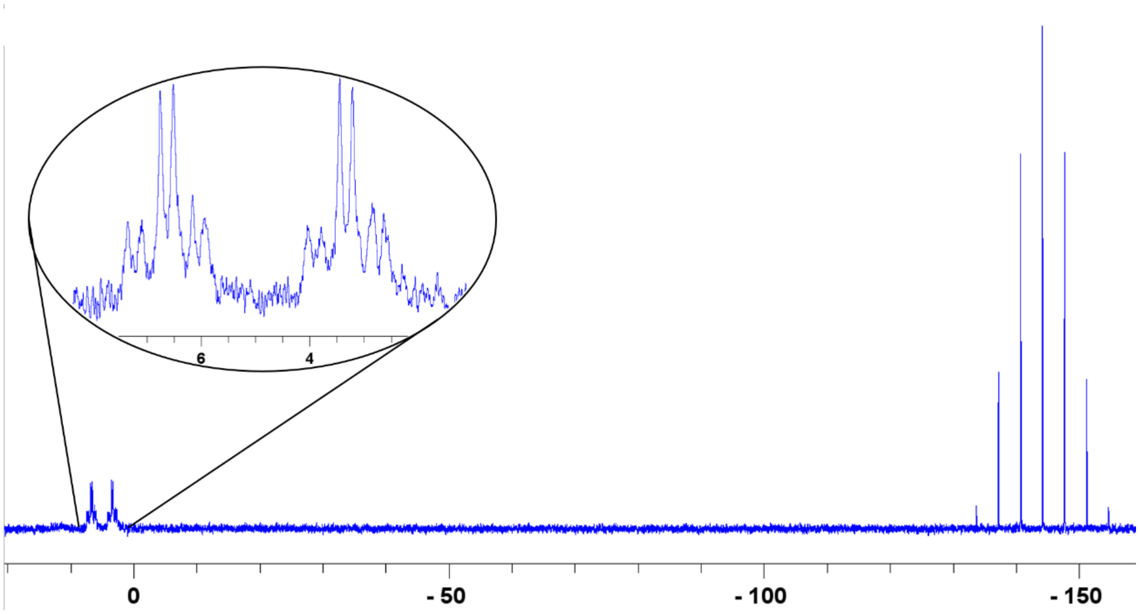

(c)

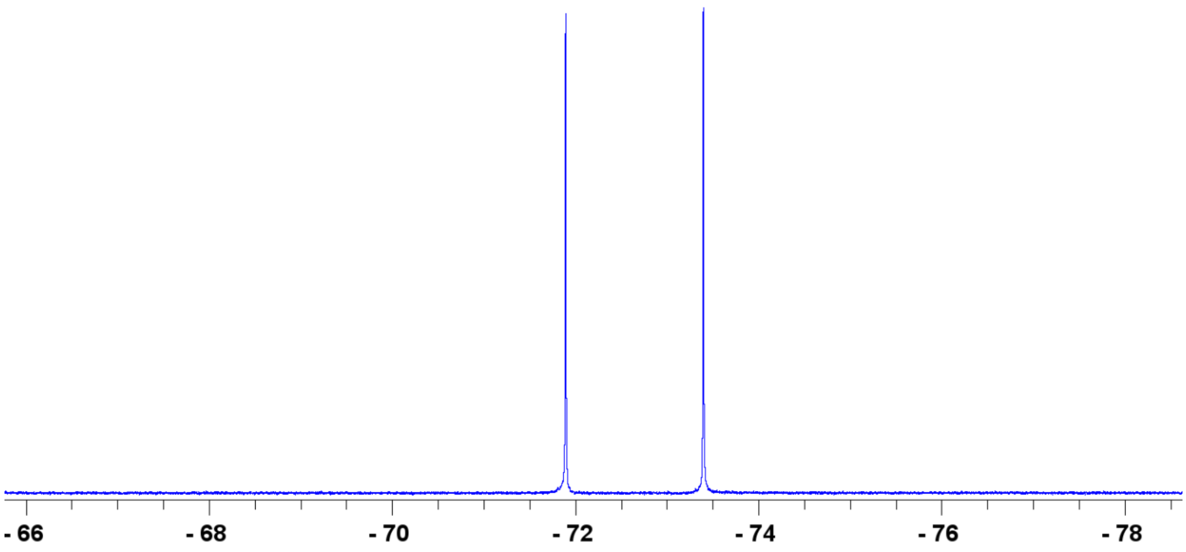

(d)

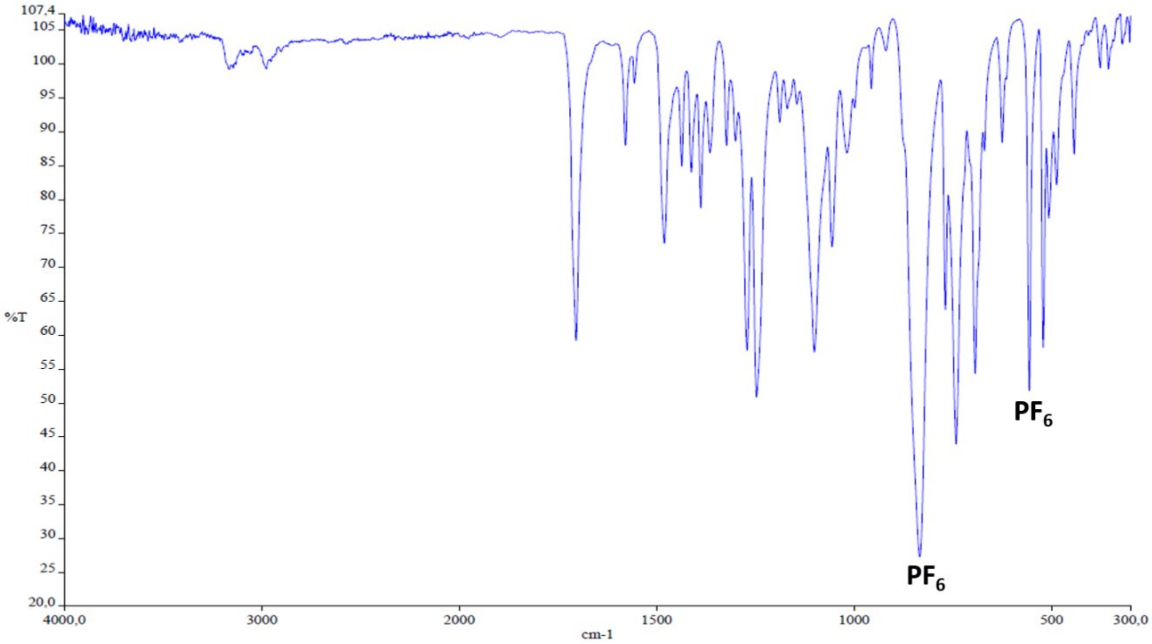

Figure S4: a. ^{1}H NMR spectrum of **2a** in acetone- d_6. **b.** ^{31}P{^{1}H} NMR spectrum of **2a** in acetone- d_6. **c.** ^{19}F{^{1}H} NMR spectrum of **2a** in acetone- d_6. **d.** IR spectrum of **2a**.

(a)

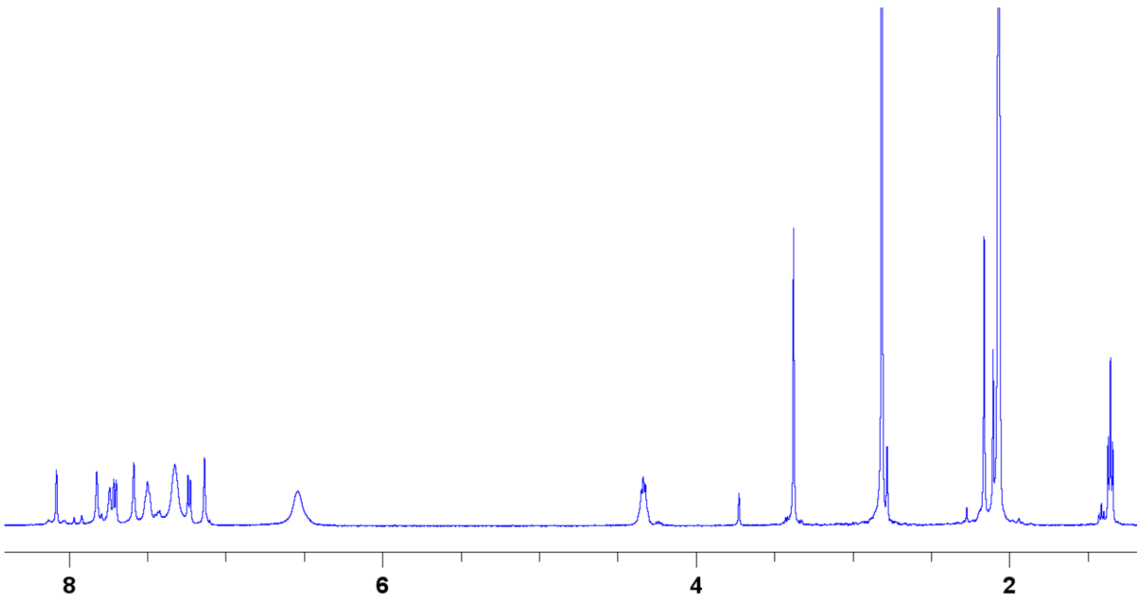

(b)

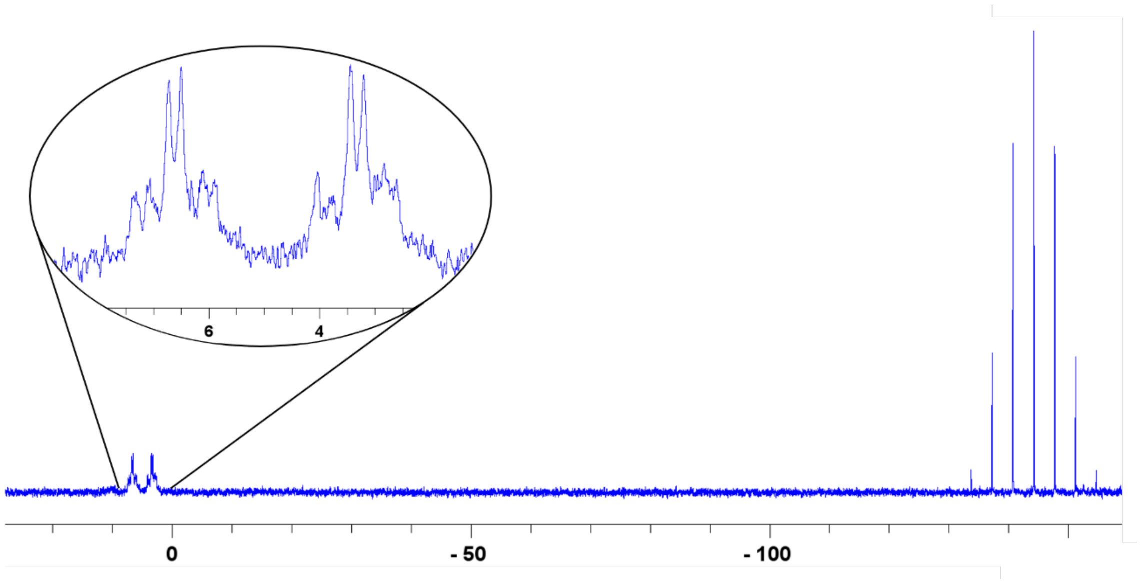

(c)

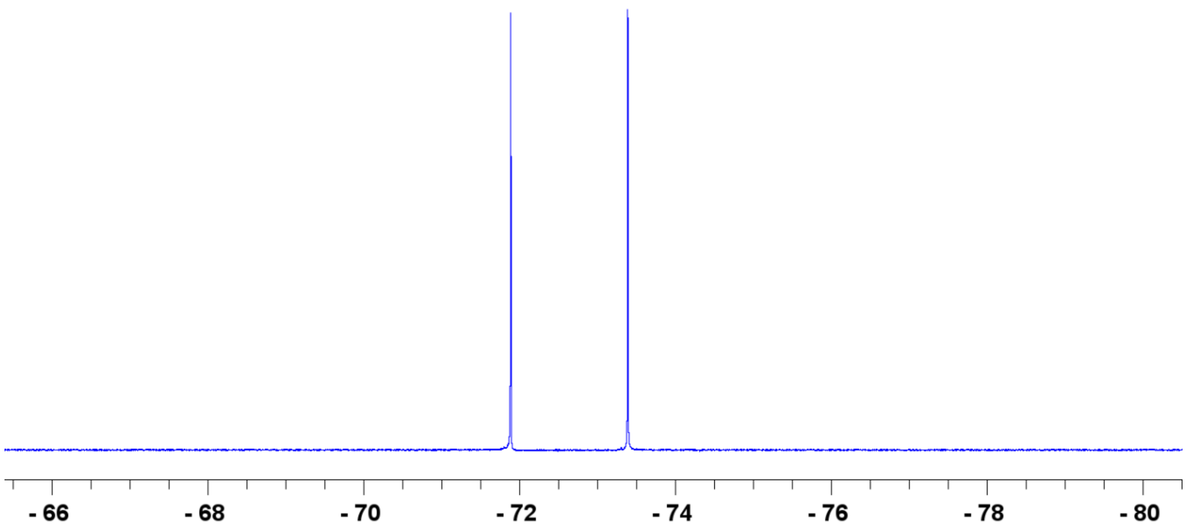

(d)

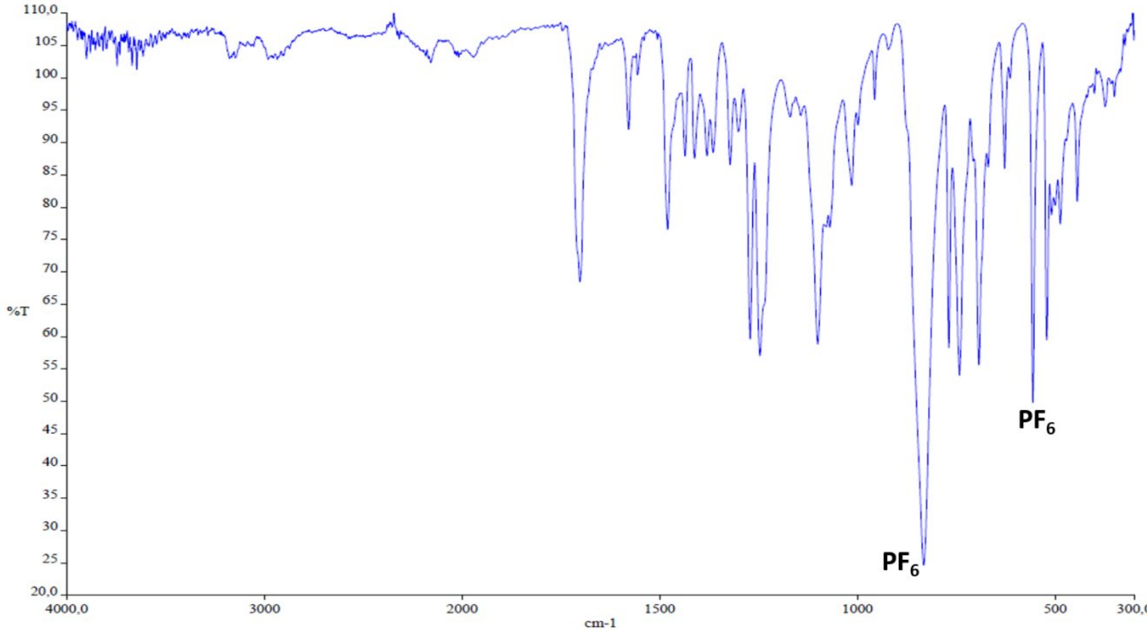

Figure S5: a. ^{1}H NMR spectrum of **2b** in acetone- d_6. **b.** $^{31}P\{^{1}H\}$ NMR spectrum of **2b** in acetone- d_6. **c.** $^{19}F\{^{1}H\}$ NMR spectrum of **2b** in acetone- d_6. **d.** IR spectrum of **2b**.

(a)

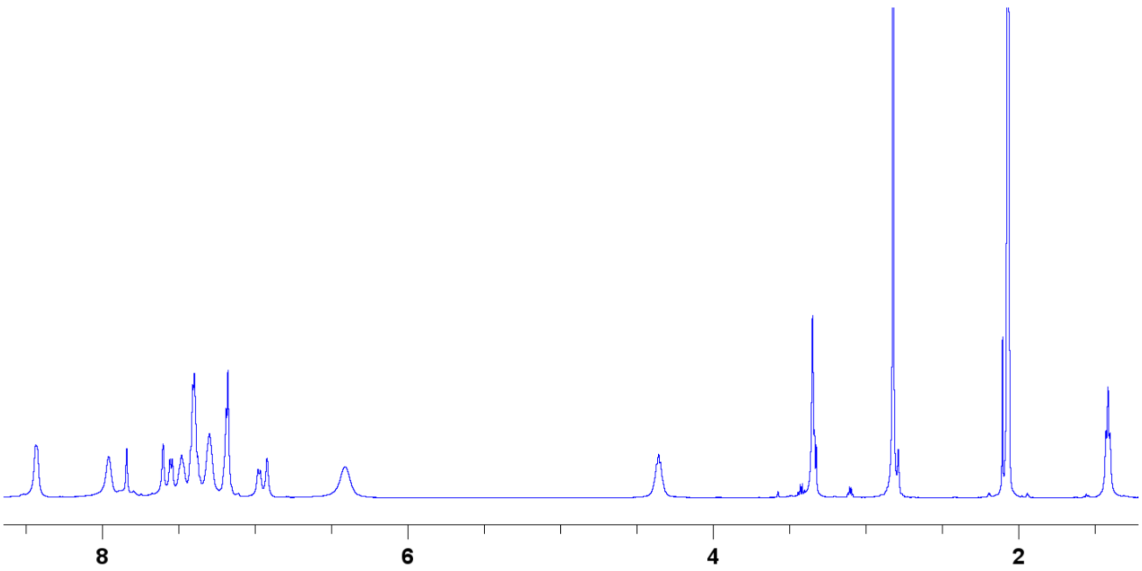

(b)

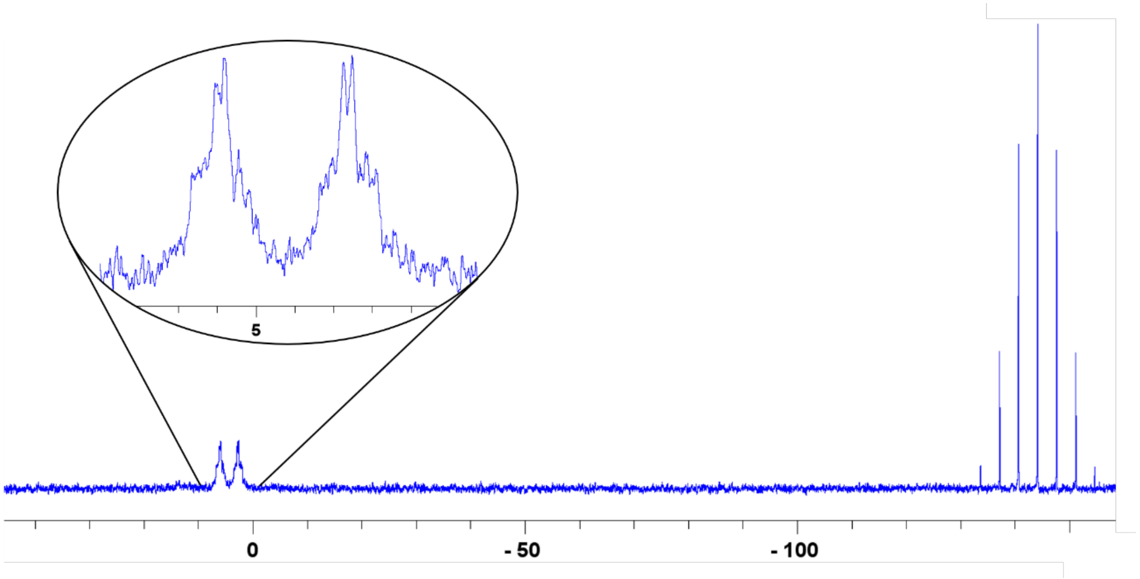

(c)

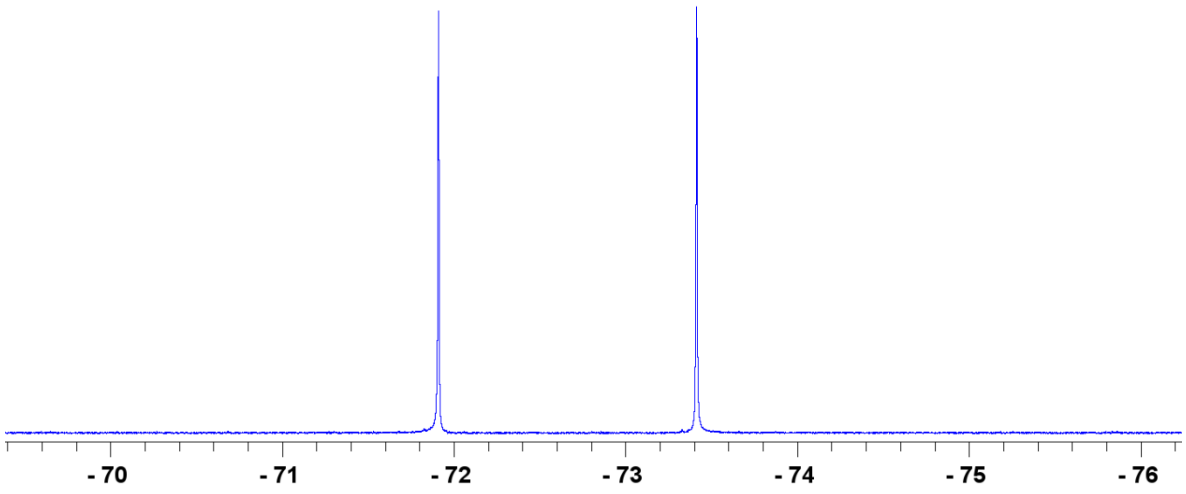

(d)

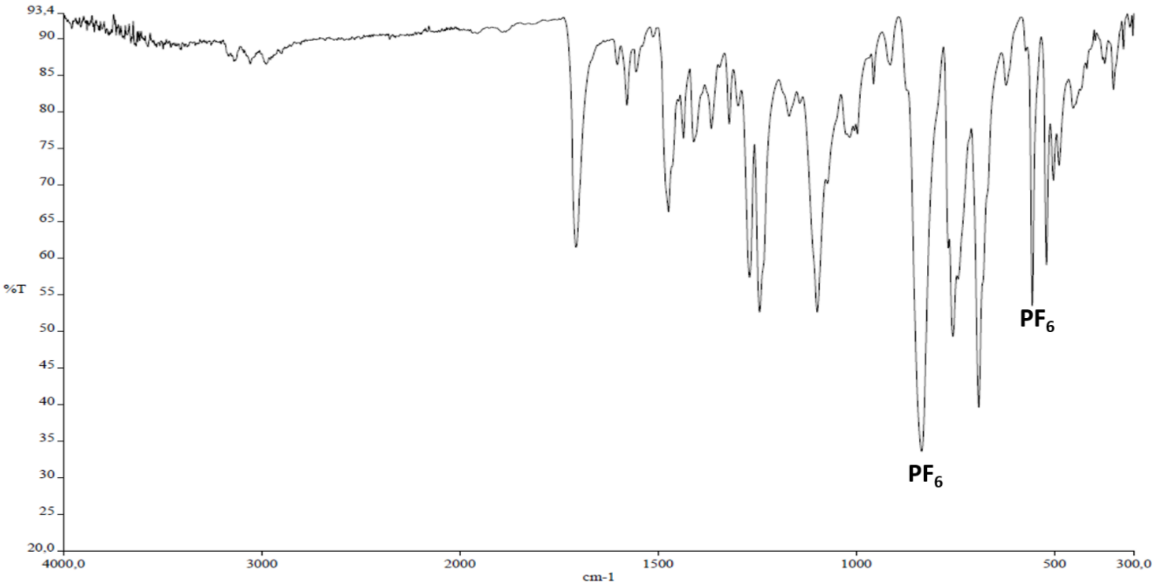

Figure S6: a. ^{1}H NMR spectrum of **2c** in acetone- d_6. **b.** $^{31}P\{^{1}H\}$ NMR spectrum of **2c** in acetone- d_6. **c.** $^{19}F\{^{1}H\}$ NMR spectrum of **2c** in acetone- d_6. **d.** IR spectrum of **2c**.

3. Full description of the single crystal X-ray structures of compounds 1a-1c.

Table S2 shows a selection of bond distances and angles for **1a, 1b** and **1c**, whose molecular structures appear in the Manuscript.

These structures confirmed the trinuclear [Pt_2Ag] nature of the complex units, with the silver atom linked to the two platinum centers of the corresponding starting material "[{Pt(C^C*)(*μ*-Rpz)}$_2$]". Then, the Ag center completes its coordination environment with different L ligands. In the four complexes, the platinum fragments are comprised of two "Pt(C^C*)" metallacycles bridged by two Rpz ligands (pz, **1a**, 4-Mepz **1b**, 3,5-dppz **1c**) displaying an *anti*-arrangement of the C^C* groups.

The six-membered ring Pt_2N_4 has the typical boat-like conformation with an angle between the Pt-N-N-Pt fragments of about 80° (77.80° **1a**, 77.77° **1b,** 81.32° **1c**) and Pt···Pt separation (3.3907(6) Å, **1a**, 3.3526(8) Å **1b**, 3.1761(4) Å **1c**) large enough to preclude significant intermetallic interaction. The dihedral angle between the best least-squares planes of the platinum environments is 85.12° in **1a**, 83.84° in **1b** and 73.76° in **1c**. This disposition creates an appropriate site for the silver atom to be located. Therefore, the Ag center bonds to the "[{Pt(C^C*)(*μ*-Rpz)}$_2$]" fragment through two Pt-Ag donor-acceptor bonds. The Pt-Ag bond distances, of about 2.75 Å (see Table S2), are within the range found for this kind of bonds.[15,16] The Pt-Ag vectors are roughly perpendicular to the coordination planes of their respective Pt atoms, in **1a** and **1b** with the angles being close to 10° (9.70° (Pt1) and 10.76° (Pt2) in **1a**; 15.74° (Pt1) and 13.18° (Pt2) in **1b**) but deviate from them in **1c** (19.34° (Pt1) and 17.35° (Pt2)). This structural parameter is indicative of a better orbital overlap to form the Pt-Ag bonds in the pz and 4-Mepz derivatives.[17]

The Pt(1)-Ag-Pt(2) angle is ca 70.0°, similar to those found in other complexes in which a dinuclear Pt complex acts as a chelating ligand toward silver. For instance in [{Pt($CH_2C_6H_4$P(o-tolyl)$_2$-*κ*C,P)(*μ*-4-Mepz)}$_2$Ag]ClO_4 is 75.28(2)°,[15] in [Pt_2Ag(μ-Cl)$_2$(C_6F_5)$_4$(OEt_2)]$^-$ is 72.15°[18] and in [(C_6F_5)$_2$Pt(*μ*-OH)(*μ*-dppm){Ag(PPh_3)}Pt(C_6F_5)$_2$]$^-$ is 76.4°.[19] In the three derivatives **1a**-**1c**, the Ag center completes its coordination sphere with a PPh_3 ligand, showing a highly distorted triangular coordination environment.

Table S2. Selected bond distances (Å) and angles (°) for **1a**, **1b**, **1c**.

	**1a**	**1b**	**1c**
Pt(1)-Ag(1)	2.7844(5)	2.7628(12)	2.8012(7)
Pt(1)-C(1)	1.984(6)	1.967(16)	1.980(8)
Pt(1)-C(6)	2.013(5)	2.007(15)	2.013(7)
Pt(1)-N(7)	2.055(5)	2.063(10)	2.059(6)
Pt(1)-N(5)	2.087(4)	2.072(11)	2.109(5)
Pt(2)-Ag(1)	2.7396(5)	2.7448(11)	2.7606(6)
Pt(2)-C(14)	1.978(5)	1.970(13)	1.981(7)
Pt(2)-C(19)	2.018(5)	2.012(11)	2.000(8)
Pt(2)-N(6)	2.062(4)	2.038(11)	2.081(6)
Pt(2)-N(8)	2.102(4)	2.072(9)	2.117(6)
Ag(1)-P(1)	2.397(2)	2.398(4)	2.396(2)
C(1)-Pt(1)-C(6)	80.2(2)°	79.6(6)°	79.5(3)
C(1)-Pt(1)-N(5)	99.7(2)°	101.2(6)°	100.7(3)
C(6)-Pt(1)-N(7)	95.2(2)°	94.6(5)°	94.4(3)
N(7)-Pt(1)-N(5)	84.86(18)°	84.7(4)°	85.3(2)
C(14)-Pt(2)-C(19)	80.4(2)°	79.1(5)°	79.9(3)
C(14)-Pt(2)-N(8)	99.6(2)°	100.1(4)°	98.8(3)
C(19)-Pt(2)-N(6)	93.9(2)°	93.5(5)°	93.9(3)
N(6)-Pt(2)-N(8)	86.19(17)°	87.2(4)°	87.4(2)
Pt(2)-Ag(1)-Pt(1)	75.73(1)°	74.99(3)°	69.64(2)
P(1)-Ag(1)-Pt(1)	131.62(4)°	140.97(10)°	142.81(6)
P(1)-Ag(1)-Pt(2)	143.80(4)°	142.04(10)°	141.21(5)

4. Absorption data and theoretical calculations for the $[Pt_2Ag(PPh_3)]^+$ clusters.

Table S3. Absorption data for **1a-c** in solution of 2-MeTHF at 298 K.

Comp.	λ_{abs} /nm (10^3 ε M^{-1} cm^{-1})
**1a**[a]	250 (42.35), 295 (14.97), 307_{sh} (12.73), 365 (13.62) tail to 465
**A**[b]	245 (45.82), 309 (11.28), 335 (11.84), 354_{sh} (9.03) tail to 425
**1b**[a]	249 (33.97), 295 (12.12), 309_{sh} (9.82), 342 (9.48), 360 (8.65) tail to 465
**B**[b]	241 (48.70), 249 (51.37), 310 (12.16), 338 (13.97), 359_{sh} (9.41) tail to 425
**1c**[a]	250 (41.71), 297 (27.96), 333_{sh} (7.45), 377 (8.06) tail to 450
**C**[c]	251 (108.8), 290_{sh} (43.5), 377 (6,7) tail to 450

[a] 10^{-4} M (pathlength 1cm); [b] 10^{-3} M (pathlength 1mm), [c] 10^{-5} M (pathlength 1cm). Data for the starting complexes, **A-C**, have been included for comparison.[25]

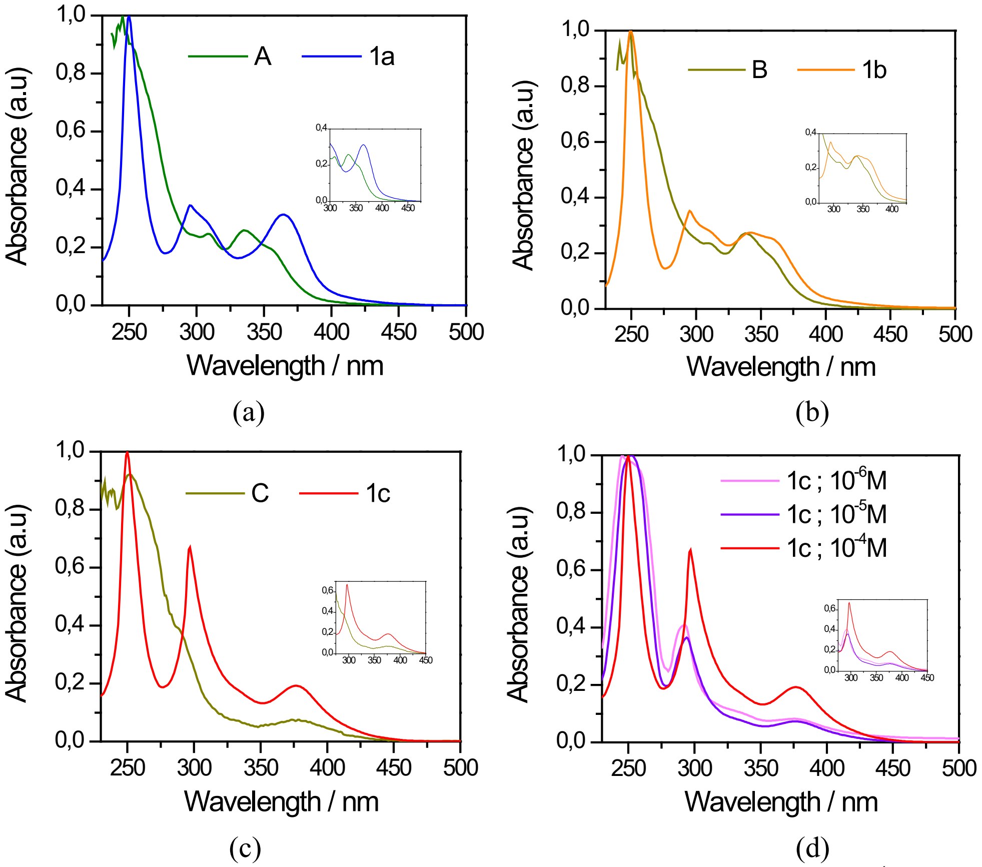

Figure S7. a-c: UV-visible spectra in 2-MeTHF of **1a**, **1b** and **1c** (10^{-4} M, pathlength: 1cm) with their corresponding starting materials **A**, **B** (10^{-3} M, pathlength: 1mm) and **C** (10^{-5} M, pathlength: 1cm). **d:** UV-visible spectra of **1c** in 2-MeTHF at different concentrations.

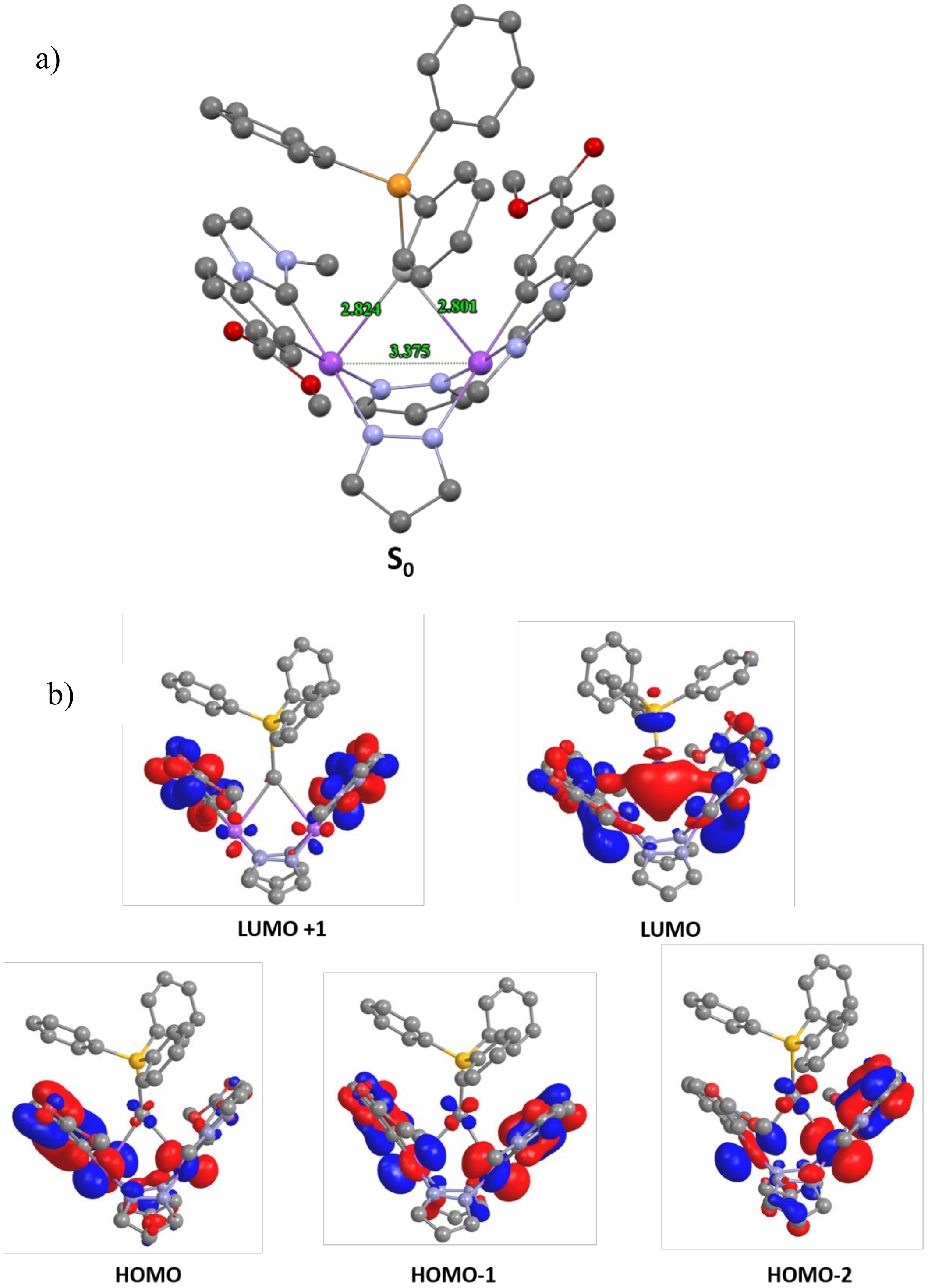

(b)

Figure S8. a) Optimized structure of S_o for **1a**. b) Calculated Frontier Molecular Orbital (isovalue =0.03) involved in the main calculated transition for **1a**.

Table S4. Population Analysis (%) of frontier MOs in the S_0 in THF for **1a**.

		Population Analysis				
	**eV**	**Pt**	**Ag**	**C^C***	**pz**	**PPh_3**
**LUMO+1**	-1.846	26	10	57	2	5
**LUMO**	-2.399	27	23	36	3	11
**HOMO**	-6.700	43	3	43	9	2
**HOMO-1**	-6.737	52	4	37	5	2
**HOMO-2**	-6.775	57	7	26	8	2

Table S5. Selection of the most significant and lowest-energy vertical singlet and triplet excitations calculated by TD-DFT for **1a** at the S_0 in solution of THF.

Transition	λ (nm)	o.s.	Transition (%)	Assignment
**S_3**	365.40	0.2149	H-2→L (62%), H-1→L (21%)<br>H→L (10%)	MM'CT/MLCT
**T_1**	452.64	0	H→L (45%), H→L+1 (15%)<br>H-1→L+1 (9%)	MM'CT/MLCT
**T_2**	449.75	0	H-1→L (33%), H-2→L (15%)<br>H-2→L+1 (10%), H-1→L+1 (8%)<br>HOMO→L+1 (5%)	MLCT/MM'CT

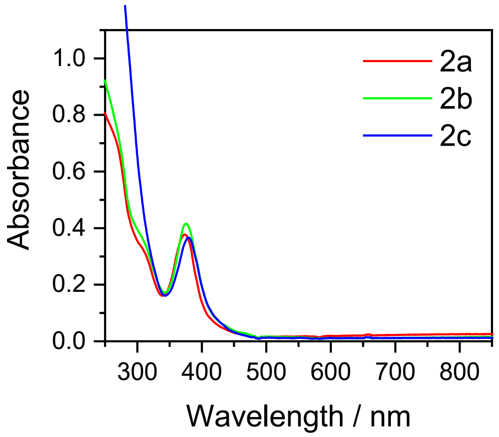

Figure S9. UV-visible absorption spectrum at r.t. of thin films of **2a-2c**.

5. Emission properties of the $[Pt_2Ag(PPh_3)]^+$ clusters.

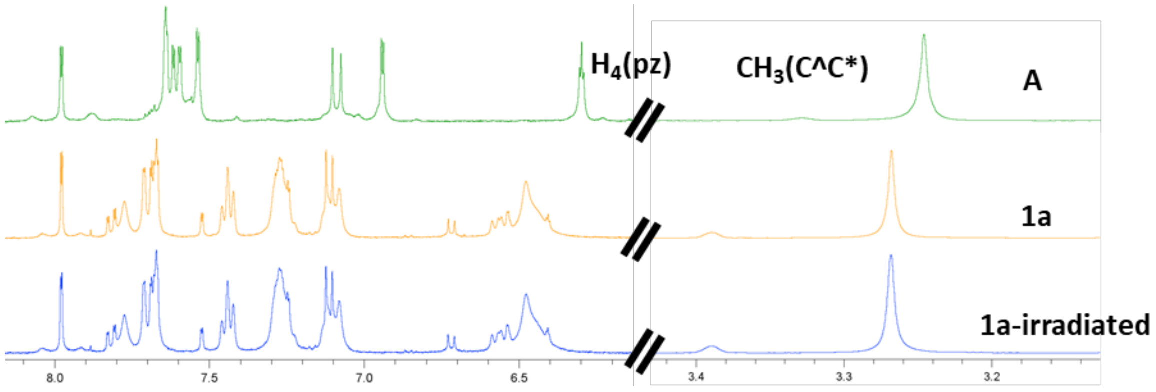

(a)

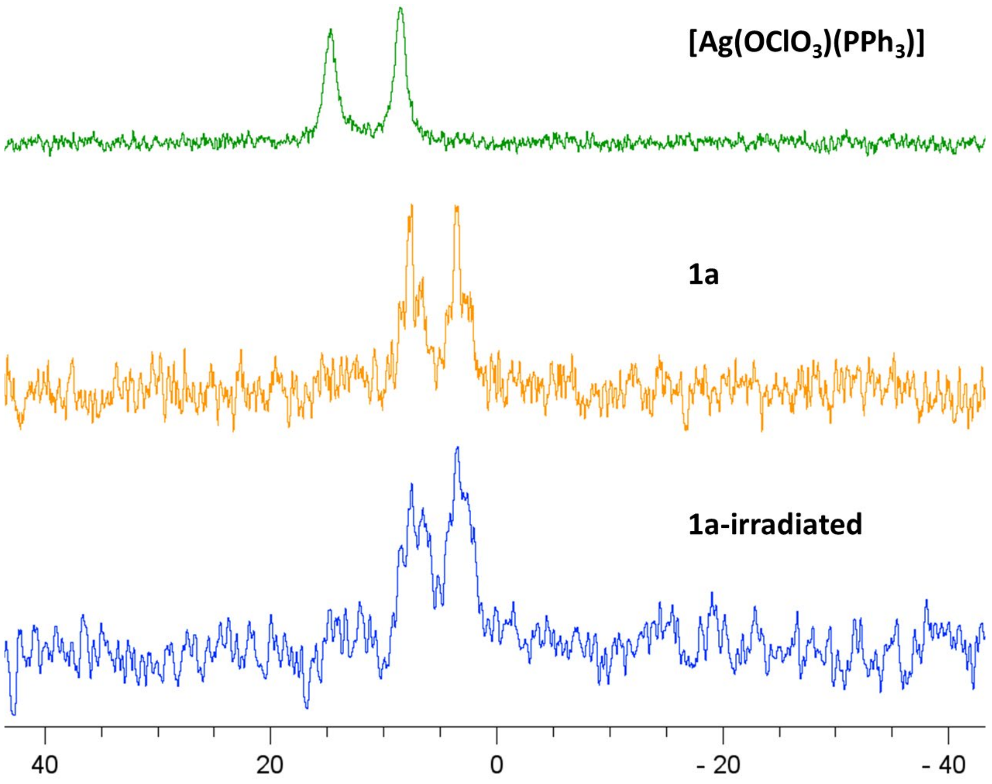

(b)

Figure S10. ^{1}H (a) and $^{31}P\{1H\}$ (b) NMR spectra of **1a** in THF-d_8 before and after irradiation with λ= 365 nm for 15 min. Spectra of the precursors have been included for comparison.

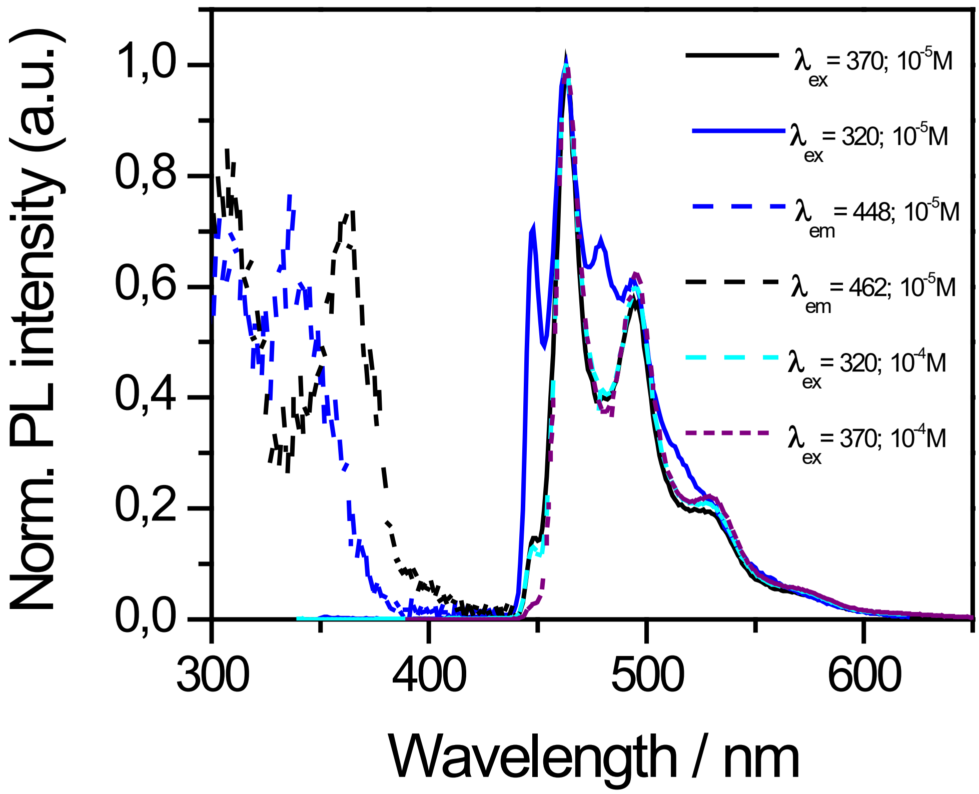
Norm. PL intensity (a.u.)
Wavelength / nm
λex = 370; 10-5M
λex = 320; 10-5M
λem = 448; 10-5M
λem = 462; 10-5M
λex = 320; 10-4M
λex = 370; 10-4M

(a)

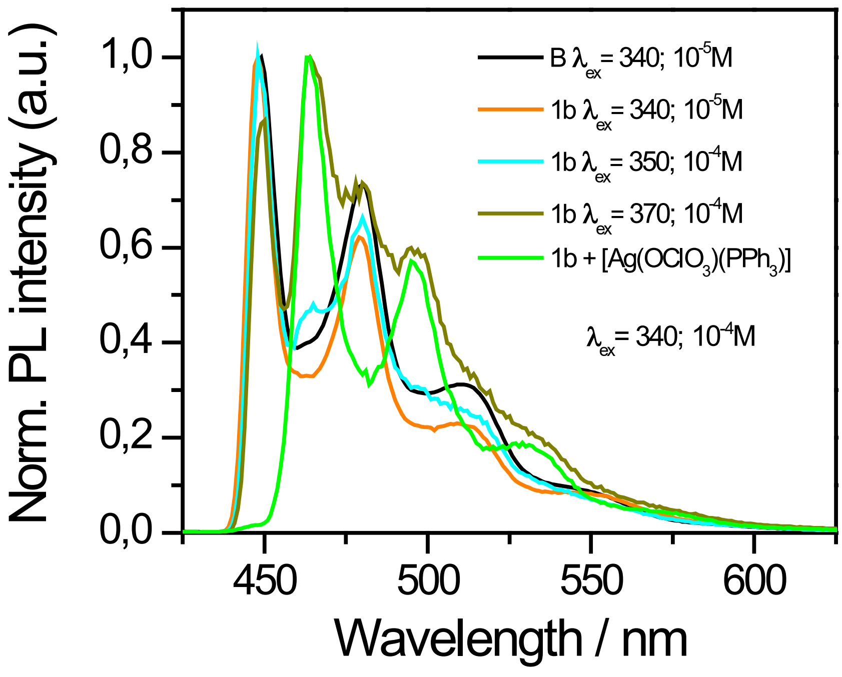
Norm. PL intensity (a.u.)
Wavelength / nm
B λex = 340; 10-5M
1b λex = 340; 10-5M
1b λex = 350; 10-4M
1b λex = 370; 10-4M
1b + [Ag(OClO3)(PPh3)]
λex = 340; 10-4M

(b)

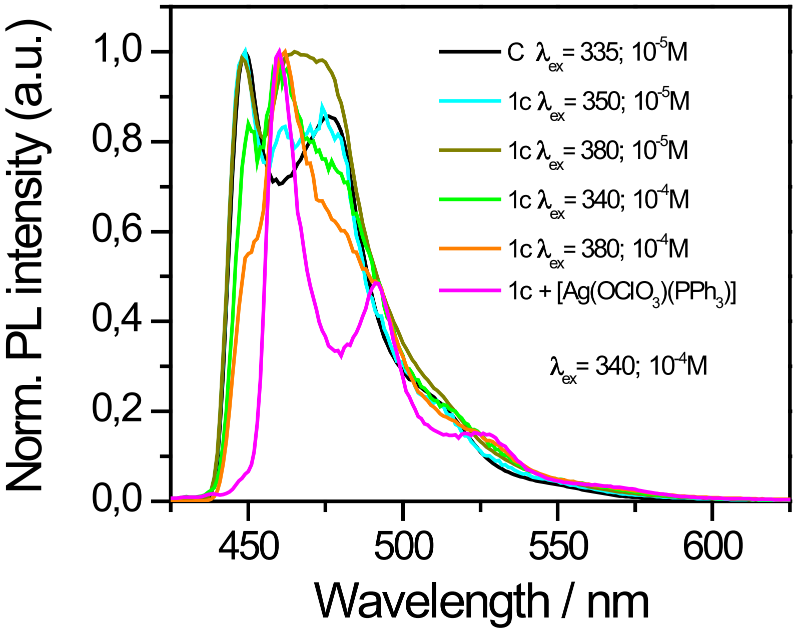

(c)

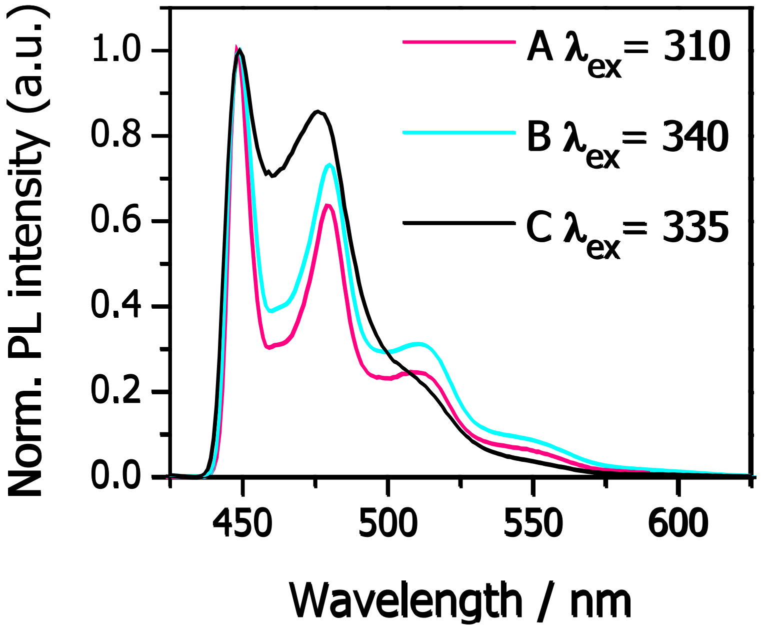

(d)

Figure S11. Normalized emission spectra of **1a (a) 1b (b)** and **1c (c)** in 2-MeTHF at 77K at different concentrations and excitation wavelengths, Normalized emission spectra of the precursors **B**-**C** in 2-MeTHF 10^{-5} M at 77 K (d).

6. Devices characterization and performances

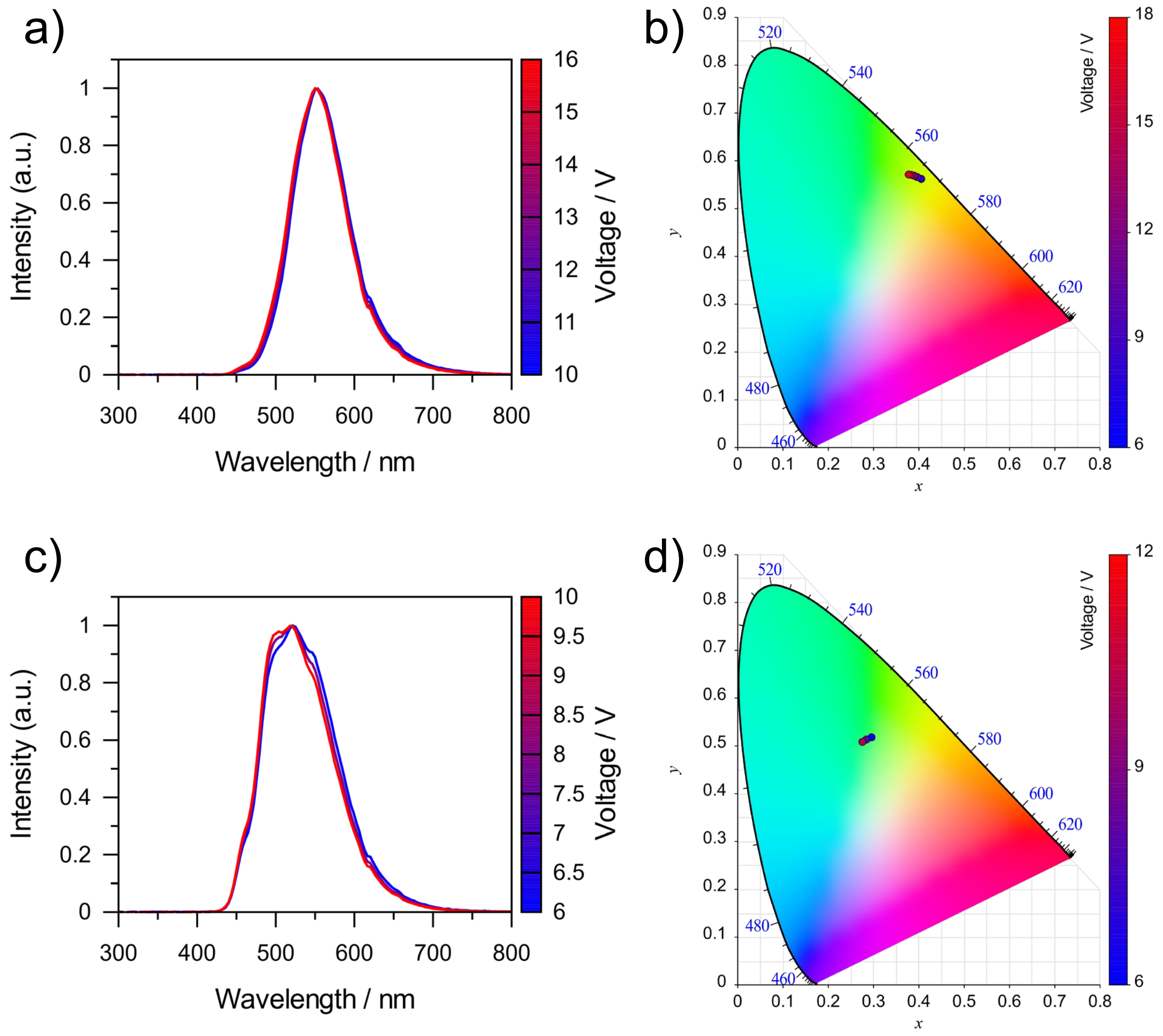

Figure S12. (a) Electroluminescence spectra of OLEDs employing thick (10 nm) **C** as the emitting material driven at different voltages (10-16 V). (b) CIE 1931 Chromaticity Diagram showing the same device's color points at the same voltage values. (c) and (d) EL spectra and Chromaticity Diagram of OLEDs employing ultrathin (1 nm) **C** as the emitting material driven at different voltages (6-10 V).

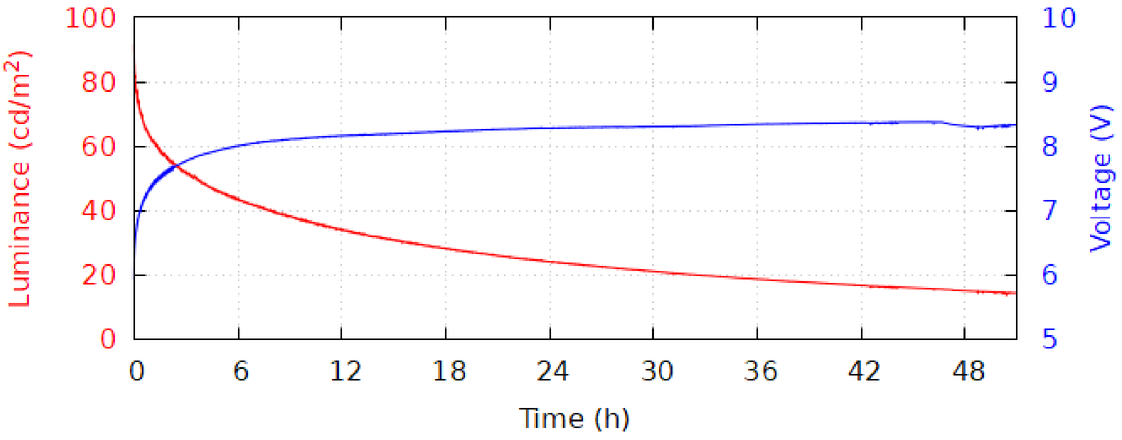

Figure S13. Evolution of luminance and driving voltage of OLEDs having an ultrathin (1 nm) **C** layer as emitting layer operated with a constant current density of 8 A m^{-2} over time.

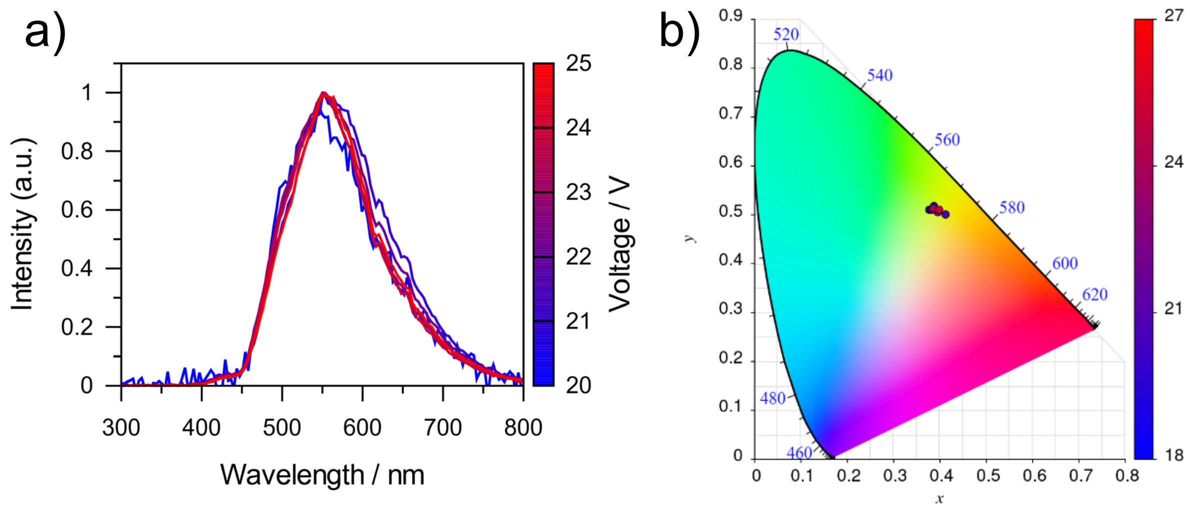

Figure S14. (a) Electroluminescence spectra of OLEDs employing **2c** as the emitting material driven at different voltage values, from 20 V to 25 V. (b) CIE 1931 Chromaticity Diagram showing the corresponding device's color points at the same voltage values.

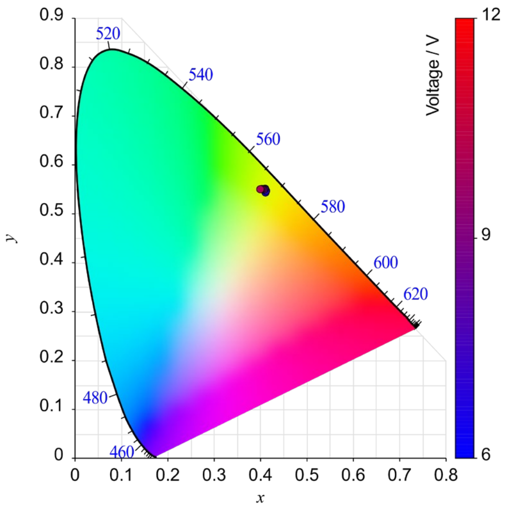

Figure S15. CIE 1931 Chromaticity Diagram showing the color points of LECs employing **2c** as the emitting material driven at different voltage values, from 7 V to 10 V.

7. References

1. Arnal, L.; Fuertes, S.; Martín, A.; Sicilia, V., The Use of Cyclometalated NHCs and Pyrazoles for the Development of Fully Efficient Blue Pt(II) Emitters and Pt/Ag Clusters. *Chem. Eur. J.* **2018,** *24*, 9377 – 9384.

2. Sicilia, V.; Arnal, L.; Escudero, D.; Fuertes, S.; Martín, A., Chameleonic Photo- and Mechanoluminescence in Pyrazolate-Bridged NHC Cyclometalated Platinum Complexes. *Inorg. Chem.* **2021,** *60*, 12274−12284.

3. Cotton, F. A.; Falvello, L. R.; Uson, R.; Fornies, J.; Tomas, M.; Casas, J. M.; Ara, I., Heterobinuclear PtAg Compounds with Platinum-Silver Bonds Unsupported by Covalent Bridges. Molecular Structure of $(C_6F_5)_3(SC_4H_8)PtAgPPh_3$. *Inorg. Chem.* **1987,** *26*, 1366-1370.

4. Frisch, M. J.; Trucks, G. W.; Schlegel, H. B.; Scuseria, G. E.; Robb, M. A.; Cheeseman, J. R.; Scalmani, G.; Barone, V.; Petersson, G. A.; Nakatsuji, H.; Li, X.; Caricato, M.; Marenich, A. V.; Bloino, J.; Janesko, B. G.; Gomperts, R.; Mennucci, B.; Hratchian, H. P.; Ortiz, J. V.; Izmaylov, A. F.; Sonnenberg, J. L.; Williams; Ding, F.; Lipparini, F.; Egidi, F.; Goings, J.; Peng, B.; Petrone, A.; Henderson, T.; Ranasinghe, D.; Zakrzewski, V. G.; Gao, J.; Rega, N.; Zheng, G.; Liang, W.; Hada, M.; Ehara, M.; Toyota, K.; Fukuda, R.; Hasegawa, J.; Ishida, M.; Nakajima, T.; Honda, Y.; Kitao, O.; Nakai, H.; Vreven, T.; Throssell, K.; Montgomery Jr., J. A.; Peralta, J. E.; Ogliaro, F.; Bearpark, M. J.; Heyd, J. J.; Brothers, E. N.; Kudin, K. N.; Staroverov, V. N.; Keith, T. A.; Kobayashi, R.; Normand, J.; Raghavachari, K.; Rendell, A. P.; Burant, J. C.; Iyengar, S. S.; Tomasi, J.; Cossi, M.; Millam, J. M.; Klene, M.; Adamo, C.; Cammi, R.; Ochterski, J. W.; Martin, R. L.; Morokuma, K.; Farkas, O.; Foresman, J. B.; Fox, D. J., Gaussian 16 Rev. C.01. **2016**.

5. Zhao, Y.; Truhlar, D. G., The M06 Suite of Density Functionals for Main Group Thermochemistry, Thermochemical Kinetics, Noncovalent Interactions, Excited States, and Transition Elements: Two New Functionals and Systematic Testing of Four M06-class Functionals and 12 Other Functionals. *Theor. Chem. Acc.* **2008,** *120*, 215-241.

6. Wang, Y.; Verma, P.; Jin, X.; Truhlar, D. G.; He, X., Revised M06 Density Functional for Main-Group and Transition-Metal Chemistry. *Proceedings of the National Academy of Sciences* **2018,** *115*, 10257.

7. Grimme, S.; Antony, J.; Ehrlich, S.; Krieg, H., A Consistent and Accurate Ab Initio Parametrization of Density Functional Dispersion Correction (DFT-D) for the 94 Elements H-Pu. *J. Chem. Phys.* **2010,** *132*, 154104.

8. Andrae, D.; Häußermann, U.; Dolg, M.; Stoll, H.; Preuß, H., Energy-adjusted ab Initio Pseudopotentials for the Second and Third Row Transition Elements. *Theor. Chim. Acta* **1990,** *77*, 123-141.

9. Ditchfield, R.; Hehre, W. J.; Pople, J. A., Self-Consistent Molecular-Orbital Methods. IX. An Extended Gaussian-Type Basis for Molecular-Orbital Studies of Organic Molecules. *J. Chem. Phys.* **1971,** *54*, 724-728.

10. Hariharan, P. C.; Pople, J. A., The Influence of Polarization Functions on Molecular Orbital Hydrogenation Energies. *Theor. Chim. Acta* **1973,** *28*, 213-222.

11. Tomasi, J.; Mennucci, B.; Cammi, R., Quantum Mechanical Continuum Solvation Models. *Chem. Rev.* **2005,** *105*, 2999-3094.

12. CrysAlis RED, Program for X-ray CCD camera data reduction (Version 1.171.32.19). Oxford Diffraction Ltd., Oxford, United Kingdom, 2008.

13 G.M. Sheldrick (2015) "Crystal structure refinement with SHELXL", Acta Cryst., C71, 3-8.

14 Spek, A. L., Single-crystal structure validation with the program PLATON. *J. Appl. Cryst.* **2003**, *36*, 7-13.

15. Falvello, L. R.; Forniés, J.; Martín, A.; Sicilia, V.; Villarroya, P., Synthesis and Reactivity of the Neutral Pyrazolate Complexes $[M_2\{CH_2C_6H_4P(o\text{-tolyl})_2\text{-}\kappa C,P\}_2(\mu\text{-Rpz})_2]$ (M = Pd, Pt; Rpz = Pz, 3,5-dmpz, 4-Mepz) toward $AgClO_4$. Molecular Structure of $[Pt_2Ag\{CH_2C_6H_4P(o\text{-tolyl})_2\text{-}\kappa C,P\}_2(\mu\text{-4-Mepz})_2]ClO_4$. *Organometallics* **2002,** *21*, 4604-4610.

16. Baya, M.; Belío, Ú.; Forniés, J.; Martín, A.; Perálvarez, M.; Sicilia, V., Neutral Benzoquinolate Cyclometalated Platinum(II) Complexes as Precursors in the Preparation of Luminescent Pt–Ag Complexes. *Inorg. Chim. Acta* **2015,** *424*, 136-149.

17. Aullón, G.; Alvarez, S., Axial Bonding Capabilities of Square Planar d_8-ML_4 Complexes. Theoretical Study and Structural Correlations. *Inorg. Chem.* **1996,** *35*, 3137-3144.

18. Cotton, F. A.; Falvello, L. R.; Uson, R.; Fornies, J.; Tomas, M.; Casas, J. M.; Ara, I., Heterobinuclear PtAg Compounds with Platinum-Silver Bonds Unsupported by Covalent Bridges. Molecular Structure of $(C_6F_5)_3(SC_4H_8)PtAgPPh_3$. *Inorg. Chem.* **1987,** *26*, 1366-1370.

19. Casas, J. M.; Falvello, L. R.; Forniés, J.; Martín, A., Direct Ag−Pt_2 Interactions in Pentafluorophenyl A-Frame Complexes Containing Halide or OH- and Bis(diphenylphosphino)methane (dppm) as Bridging Ligands. Crystal Structures of $[(C_6F_5)_2Pt(\mu\text{-}OH)(\mu\text{-}dppm)\{Ag(PPh_3)\}Pt(C_6F_5)_2]\cdot C_7H_8$ and $[(C_6F_5)_2Pt(\mu\text{-}SC_4H_8)(\mu\text{-}dppm)Pt(C_6F_5)_2]\cdot 1/2C_6H_{14}$. *Inorg. Chem.* **1996,** *35* (26), 7867-7872.
